# Multilevel Targets for Promoting Pediatric HPV Vaccination: A Systematic Review of Parent-Centered, Provider-Centered, and Practice-Centered Interventions in HIC and LMIC Settings

**DOI:** 10.3390/vaccines13030300

**Published:** 2025-03-11

**Authors:** Aaliyah Gray, Celia B. Fisher

**Affiliations:** 1 Center for Women’s and Gender Studies, Florida International University, Miami, FL 33199, USA; 2 Department of Psychology, Fordham University, Bronx, NY 10458, USA; fisher@fordham.edu; 3 Center for Ethics Education, Fordham University, Bronx, NY 10458, USA

**Keywords:** HPV, human papillomavirus, adolescents, childhood immunization, pediatric vaccine, strategies, parent, provider, practice, multilevel, intervention

## Abstract

Background/Objectives: Human papillomavirus (HPV) is a predominant cause of cervical cancer globally in women. HPV-related cancers in men are also on the rise. Immunization against HPV infection is a highly effective preventative against these cancers. However, HPV vaccine programs are not easily implemented globally. The objective of this systematic review was to identify multilevel strategies associated with improved pediatric HPV vaccination in high-income countries (HICs) and low- and middle-income countries (LMICs) that target parent, provider, and practice points of interventions. Methods: Through a systematic search of electronic databases, we identified 159 peer-reviewed articles published between the years 2011 and 2023. Results: Ninety-five percent of the included studies were conducted in HICs. Just eight studies were set in LMICs. A variety of HPV vaccination outcomes were assessed including uptake, initiation of the series, continuation of the series, missed opportunities, time, and refusal. Eighty percent of studies reported improved pediatric HPV vaccination, including a third of studies with mixed findings. Parent-centered strategies included education programs and reminder/recall procedures. Provider-centered strategies also included education programs and training in communication. Practice-centered strategies included vaccine access programs, vaccine bundling protocols, provider prompts, standing orders, vaccine messaging, and lowering the initiation age to 9 years. Multilevel, multi-component programs were highly effective. Conclusions: Multilevel strategies can be adopted in a variety of settings to promote HPV vaccination among youth globally. However, this research is disproportionately conducted in high resource environments. Further work is needed in LMIC settings as more countries begin to adopt HPV immunization programs.

## 1. Introduction

Human papillomavirus (HPV) is a sexually transmitted infection that nearly all people will acquire in their lifetime. HPV infection can resolve without symptoms, whereas those caused by high-risk HPV types are responsible for 5% of all cancers globally, including nearly 100% of cervical cancer and a growing incidence of cancers affecting men [1,2,3,4]. Low- and middle-income countries (LMICs) are most burdened by cervical cancer, representing 90% of incidence and mortality rates globally due to inequities in HPV- and HPV-related cancer prevention, screening, and treatment [2,5]. Likewise, the global incidence of HPV-related cancer among men, specifically anal and oropharyngeal cancers, is on the rise [6]. However, HPV-related cancer prevention and screening for males is underdeveloped globally [4,7]. Immunization against HPV is an instrumental preventative of HPV infection and precancerous lesions that can cause HPV-related cancers with ≥80% efficacy in reducing HPV-related outcomes and cancers as well as high levels of protection for at least 10 years [8]. Early immunization against HPV (i.e., ages 11 or 12 and as early as 9) is currently recommended [8,9].

The elimination of cervical cancer is an international priority. The 2020 World Health Organization Global Strategy to Accelerate the Elimination of Cervical Cancer as a Public Health Problem aims to include HPV vaccines in all national immunization programs with the goal of immunization among 90% of all girls worldwide by age 15 [10]. However, it is not well adopted globally. Just 27% of girls worldwide have initiated the vaccine [11], and only 5% of boys worldwide initiated the HPV vaccine series in 2019 [12]. A larger proportion of high-income countries (HICs) have started partial or national HPV immunization programs than LMICs as of November 2024. In 2019, only a third of programs were “gender-neutral” (i.e., vaccinating both boys and girls), with all programs located in HICs or upper-middle income countries and no gender-neutral programs in lower income countries [12].

A World Health Organization research priority for HPV immunization is the identification of strategies that can improve and sustain HPV vaccine uptake and series completion [11]. To improve success in the adoption of the HPV vaccine globally, a multilevel model of factors facilitating HPV vaccine uptake, series initiation, and completion is needed. Introduced by Bednarczyk and colleagues, the P3 model is a synthesis of health promotion and behavioral frameworks that outlines the ecology of a clinical intervention based on the interplay of three independent tiers directed at the patient, provider, and practice levels [13]. The patient level represents factors relevant to the immediate decision-maker such as a parent, the provider level represents factors relevant to those administering the vaccine, and the practice level represents systemic and procedural factors implemented through clinics or other health services. Other reviews of multilevel interventions have adopted different frameworks, such as the socioecological model [14] and the vaccine hesitancy model [15], that focus solely on cultural, social, and interpersonal factors. Reviews that have adopted the P3 model or similarly examined multilevel or multi-component interventions have highlighted knowledge, attitudes and beliefs, outcome expectations, intention to vaccinate at the parent level; recommendations for girls and boys to receive the HPV vaccine at the provider level; and targeting reminder/recall protocols for parents or provider-targeted procedures including assessment/feedback, education, and language services at the practice level [16,17,18]. However, these reviews have been limited in scope, focusing on research conducted solely in the United States or including a small number of studies. As such, an updated review of multilevel approaches drawing on evidence from the global literature on pediatric HPV vaccination is needed.

Guided by the P3 multilevel framework, the objective of this large-scale systematic review is to explore strategies and interventions implemented to improve the uptake of the HPV vaccine among pediatric patient populations across high-, middle-, and low-income areas. This review has the following three main aims:

(1) Apply the P3 multilevel framework to review and synthesize the published literature on targets in parent-centered, provider-centered, and practice-centered interventions associated with pediatric HPV vaccine initiation and uptake.
(2) Compare targets in high-income country (HIC) and low- and middle-income country (LMIC) settings.
(3) Evaluate the integration of parent-, provider-, and practice-level interventions that can increase HPV vaccination initiation, uptake, and completion in pediatric patient populations.

## 2. Methods

### 2.1. Study Protocol and Registration

This systematic review was conducted in accordance with the standards outlined by the Preferred Reporting Items for Systematic Reviews and Meta-analysis (PRISMA, see Supplementary Materials for PRISMA checklist) [19]. The study protocol was created by AG and CBF and registered with the International Prospective Register of Systematic Reviews (PROSPERO, #CRD42023476871) in October 2023. Following a pilot of the search procedures in November 2023, the final systematic database search was conducted in December 2023.

### 2.2. Search Strategy

An extensive search for peer-reviewed publications was conducted with seven online academic databases including CINAHL, Embase, Medline ProQuest, PubMed, PsycINFO, Scopus, and Web of Science. Keywords and Medical Subject Headings (MeSHs) were specified by AG following a modified PIO strategy to identify relevant search terms for the populations (parent, providers, practice/clinic), interest (interventions, strategies), outcomes (initiation, uptake, and completion of HPV vaccination), and context (pediatric HPV vaccination) of the systematic review. Table S1 in the Supplementary Materials provides the final list of search terms utilized in this review. The systematic and methodological search strategy was replicated across all seven databases.

### 2.3. Study Selection

#### 2.3.1. Inclusion and Exclusion Criteria

The objective of this systematic review was to identify interventions and strategies to improve pediatric HPV vaccination across multiple levels of intervention. A priori eligibility criteria were established prior to the preliminary systematic search and piloting of search procedures. To be included, studies needed to meet all of the following criteria: (1) publication years from 2008 to 2023; (2) empirical or original quantitative/qualitative data; (3) research designs including quasi-experimental, pre/post, cohort, case–control, cross-sectional, longitudinal, and clinical randomized control trial; and (4) research focused on modifiable factors across parent, provider, and practice levels including psychosocial, attitudinal, behavioral, procedural, economic, or systematic (minoritized populations, government, public health policies) factors. Articles were not considered if they were pre-prints, not peer-reviewed, other types of manuscripts including reviews, commentaries, abstracts, dissertations, or gray literature, focused on outcomes limited to HPV vaccine awareness, acceptability, or intention, or only focused on individuals 18 years or older. This review has a global scope and studies from all countries were eligible. Manuscripts not published in English were translated using Google Translate.

#### 2.3.2. Database Search

Database searches were conducted within all seven databases. Each database search consisted of the following: As outlined by the above search strategy, seven individual searches for each category of terms were conducted, and a final combined search query was implemented by joining the previous searches with the Boolean operator “AND” (Table 1). All search queries were limited to abstracts, publication titles, and keywords. Full citations including publication titles and abstracts were exported from each database in the RIS file format and imported to the Covidence systematic review workflow platform by AG. As citations were imported into Covidence, duplicates were automatically identified and removed by the software.

#### 2.3.3. Title and Abstract Screening

Using the Covidence systematic review workflow platform, two reviewers (AG and an undergraduate research assistant, MB) independently screened publication titles and abstracts for eligibility. Eligibility was determined based on perceived relevance to the research questions and adherence to the a priori inclusion and exclusion criteria. Disagreement was resolved through discussion and consensus between the two reviewers. At this stage, additional duplicates were manually identified and removed.

#### 2.3.4. Full-Text Review

Full text portable document format (PDF) files were retrieved for each citation that met eligibility criteria based on title and abstract screening. The two reviewers (AG and MB) independently reviewed the full text articles and determined eligibility based on the a priori inclusion and exclusion criteria. Disagreement was resolved through discussion and consensus between the two reviewers. Articles that failed to meet eligibility were excluded and the reasons for exclusion were documented. Eligible abstracts that did not have an accessible full text were excluded if all attempts to locate the full text were unsuccessful.

### 2.4. Data Extraction

Citations of the final set of articles were exported and managed using Endnote and Papers. AG and CBF developed the data extraction criteria. AG developed and completed the data extraction form in Microsoft Excel. The extracted data included the following: (1) publication information (author’s name, publication year, full citation); (2) study location/country; (3) race/ethnicity and socioeconomic status (annual income, employment status, insurance status, or parent education level) based on indicators from adolescent targets, their parents, or practice data; (4) gender, (5) age group, and (6) sample size of adolescent targets; (7) intervention level (parent, provider, practice, or multilevel); (8) sample size for intervention participants (parent, provider, and/or practices); (9) intervention setting/context; (10) intervention targets (modifiable factors); (11) description of the intervention or implemented strategies; (12) target outcome(s); (13) source of outcome data; and (12) summary of study findings.

## 3. Results

### 3.1. Study Selection

**Figure 1:**
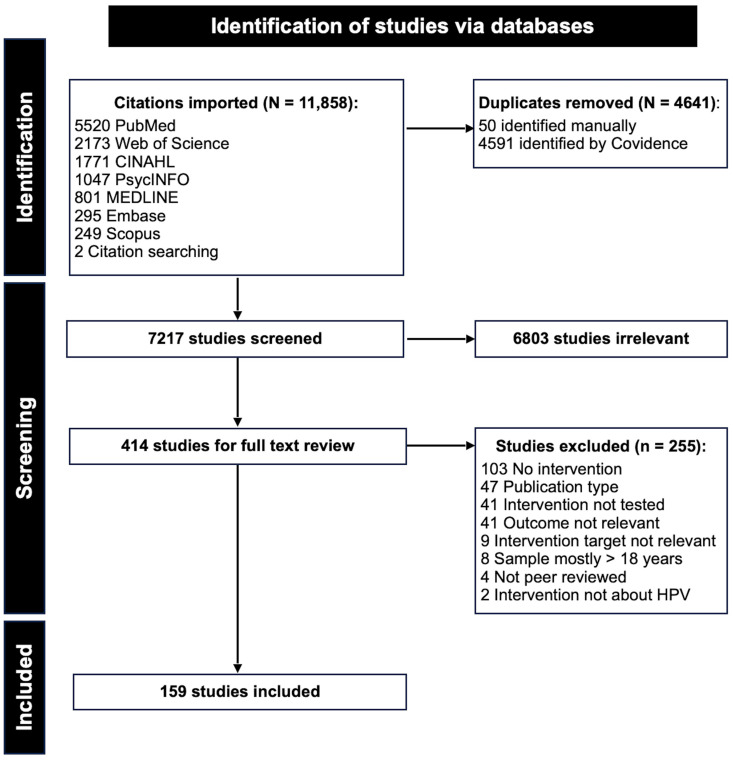
Flow diagram illustrating study selection process.

A summary of our study selection process, including the identification, screening, and inclusion stages, is illustrated with a PRISMA flow diagram in Figure 1. Nearly 12,000 citations were imported for screening. After the removal of 4641 duplicates, 7217 studies were screened based on titles and abstracts and 6803 were found to be irrelevant to our systematic review. The remaining 414 studies were retained for full-text review. At this stage of screening, 255 studies were excluded based on eligibility criteria. In total, 159 published studies were analyzed in our systematic review.

### 3.2. Risk-of-Bias Assessment

The 159 articles included in the final sample were evaluated for risk of bias in their methodology. Studies reporting the prevalence of HPV vaccination were evaluated using the Joanna Briggs Institute Critical Appraisal Checklist for Studies Reporting Prevalence Data [20], which included 9 questions assessing methodological concerns attributed to potential bias (Table S2 in Supplementary Materials). Quasi-experimental studies were evaluated with the Joanna Briggs Institute Critical Appraisal Checklist for Quasi-Experimental Studies [21], comprising 9 questions assessing biased results in non-randomized experimental studies (Table S3 in Supplementary Materials). Lastly, bias in studies with randomization was evaluated with the Joanna Briggs Institute Critical Appraisal Checklist for Assessment of Risk of Bias for Randomized Controlled Trials [22], comprising 13 questions (Table S4 in Supplementary Materials). AG conducted the evaluation for all studies. For each assessment, responses included “Yes”, “No”, Unclear”, or “Not Applicable”. The results of the risk-of-bias assessment were reviewed by CBF. Most studies were high quality with minimal methodological risk of bias. Studies with randomization demonstrated the most potential for bias. No study was removed from the final sample based on the quality criteria evaluated by each assessment.

### 3.3. Study Characteristics

Table S5 in the Supplementary Materials summarizes the key characteristics of the 159 studies included in our final sample. All studies were published between 2011 and 2023. Nearly all (95%, *n =* 151) were conducted in HICs in North America, particularly the United States. Other high-income settings included Europe (England, Italy, Denmark, the Netherlands, France, Sweden, Switzerland), the Middle East (Israel), East Asia (Japan), and Australia. Only 5% (*n* = 8) were conducted in LMICs in the Caribbean (Haiti), South America (Peru, Brazil, Bolivia), Africa (Cameroon, Lesotho, Uganda, Tanzania), South Asia (India, Bhutan, Nepal), and Southeast Asia (Cambodia, Malaysia). Nearly half (47%, *n* = 74) were quasi-experimental designs, 35% (*n* = 55) were randomized control trials, and 19% (*n* = 30) were prevalence studies. Among the eight LMIC studies, four were prevalence studies [23,24,25,26], two were quasi-experimental [27,28], and two were randomized control trials [29,30].

All studies focused on pediatric HPV vaccination among younger and older adolescents aged 9–17 years. Of these, a third (33%, *n* = 52) included adolescents as young as ages 9 or 10 among those eligible for vaccination. One study did not specify the age of their adolescent target population [31]. Most studies (67%, *n* = 107) focused on vaccination among both boys and girls and nearly a third (28%, *n* = 45) only examined vaccination among girls. Just four studies, all conducted in the United States, examined vaccination among boys only [32,33,34,35]. For studies in LMIC settings, six of the eight included adolescents aged 9 and 10 [23,24,25,27,28,29], and all eight focused on vaccination among girls only.

Studies were categorized by the intervention target: 21% focused on parents (*n* = 34), 11% on the provider (*n* = 17), 35% on the practice level (*n =* 55), and 33% were multilevel (*n* = 53). Of those with multilevel targets, 47% (*n* = 25) were focused on provider- and practice-level targets; 25% (*n* = 13) targeted the parent, provider, and practice levels; 17% (*n* = 9) were parent- and provider-level interventions; and 11% (*n* = 6) were parent- and practice-level interventions. For LMIC studies, seven of the eight were focused on practice-level targets [23,25,26,27,28,29,30] and just one was a multilevel intervention with parent, provider, and practice targets [24].

**Figure 2:**
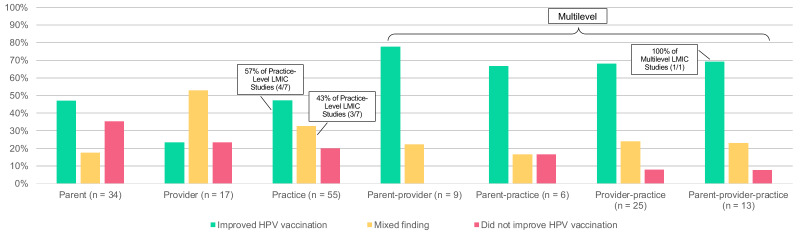
Efficacy of the study findings across single- and multilevel intervention targets. Results indicate a high prevalence of improvement in HPV across parent-, practice-, and multilevel studies; provider-level studies reported more mixed findings. LMICs stand for low- and middle-income countries of which 7 of 8 LMIC studies were practice level that reported improvement in HPV vaccination (*n* = 4, 57%) or mixed findings (*n* = 3, 43%), and 1 of 8 studies was multilevel that saw improved HPV vaccination.

Various outcomes were assessed in this review. About half the studies examined the uptake of any dose in the HPV vaccination series, vaccination rate (i.e., rate of uptake among eligible adolescents), or continuation of the HPV vaccine series (50%, *n* = 80), initiation of the series (i.e., first dose; 47%, *n* = 75), or completion of the series (45%, *n* = 72). Nine studies assessed missed opportunities for vaccination (i.e., clinical encounters where vaccine-eligible adolescents are not given the vaccine), three studies examined time periods related to vaccination (i.e., time between doses, time to completion, etc.), and two studies examined vaccine refusal. Approximately half (53%, *n* = 84) reported that the intervention improved HPV vaccination and nearly a third (28%, *n* = 44) reported mixed findings. About 20% (*n =* 31) found that the intervention did not improve vaccination. Of the eight LMIC studies, all reported positive [23,24,26,29,30] or mixed evidence of intervention success [25,27,28]. Figure 2 shows the efficacy of the study findings across single- and multilevel intervention targets, which shows a high prevalence of improvement in HPV vaccination across parent-, practice-, and multilevel studies, whereas provider-level studies reported more mixed findings.

## 4. Synthesis of Results

Table 1 characterizes all 159 studies by the intervention target, intervention level, and outcome (i.e., HPV vaccination is improved, not improved, or study reports mixed findings).

**Table 1:** Summary of intervention results on HPV vaccine uptake or completion across studies.

	Results of Intervention Target on HPV Vaccine Uptake or Completion		
Intervention Target	Improved	Mixed Findings	Did Not Improve
Parent knowledge/attitudes	Parent: [31,36,37,38,39,40,41,42,43,44,45,46,47] Multilevel: [24,32,48,49,50,51,52,53,54,55,56,57,58,59,60,61,62,63,64,65]	Parent: [66,67,68,69,70] Multilevel: [33,65,71,72,73,74]	Parent: [75,76,77,78,79,80,81,82,83,84,85] Multilevel: [34,86]
Parent motivations			Parent: [87]
Parent intention/decision-making	Parent: [88]		Multilevel: [86]
Communication with child/others	Parent: [31,41]	Parent: [66,68]	Parent: [81] Multilevel: [86]
Communication with provider	Parent: [89]	Parent: [90]	Parent: [77,79]
Parent self-efficacy	Parent: [89]	Parent: [90]	Parent: [79]
Health educator or Patient/peer navigators	Parent: [41,45,46] Multilevel: [91]	Parent: [67,68] Multilevel: [92]	
Reminder/recall	Parent: [36,93] Practice: [29,94,95,96,97,98,99,100,101,102,103,104,105] Multilevel: [50,54,59,60,62,63,65,106,107,108,109,110]	Parent: [67] Practice: [111,112,113,114,115,116,117,118] Multilevel: [65,72,119,120,121]	Parent: [82] Practice: [122,123,124] Multilevel: [125]
Patient invitation	Multilevel: [110]		
Provider education	Provider: [126] Multilevel: [24,35,48,51,52,53,58,59,60,61,62,63,64,91,106,107,108,109,110,127,128,129,130,131,132,133,134,135,136]	Provider: [137,138,139,140,141] Multilevel: [72,73,74,92,119,120,121,142,143]	Provider: [144,145,146] Multilevel: [34,125,147]
Performance feedback	Practice: [100,103,148] Multilevel: [60,63,64,107,129,131,135,136,149]	Provider: [140,150,151] Multilevel: [119,120,121]	Provider: [144,146,152] Practice: [153,154] Multilevel: [125]
Provider communication and/or recommendation style	Provider: [126,155,156,157] Multilevel: [32,48,49,50,51,52,53,58,59,60,61,64,106,108,109,127,128,129,130,131,135,136,149]	Provider: [137,138,139,141,150,158,159] Multilevel: [33,71,120,121,143]	Provider: [145,146,152] Multilevel: [34,147]
Vaccine timing/schedule	Practice: [23]		
Vaccine access	Practice: [102,105,160] Multilevel: [24,54,55,56,57,61,110]	Practice: [28,117,161,162] Multilevel: [72,74]	Practice: [163,164]
Provider prompts	Practice: [26,98,100,101,165,166,167,168,169,170] Multilevel: [35,59,64,106,107,128,132,133,136,149]	Practice: [27,112,117,161,162,171,172,173] Multilevel: [143]	Practice: [123,154,164,174,175] Multilevel: [34,125]
Patient prompts	Practice: [168] Multilevel: [127,133,134]		
Patient invitation	Practice: [176] Multilevel: [110]	Practice: [172]	Practice: [175]
Immunization registry	Practice: [100,103]		Practice: [124]
Patient navigators	Practice: [177] Multilevel: [91]	Multilevel: [92]	
Vaccine procedures	Multilevel: [64,108,109,127,128,130,132,133,134]	Practice: [161] Multilevel: [119,142]	Practice: [123] Multilevel: [34,86,125,147]
Consent procedures	Practice: [105]	Practice: [178]	Practice: [179]
Patient comfort	Practice: [103]		Practice: [180]
Patient incentives	Practice: [99]		
Program subsidies	Practice: [95]		
Initiation age	Practice: [30,165,181] Multilevel: [58,61,64,129,135]	Practice: [25,161,173] Multilevel: [74,143]	Practice: [123]
Standing orders	Practice: [100,101,103,148,166] Multilevel: [54,64,65,106,108,109,128,136,149]	Practice: [161,173] Multilevel: [34,65,119,121]	Practice: [123,124]
Vaccine messaging	Practice: [181]	Provider: [161]	
Clinic materials	Multilevel: [62,64,130,136,149]	Practice: [172] Multilevel: [72,73,142,143]	
Media/advertisement campaign	Practice: [26] Multilevel: [54,60]		Practice: [164]

### 4.1. Parent-Centered Interventions

Table S6 in Supplementary Materials provides a summary of the 34 studies assessing interventions conducted only at the parent level. All studies were conducted in HICs (United States, Switzerland, Denmark, the Netherlands, Japan, Israel), and assessed HPV vaccination in girls (*n =* 15) or boys and girls (*n =* 19). Nine studies included younger adolescents aged 9 and 10 in their target population. Most studies were randomized control trials (*n* = 19), 10 were quasi-experimental, and 5 were prevalence studies. HPV vaccine outcomes included uptake (*n* = 15), initiation (*n* = 21), and completion of the series (*n* = 13). A total of eight targets to improve HPV vaccination at the parent level were identified. Most studies targeted parent knowledge and attitudes (*n* = 29). Other targets were parent motivations (*n* = 1), parent intentions and decision-making (*n* = 1), communication between child and parent (*n* = 5), communication between provider and parent (*n* = 4), parent self-efficacy (*n* = 3), patient/peer navigators (*n* = 5), and reminder/recall protocols (*n* = 4).

Sixteen of the thirty-four parent-level studies reported improvement in uptake, initiation, or completion of the HPV vaccine series, with six additional studies reporting mixed results. However, twelve studies found no improvement in any HPV vaccine outcomes. Across these studies, improving parent knowledge and attitudes through comprehensive, culturally-sensitive educational sessions utilizing health educators and peer/patient navigators was one of several promising points of intervention. For example, a 60-min educational presentation from local health educators was associated with 44% of adolescents initiating the series [46]. More intensive health educator programs have shown even greater effects with a program comprising four group sessions and one home-visit individual session associated with the following: 8× greater odds of uptake of dose 2, 6× greater odds of initiation, and 16× greater odds of completion than the comparison group [45]; and one-on-one sessions combined with reminder/recall text messages similarly associated with improved initiation and completion [36]. When possible, technology such as websites, mobile and tablet applications, and videos can be beneficial. One study team utilized Facebook to post about the HPV vaccine in parenting groups and reported an 8% increase in vaccine initiation and 15% increase in series completion [37], and another intervention using the Vacteens/Vacunadolescente.org mobile application and website was associated with a 20% increase in initiation and 37% increase in completion [47]. Likewise, parents using tablets demonstrated greater odds of uptake [39] with one tablet intervention featuring a nurse avatar and virtual conversation module for parents resulting in same-day in-office initiation among 50% of adolescent patients [38]. Informational video interventions were associated with 13–14% increases in uptake among girls and a 9% increase in uptake among boys [42,43]. One intervention paired videos with health educators, resulting in 485× higher odds of vaccine initiation, with 3% of initiators completing the series compared to zero percent in the control group [41]. Interventions that included increasing adolescent knowledge directly or through communication with parents were also successful [66,68,69,70,90]. However, greater efforts to include parents in improving HPV vaccination in LMICs are needed, as all parent-centered studies identified in this review were conducted in HICs.

### 4.2. Provider-Centered Interventions

A summary of the 17 studies assessing interventions conducted only at the provider level is included in Table S7 in the Supplementary Materials. All studies were conducted in the United States and assessed vaccination in both boys and girls. Four studies (24%) included the vaccination of younger adolescents aged 9 and 10 years. Eight studies were quasi-experimental, six were randomized control trials, and three were prevalence studies. HPV vaccine outcomes included the uptake of any dose (*n* = 9), initiation of the first dose (*n* = 10), completion of the series (*n* = 5), and missed opportunities to administer the vaccine (*n* = 3). Four targets for provider-centered interventions were identified across studies including provider education (*n* = 9), performance feedback (*n* = 6), provider communication with parents (*n =* 11), and recommendation style (*n* = 8).

Of the 17 total studies, 4 were associated with improved HPV vaccination, 4 were not, and 11 were inconclusive. Interventions targeting providers were largely focused on provider communication with parents or strong recommendations. Motivational interviewing techniques were associated with increased initiation of the vaccine series [157] and strong recommendations utilizing presumptive, announcement, or indicated messaging (e.g., “your child is due for the HPV vaccine”) have been associated with increased initiation [126,155], 9× greater odds of uptake [156], and a decrease in missed opportunities [126]. Provider education on HPV epidemiology and key information about the vaccine (e.g., side effects, benefits, efficacy, etc.) is also necessary for supporting strong recommendations to parents [126,137,138,139,140,141]. Providers must also be prepared to continue conversations with parents over time, especially after an initial refusal of the vaccine for their child. An observational study of provider responses to parents refusing to vaccinate found that various “active responses” from the provider such as giving more information, offering to talk about the HPV vaccine again at a later visit, trying to change the parent’s mind during the visit, or asking the parent to sign a form confirming refusal to vaccinate were not associated with uptake after refusing the vaccine, whereas follow-up behaviors including scheduling another visit to talk about the vaccine again, discussing the vaccine again at the next visit, or sending a reminder via phone, text, email, or mail were associated with higher odds of uptake after the initial decline [159]. Providers may also benefit from regular performance feedback [140,150,151], although practice-level systems capable of providing data on performance to providers would be required. A limitation of these findings is that all studies were conducted in the United States. Consequently, research on provider-centered practices outside of the United States in other HIC settings and in a variety of LMICs is needed to understand how providers can be used to improve vaccination in other environments.

### 4.3. Practice-Centered Interventions

Findings from the 55 studies assessing interventions only targeting aspects of practice (i.e., health care setting) are included in Table S8 in Supplementary Materials. All but seven studies were conducted in HICs including countries of North America (United States, Canada; *n* = 39), Europe (Denmark, England, Italy, Sweden; *n* = 7), Japan (*n* = 1), and Australia (*n* = 1). The seven studies in LMICs were conducted in countries of the Caribbean (Haiti, *n* = 2), South America (Brazil, Bolivia, Peru; *n* = 3), Africa (Cameroon, Lesotho, Uganda, Tanzania; *n* = 3), South Asia (Bhutan, Nepal; *n* = 1), and Southeast Asia (Cambodia, Malaysia; *n* = 2). Most studies (62%, *n* = 34) assessed vaccination in boys and girls, and a third (33%, *n* = 18) assessed vaccination in girls only. Sixteen studies (29%) included adolescents aged 9 and 10 years in their sample. Twenty-six studies were quasi-experimental, eighteen were randomized control trials, and eleven were prevalence studies. Several HPV vaccine outcomes were assessed across studies including the uptake of any dose (*n* = 32), initiation of the first dose (*n* = 19), completion of the series (*n* = 23), missed opportunities (*n* = 3), refusing vaccination (e.g., noncompletion after dose 1, *n =* 1), and time (e.g., time between doses, time to completion, etc., *n* = 4). We identified 20 practice-level targets. At least 10% of studies implemented reminder/recall protocols (*n =* 24), provider prompts (*n =* 24), vaccine access programs (*n =* 9), standing orders (*n =* 9), initiation age changes (*n =* 7), and/or performance feedback programs (*n =* 6) as a part of their interventions. A minority of studies (e.g., 1–3 studies per target) included other targets such as implementing patient prompts, patient/peer navigator programs, and media/advertisement campaigns; utilizing patient invitations, clinic materials, patient comfort tactics, patient incentives, and program subsidies; and modifying vaccine timing, vaccine procedures, consent procedures, provider vaccine communication/recommendation scripts, and vaccine messaging.

Of the 55 practice-level studies, 26 (47%) reported improvement in HPV vaccination, 18 (33%) reported mixed results, and 11 (20%) demonstrated no significant improvement. Effective practice-level interventions include vaccine access methods (school-based programs, in particular), provider prompts, standing orders, and reminder/recalls. School-based vaccination programs demonstrated high levels of uptake and completion (60–99%) [26,27,102,105,160,164,167]. School-based programs should consider vaccination based on school grade [25,30]. Similarly, a clinic-based intervention that implemented a vaccine schedule corresponding to the local school calendar reported that 98% of adolescent girls initiated the vaccine, 92% received dose 2, and 25% completed the series following the intervention [23]. Provider prompts were highly effective [169], especially when combined with other practice-level strategies [168,170]. One study found that provider prompts and standing orders together contributed to 4× greater initiation, 3× greater completion, and 4× faster decrease in missed opportunities than pre-intervention rates [166]. Methods of reminder/recall included mailed letters, phone calls, and text messages. Reminder/recall interventions were associated with greater vaccine uptake and completion than standard care uptake [29,94,96,104,176], and phone, email, and text reminders promoted on-time vaccination [97,98].

When paired with reminder/recall methods, incentives (e.g., cash or vaccine subsidies) were associated with increased uptake and completion [95,99]; but these strategies may not be tenable for low-resource settings or those that do not have already-established subsidized vaccine programs. Lowering the initiation age was also effective for improving vaccination. One study found that changing provider prompts to alerts for patients aged 9 years and to flag the HPV vaccine as “due now” rather than “optional” was associated with a 99% increase in initiation [165]. Likewise, recommending the vaccine for patients aged 9 through to parents and focusing on the cancer prevention benefits of the vaccine was associated with a 35% increase in the rate of initiation for adolescents aged 9–10 years and a 15% increase for adolescents aged 11–12 years [181]. Utilizing multiple practice-level strategies was common, and particularly important for success with using performance feedback as a strategy to improve vaccination [100,101,103,148,153].

However, practice-centered interventions alone may require support from other levels of intervention. For example, provider adherence to the systems and procedures implemented at the practice-level is necessary for sustainability of interventions by modifying provider prompts, initiation age, and standing orders. Likewise, parental acceptability and support are required for the success of programs, improving vaccine access and other practice-level strategies such as reminder/recall messages. However, a limitation of these findings is the underrepresentation of research conducted in LMIC settings. More work is needed to explore the capacity of LMIC practices to implement effective interventions promoting HPV vaccine initiation and completion.

### 4.4. Multilevel Interventions

A total of 53 studies assessed interventions targeting two to three levels of intervention. All but one study were conducted in HICs including the United States (*n* = 49), Italy (*n* = 1), France (*n* = 1), and Australia (*n* = 1). The one LMIC study was conducted in India. Most studies (71%, *n* = 38) assessed vaccination in boys and girls with 20% (*n* = 11) assessing vaccination in girls only and 4 studies assessing vaccination in boys only. Nearly half (43%, *n* = 23) included adolescents aged 9 and 10 years. Twenty-nine studies were quasi-experimental, twelve were randomized control trials, and eleven were prevalence studies. Most studies (72%, *n* = 38) saw significant improvement in vaccination, 11 studies found mixed results, and just 4 found no significant improvement. Multilevel studies included 9 parent–provider interventions, 6 parent–practice interventions, 25 provider–practice interventions, and 13 parent–provider-practice interventions. The findings of all multilevel interventions are reported in Table S9 in Supplementary Materials.

#### 4.4.1. Parent–Provider Interventions

Nine studies focused on interventions targeting both parents and health care providers. All studies were conducted in the United States and were quasi-experimental with the exception of one randomized control trial. Half of the studies assessed vaccination in boys and girls; however, two focused on girls only and two focused on boys only. Four of the studies included younger adolescents 9 and 10 years of age. All studies targeted parent knowledge/attitudes paired with a provider-targeted strategy. Half also targeted provider communication and/or recommendation style. Four studies also focused on provider education. Just one study included a reminder/recall component in the intervention. Most assessed initiation (*n* = 5) and completion (*n* = 6) of the HPV vaccine series, with three studies examining uptake and one study examining missed opportunities.

#### 4.4.2. Parent–Practice Interventions

There were six parent- and practice-level interventions where both parent-centered strategies and strategies targeting the health care setting were implemented. Four of these were conducted in the United States, one in France, and one in Australia. Half of the studies were quasi-experimental, two were randomized control trials, and one was a prevalence study. Half focused on vaccination among boys and girls, and the other half focused on girls only. Just two studies explicitly reported recruiting parents of younger adolescents 9 and 10 years old. All studies targeted parent knowledge/attitudes. Most (*n* = 4) also targeted vaccine access. A minority of studies (i.e., 1–2 studies per target) included targets such as parent intention/decision-making, parent communication with child/others, implementing reminder/recalls, vaccine procedures, standing orders, and media/advertisement campaigns. One intervention examined uptake, with the remaining studies examining both the initiation and completion of the HPV vaccine series.

#### 4.4.3. Provider–Practice Interventions

There were 25 provider- and practice-level interventions where both strategies aimed at health care providers and strategies aimed at modifying aspects of the health care setting were implemented. All studies were conducted in the United States. About half were quasi-experimental, seven were randomized control trials, and six were prevalence studies. All but two studies examined vaccination in boys and girls; one study focused on girls only, and the other on boys only. Eight studies included younger adolescents 9 and 10 years old. Most interventions targeted provider education on HPV education on HPV infection and vaccination (*n* = 24), provider communication with parents or recommendation (*n* = 16), and vaccine procedures (i.e., vaccine bundling) (*n* = 12). Several interventions also implemented reminder/recall procedures (*n* = 8), provider prompts (*n* = 10), standing orders (*n* = 8), performance feedback (*n* = 10), and clinic materials (*n* = 5). A minority reported on patient navigator programs (*n* = 2), patient prompts (*n* = 3), and initiation age (*n* = 3). Most studies examined HPV vaccine uptake (*n* = 12) and series completion (*n* = 14), with fewer studies examining initiation (*n* = 9). Just two studies examined missed opportunities and one study examined refusal to take the vaccine.

#### 4.4.4. Parent–Provider–Practice Interventions

A total of 13 studies conducted multilevel interventions with parent, provider, and practice targets. All but one study was conducted in HICs, namely the United States (*n* = 11) and Italy (*n* = 1). The one LMIC study was conducted in India. Half of the studies were quasi-experimental, four were prevalence studies, and one was a randomized control trial. A variety of practice settings were included such as school-based clinics, community health clinics, academic hospitals, primary care settings, immunization clinics, and local health departments/offices. Half examined vaccination in boys and girls; five focused on girls only and one on boys only. Half of the studies also recruited younger adolescents aged 9 and 10 years. All studies targeted parent knowledge/attitudes (*n =* 13) and provider education (*n* = 13). Other parent-level and provider-level targets were patient invitation (*n* = 1) and provider communication/recommendation (*n* = 6). Several practice-level targets were examined including reminder/recall (*n* = 6), vaccine access (*n* = 5), initiation age (*n* = 4), provider prompts (*n* = 3), vaccine procedures (*n* = 2), performance feedback (*n =* 3), standing orders (*n* = 2), clinic materials (*n* = 4), and media/advertisement campaigns (*n* = 1). Outcomes of interest were the uptake of any dose (*n* = 8), initiation of the series (*n* = 6), and completion of the series (*n* = 6).

#### 4.4.5. Evidence Supporting Intervention Across Multilevel Targets

Synergies of parent–provider, parent–practice, provider–practice, and parent–provider–practice interventions demonstrated high-levels of effectiveness in improving HPV vaccination. This is particularly promising since provider-level interventions alone were less effective than parent-only and practice-only interventions. Multiple-strategy, multilevel interventions provide flexibility for HPV vaccination improvement programs across a variety of settings and resource levels.

Seven (78%) of the nine parent–provider studies found that the intervention significantly improved HPV vaccination among adolescents and the remaining two studies found mixed results. Parent–provider strategies consisted of provider education combined with parent-targeting websites [49], fact sheets [50], booklets [52,53], direct MyChart messages to parents [51], games [48], or media campaigns promoting adolescent safety and health behaviors [32,33]. All four (67%) of the six parent–practice interventions that improved HPV vaccination focused on promoting parent knowledge/attitudes and vaccine access to adolescents of which three were school-based and associated with increased initiation and completion [55,56,57], and one was based in an authorized “Vaccines for Children” pharmacy, demonstrating a 7% increase in HPV vaccination [54]. Similar to the parent–provider strategies, 16 of the 17 (68%) successful provider–practice interventions targeted provider education on HPV combined with other approaches, including patient navigators [92] and patient [133] and provider prompts [35]. Combinations between provider education and multiple practice-level strategies were highly effective. These studies paired provider education with other intervention targets including performance feedback and provider recommendation training [120,131], vaccine procedures (e.g., vaccine bundling) and provider prompts [108,109,127,128,130,132,134], and provider communication approaches and initiation age [129,135]. Likewise, nine (69%) of the thirteen parent-provider–practice study interventions improved HPV vaccination, with an additional three interventions reporting mixed results. These studies implemented several evidence-based interventions described in this review to create comprehensive, multilevel programs that can be sustained over time to improve vaccination among adolescents.

**Figure 3:**
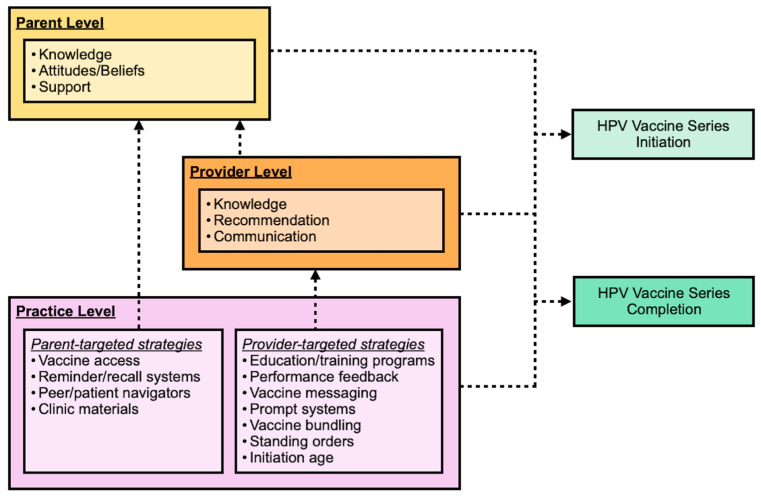
Multilevel model of HPV pediatric vaccine facilitators. Adapted from Rodriguez et al. 2020 [16].

Overall, our results indicate that effective targets for a multilevel program include the implementation of educational programs for parents and the utilization of reminder/recalls and clinic materials to support adherence to appointments and parent acceptance of the vaccine. Likewise, education programs for providers are needed. Provider training should include HPV epidemiology, vaccine effectiveness, and strategies for improving communication with parents and adolescents using standard vaccine messaging and strong recommendations. Programs must also provide opportunities for vaccination by establishing access to the vaccine in school-based or community settings. Access to the vaccine in clinical settings is best supported by improved vaccine procedures such as bundling the vaccine with other childhood immunizations and lowering the initiation age, standing orders allowing the administration of the vaccine by nurses, and provider prompts which alert providers to adolescents needing the vaccine. As depicted in Figure 3, an integrated intervention across multilevel intervention targets maximizes the extent to which HPV vaccine initiation and series completion can be improved through intervention programs. However, more research examining multilevel intervention in general, and particularly in LMIC settings, is urgently needed.

## 5. Discussion

Currently, just a third of girls and 5% of boys worldwide have been vaccinated against HPV infection [11,12]. The objective of this large-scale systematic review was to identify multilevel, intervention targets for improving pediatric HPV vaccination and to synthesize findings across the existing global literature in this area. Identifying strategies that promote HPV vaccination is an important research priority of the World Health Organization [8]. We conducted a novel, comprehensive review of 159 articles from HIC and LMIC settings, identifying facilitators and barriers to HPV vaccination for adolescents. Following the P3 model [13], this review considers the role of parents, providers, and practices in the uptake of the HPV vaccine for adolescent populations. We found that a majority of strategies were successful. In total, 80% of all studies reported improved HPV vaccination among youth, including half that reported only positive results and a third of studies that reported mixed findings.

Consequently, practice- or provider-centered approaches to improving vaccine uptake, initiation, and completion are limited without parental support [182]. Parents must find the vaccine acceptable, which requires them to have an adequate level of knowledge about HPV and the vaccine and to possess positive attitudes about immunization against the virus. However, lack of awareness, knowledge, and acceptance of the vaccine constitute major parent-level barriers to pediatric HPV immunization. Targeting parent knowledge is thus among the most important and common strategies used to promote HPV vaccination [18]. In our review of parent-level interventions, we found that 2 in 3 studies reported improvement in HPV vaccine uptake. Consistent with other reviews, these parent-centered strategies are associated with improved HPV vaccination among youth [15,16,17,183,184,185]. Strategies that were most successful addressed vaccine hesitancy by targeting parent knowledge and attitudes about HPV and HPV vaccination through one-on-one counseling with providers, patient/peer navigators, community educators, or various technological methods such as tablets, videos, websites, and applications, and supporting adherence through reminder/recall messages.

As evident in our review, provider knowledge and communication skills also play an important role in parent acceptance of the vaccine. Providers are a trusted source of information and care, requiring them to possess adequate knowledge of the vaccine and the capacity to provide a strong recommendation to parents. For example, low vaccine uptake has been associated with low HPV knowledge among providers [186], and disparities in provider recommendations for the HPV vaccine disproportionately impact uptake among boys, younger adolescents, ethnic and racial minority individuals, lower income individuals, and those in rural areas [187,188]. Consistent with prior research, in our review, provider training in HPV epidemiology, communication with parents, vaccine messaging, and presumptive recommendations are necessary for the uptake of the vaccine, initiation, and completion of the vaccine series [15,16,17,189,190,191,192], and providers benefit from webinars, telemonitoring, and train-the-trainer programs [18]. However, provider-level interventions alone are not sufficient despite being among the most implemented strategies [18]. Although 3 in 4 studies reported improvement in vaccination in our review, over half of these reported mixed results. Consequently, systems at the practice level are necessary for the implementation and success of vaccine programs.

Practice-level interventions were highly effective alone; 5 in 6 studies in our review reported improvement in vaccination with just a third reporting mixed findings. Although previous work has found system- and practice-level interventions to be less commonly implemented than other types of strategies [17,18], they were the most endorsed level of strategy in our review. Similar to earlier reports [16], these practice-level strategies included targeting parents through vaccine access programs, reminder/recall procedures, peer and patient navigator programs, and clinic materials (e.g., brochures, posters, etc.). Successful practice-level strategies targeting providers included education programs, performance feedback, practice-wide vaccine messaging, bundling of the vaccine with other pediatric immunizations, provider prompt systems, standing orders authorizing nurse administration of the vaccine, and lowering the initiation age to 9 years.

Reflecting the multilevel P3 paradigm, our review and other recent work [193] suggest that approaches to improve vaccination are best served by multilevel interventions. We found that 93% of multilevel intervention studies reported improvement in HPV vaccination. Prior work has identified several barriers to implementing multilevel interventions including cost, time, and difficulties integrating interventions into the current workflow infrastructure [14,194]. However, consistent with prior work [194], we found that multilevel systems can be adapted to a variety of settings, in particular LMICs, as these strategies do not necessarily rely on high levels of technology at the practice level. For example, parent-targeted components can successfully include school- and community-based vaccine access programs embedded into existing school and community infrastructure. Paper records and vaccine cards can act as reminder/recalls for parents, and community health workers can conduct follow-up visits with adolescents. Likewise, peer and patient navigators can conduct community-based education programs for parents [14,17]. In clinical settings, nurse-led prompts for providers can be implemented instead of electronic or state-level record systems. As promising as these multilevel interventions are, our review and others [195] found that single-level and multilevel strategies are disproportionately implemented in HICs as compared to LMICs, indicating the need for further work that identifies appropriate strategies for low-resource settings.

Emerging evidence demonstrating the success of HPV immunization suggests several HICs are on target to eliminate cervical cancer in the near future. For example, Australia is anticipated to be the first country to reduce cervical cancer incidence below the elimination threshold through HPV vaccination and screening programs [196], data from Scotland shows no cervical cancer in girls vaccinated between the ages of 12 and 13 years [197], and Norway similarly reports no cervical cancer in 25-year-old women due to the introduction of the vaccine in the pediatric national immunization program [198]. By contrast, many LMICs are lagging in reaching targets for elimination due to insufficient programming and limited resources. Of the 159 studies included in our review, just 8 were conducted in LMICs in the Caribbean, Africa, South America, South Asia, and Southeast Asia. Many of these areas (e.g., sub-Saharan Africa, South America, the Caribbean, and Southeast Asia) report the highest incidence of cervical cancer in the world and related mortality, and experience greater inequities in HPV-related cancer prevention, screening, and treatment [2,5,8]. Some LMIC regions, such as North Africa and Asia, report low vaccine coverage compared to areas like sub-Saharan Africa, the latter demonstrating higher coverage despite the limited introduction of HPV vaccination programs [12]. Given disparities in vaccination rates among low-resourced settings, a better understanding of these vaccine programs is warranted. Rwanda, for example, has rapidly developed their vaccine program to become the first African country anticipated to eliminate cervical cancer [199]. Between 2011 and 2018, 98% of the target population initiated the HPV vaccine series with coverage among 9 in 10 girls aged 12 years [200]. Today, Rwanda maintains one of the highest vaccination rates globally [201]. The Rwandan Ministry of Health attributes their success to a culturally-informed health education program that relies on village elders, community health workers, churches, and schools [201]. The importance of culturally-sensitive, community-embedded, and evidence-based vaccine strategies has been widely discussed in previous work within a variety of low-resource communities and settings [15,184,202,203,204]. This underscores the urgent need to support research on facilitators and barriers to pediatric HPV vaccination in LMICs.

In our review, we found that no LMIC study included boys in their target population. By contrast, 70% of HIC studies included boys. Currently, adolescent boys and adult men are not a primary target population in global pediatric HPV immunization efforts [8]. Just a third of programs are gender-neutral with most implemented in higher income settings [12]. Given the rise in HPV-related cancer among men [6], gender-neutral vaccination efforts will become increasingly important in efforts to curb HPV-related cancers in men as well as cervical cancer in women. Assessing the implementation of vaccine programs and multilevel strategies to promote vaccination among boys in LMICs is another necessary area of research moving forward.

Our review identified several limitations in this literature. As previously noted, HPV vaccination research is disproportionately conducted in HICs and under-representative of LMICs. Further, despite our more stringent exclusion criteria, upon detailed review, some studies suffered from small and potentially biased samples, limiting the generalizability of these studies to other populations. The strengths of our review include the number of eligible studies examined and the geographic diversity of our sample. Even though we were only able to identify a few studies in LMICs, we are one of a few multilevel reviews to have a global scope and a large sample of articles. Lastly, as a systematic review, our study benefited from the construction of a reproducible, rigorous methodology to ensure it adequately reflected empirical knowledge in this area.

## 6. Conclusions

This systematic review identified various intervention targets across parent, provider, and practice levels associated with HPV vaccine uptake, initiation, and series completion in HIC and LMIC settings. The diversity of interventions described in this comprehensive review provides a clear perspective of effective strategies for promoting HPV vaccination and current barriers in a variety of settings, with several implications for promoting HPV vaccination among global youth populations. Key strategies identified in our review included the following: parent-level education targeting attitudes and knowledge; provider-level education targeting HPV knowledge and communication, practice-level systems to support parent decision-making and provider training to encourage vaccination through reminder/recall procedures, vaccine bundling, standing order protocols, and provider prompts. Our review also identified school-based and community-based settings as common venues for vaccine promotion. However, these strategies cannot exist in isolation. Our review indicates the high level of success of multi-component, multilevel interventions in promoting HPV vaccination across a variety of settings. Our findings also highlight several areas of improvement for clinical practice. For example, few studies included 9–10-year-olds in their vaccination targeting. Greater efforts to introduce the vaccine early in adolescence must be made in both HIC and LMIC settings. Additionally, efforts to target adolescent boys in LMIC vaccine programs are necessary but lacking. To understand which multilevel programs benefit adolescents most, longitudinal and causal work is needed to determine which intervention effects are sustained over time. Despite the progress that has been made in evidence-based strategies to reduce HPV vaccine hesitancy, gains in successful multilevel strategies have largely benefited those living in HICs. Increased research conducted in LMICs remains of international importance and will be essential in creating a global resource for best practices that can be adopted in low-resource clinical and community settings as more countries adopt the HPV vaccine.

## Data Availability

No new data were created or analyzed in this study.

## Supplementary Materials

The following supporting information can be downloaded at: https://www.mdpi.com/article/10.3390/vaccines13030300/s1, PRISMA Checklist; Table S1: Categories of Search Terms; Table S2: Risk of Bias for Prevalence Studies; Table S3: Risk of Bias for Quasi-Experimental Studies; Table S4: Risk of Bias for Studies with Randomization; Table S5: Summary of Study Characteristics; Table S6: Summary of Parent-Level Studies; Table S7: Summary of Provider-Level Studies; Table S8: Summary of Practice-Level Studies; Table S9: Summary of Multilevel Studies. References [19,23,24,25,26,27,28,29,30,31,32,33,34,35,36,37,38,39,40,41,42,43,44,45,46,47,48,49,50,51,52,53,54,55,56,57,58,59,60,61,62,63,64,65,66,67,68,69,70,71,72,73,74,75,76,77,78,79,80,81,82,83,84,85,86,87,88,89,90,91,92,93,94,95,96,97,98,99,100,101,102,103,104,105,106,107,108,109,110,111,112,113,114,115,116,117,118,119,120,121,122,123,124,125,126,127,128,129,130,131,132,133,134,135,136,137,138,139,140,141,142,143,144,145,146,147,148,149,150,151,152,153,154,155,156,157,158,159,160,161,162,163,164,165,166,167,168,169,170,171,172,173,174,175,176,177,178,179,180,181] are cited in the Supplementary Materials.
