# Supplementary material for: Multilevel Targets for Promoting Pediatric HPV Vaccination: A Systematic Review of Parent-Centered, Provider-Centered, and Practice-Centered Interventions in HIC and LMIC Settings
Source: Vaccines (Basel). 2025 Mar 11;13(3):300. doi: 10.3390/vaccines13030300 (PMC11945458; doi:10.3390/vaccines13030300)
Supplement: Supplementary file 1 [file vaccines-13-00300-s001.zip › supplementary materials.pdf]

Multilevel Targets for Promoting Pediatric HPV Vaccination: A Systematic Review of Parent-Centered, Provider-Centered, and Practice-Centered Interventions in HIC and LMIC Settings

Aaliyah Gray and Celia B. Fisher

2025, *Vaccines*

***Refer to the published article for the proper citation**

Supplementary Materials

Table of Contents

Supplementary Table S1 – Categories of Search Terms 2

Supplementary Table S2 - Risk of Bias for Prevalence Studies 3

Supplementary Table S3 - Risk of Bias for Quasi-Experimental Studies 4

Supplementary Table S4 - Risk of Bias for Studies with Randomization 7

Supplementary Table S5 - Summary of Study Characteristics 9

Supplementary Table S6 - Summary of Parent-Level Studies 12

Supplementary Table S7 - Summary of Provider-Level Studies 23

Supplementary Table S8 - Summary of Practice-Level Studies 29

Supplementary Table S9 - Summary of Multilevel Studies 45

Supplementary Table S1. Categories of Search Terms.

PIO Component	Category	Search query
**Population**	**Parent**	parent OR parental OR guardian OR mother OR father OR grandparents OR family
	**Providers**	provider OR doctor OR practitioner OR physician OR nurses OR health care provider OR health care professional OR health care doctor OR health care practitioner OR health care physician OR primary care provider OR primary care doctor OR primary care practitioner OR family provider OR family doctor OR family practitioner OR family physician OR pediatric doctor OR pediatric care provider OR pediatric practitioner OR pediatric physician OR nurse practitioners OR family nurse practitioners OR pediatric nurse practitioners OR paediatric doctor OR paediatric care provider OR paediatric practitioner OR paediatric physician OR paediatric nurse practitioners
	**Practice/clinic**	practice OR clinic OR clinical care OR health care setting OR community health OR health services OR school health services OR health organization OR clinical practice OR health care practice
		AND
**Interest**	**Intervention, strategies**	intervention OR strategy OR strategies OR prevent OR prevention OR program OR programme OR promotion OR modifiable OR modify OR policy
		AND
**Outcome**	**Initiation, uptake, completion**	vaccinate OR vaccine OR vaccination OR immunize OR immunise OR immunization OR immunisation OR prophylaxis OR uptake OR completion OR complete OR compliance OR coverage
		AND
**Context**	**Pediatric, 9-17 years HPV vaccine**	child OR children OR childhood OR pediatric OR paediatric OR adolescent OR adolescence OR teen OR teenage OR youth OR school age OR juvenile OR student
		AND
		human papilloma virus OR HPV OR papillomaviridae OR papilloma OR papillomavirus OR human papilloma virus vaccine OR human papilloma virus vaccination OR HPV vaccine OR HPV vaccination OR papillomavirus vaccine OR papillomavirus vaccination

Supplementary Table S2. Risk of Bias for Prevalence Studies (N = 30).

	Joanna Briggs Institute Critical Appraisal Checklist for Studies Reporting Prevalence Data								
References	1. Was the sample frame appropriate to address the target population?	2. Were study participants sampled in an appropriate way?	3. Was the sample size adequate?	4. Were the study subjects and the setting described in detail?	5. Was the data analysis conducted with sufficient coverage of the identified sample?	6. Were valid methods used for the identification of the condition?	7. Was the condition measured in a standard, reliable way for all participants?	8. Was there appropriate statistical analysis?	9. Was the response rate adequate, and if not, was the low response rate managed appropriately?
Abuelo et al., 2014 [23]	●	●	×	●	●	●	●	●	●
Ahmed et al., 2022 [24]	●	●	●	●	●	●	●	●	●
Austin & Morgan, 2019 [144]	●	●	△	●	●	△	●	●	●
Berenson et al., 2019 [91]	●	●	●	●	●	●	●	●	●
Bonville et al., 2019 [106]	●	●	●	●	●	●	●	●	●
Brodie & McPeak, 2018 [126]	●	●	●	●	●	●	●	●	●
Calo et al., 2019 [163]	●	●	△	●	●	●	●	●	●
Casey et al., 2022 [129]	●	●	●	●	●	●	●	●	●
Charles & Erikson, 2023 [130]	●	●	×	●	●	△	●	●	●
Chen et al., 2017 [38]	●	●	△	●	●	●	●	●	●
Cox et al., 2022 [61]	●	●	●	●	●	●	●	●	●
Daniel et al., 2021 [54]	●	●	×	●	●	●	●	●	●
Fujiwara et al., 2013 [95]	●	●	●	●	●	△	●	●	●
Giambi et al., 2015 [110]	●	●	△	●	●	△	●	●	●
Groom et al., 2017 [153]	●	●	△	●	●	△	●	●	●
Hansen et al., 2020 [40]	●	●	●	●	●	●	●	●	●
Huang et al., 2024 [64]	●	●	△	●	●	●	●	●	●
Jacobs-Wingo et al., 2017 [123]	●	●	●	●	●	●	●	●	●
Mackey et al., 2019 [172]	●	●	×	●	●	●	●	●	●
Margolis et al., 2022 [159]	●	●	●	●	●	●	●	●	●
Mugisha et al., 2015 [25]	●	●	●	●	●	△	●	●	●
Muhamad et al., 2018 [26]	●	●	●	●	●	△	●	●	●
Obulaney et al., 2016 [42]	●	●	△	●	●	△	●	●	●
Oliver et al., 2021 [161]	●	●	△	●	●	●	●	●	●
Potts & Southard, 2019 [43]	●	●	×	●	●	△	●	●	●
Rand et al., 2015 [114]	●	●	●	●	●	△	●	●	●
Rodriguez et al., 2022 [102]	●	●	●	●	●	●	●	●	●
Smajlovic & Toth, 2023 [149]	●	●	△	●	●	●	●	●	●
Spleen et al., 2012 [46]	●	●	△	●	●	●	●	●	●
Zorn et al., 2023 [143]	●	●	●	●	●	●	●	●	●

● = Yes, × = No, △ = Unclear

Supplementary Table S3. Risk of Bias for Quasi-Experimental Studies (N = 74).

	Joanna Briggs Institute Critical Appraisal Checklist for Quasi-Experimental Studies								
References	1. Is it clear in the study what is the “cause” and what is the “effect” (i.e. there is no confusion about which variable comes first)?	2. Was there a control group?	3. Were participants included in any comparisons similar?	4. Were the participants included in any comparisons receiving similar treatment/care, other than the exposure or intervention of interest?	5. Were there multiple measurements of the outcome, both pre and post the intervention/ exposure?	6. Were the outcomes of participants included in any comparisons measured in the same way?	7. Were outcomes measured in a reliable way?	8. Was follow-up complete and if not, were differences between groups in terms of their follow-up adequately described and analyzed?	9. Was appropriate statistical analysis used?
Agana-Norman et al., 2022 [32]	●	●	●	●	●	●	●	●	●
Aragones et al., 2015 [36]	●	●	●	●	×	●	●	●	●
Bae et al., 2017 [171]	●	●	●	●	×	●	●	●	●
Bar-Shain et al., 2015 [112]	●	●	●	●	×	●	●	●	●
Beck et al., 2021 [88]	●	●	●	●	●	●	●	●	●
Berenson et al., 2020 [92]	●	●	●	●	●	●	●	●	●
Bernstein et al., 2022 [127]	●	●	●	●	●	●	●	●	●
Biehl & Efre, 2023 [128]	●	●	●	●	●	●	●	●	●
Bowden et al., 2017 [58]	●	●	●	●	●	●	●	●	●
Brewer et al., 2021 [155]	●	●	●	●	●	●	●	●	●
Buller et al., 2021 [37]	●	●	●	●	●	●	●	●	●
Cassidy et al., 2014 [59]	●	●	●	●	●	●	●	●	●
Cates et al., 2018 [48]	●	●	●	●	●	●	●	●	●
Cates et al., 2014 [33]	●	●	●	●	●	●	●	●	●
Cates et al., 2011 [71]	●	●	●	●	×	●	●	●	●
Chantler et al., 2020 [179]	●	●	●	●	×	●	●	●	●
Choi et al., 2018 [60]	●	●	●	●	●	●	●	●	●
Christensen et al., 2023 [165]	●	●	●	●	●	●	●	●	●
Chung et al., 2015 [72]	●	●	●	●	●	●	●	●	●
Coley et al., 2018 [77]	●	●	●	●	●	●	●	●	●
Dang et al., 2023 [62]	●	●	●	●	●	●	●	●	●
Davis et al., 2022 [147]	●	●	●	●	●	●	●	●	●
Dawson et al., 2018 [138]	●	●	●	●	●	●	●	●	●
Dempsey et al., 2019 [158]	●	●	●	●	×	●	●	●	●
Deshmukh et al., 2018 [166]	●	●	●	●	●	●	●	●	●
Desiante et al., 2017 [160]	●	●	●	●	×	●	●	●	●
Fenton et al., 2021 [156]	●	●	●	●	×	●	●	●	●
Fiks et al., 2016 [139]	●	●	●	●	●	●	●	●	●

Fisher et al., 2022 [178]	●	●	●	●	●	●	●	●	●
Froidevaux et al., 2023 [78]	●	●	●	●	●	●	●	●	●
Fuller & Papachrisanthou, 2017 [177]	●	●	●	●	●	●	●	●	●
Glenn et al., 2023 [96]	●	●	●	●	×	●	●	●	●
Goleman et al., 2018 [181]	●	●	●	●	●	●	●	●	●
Howhü & Bro, 2012 [176]	●	●	●	●	×	●	●	●	●
Irving et al., 2018 [146]	●	●	●	●	●	●	●	●	●
Jafari et al., 2020 [50]	●	●	●	●	×	●	●	●	●
Kaul et al., 2019 [55]	●	●	●	●	●	●	●	●	●
Kharbanda et al., 2011 [98]	●	●	●	●	●	●	●	●	●
Krantz et al., 2018 [132]	●	●	●	●	●	●	●	●	●
Ladner et al., 2012 [27]	●	●	●	●	×	●	●	●	●
Ma et al., 2022 [41]	●	●	●	●	×	●	●	●	●
Marchand-Ciriello et al., 2020 [34]	●	●	●	●	●	●	●	●	●
McLean et al., 2017 [120]	●	●	●	●	●	●	●	●	●
Meyer et al., 2018 [133]	●	●	●	●	●	●	●	●	●
Molokwu et al., 2019 [67]	●	●	●	●	●	●	●	●	●
Molokwu et al., 2023 [35]	●	●	●	●	●	●	●	●	●
Moore et al., 2022 [134]	●	●	●	●	●	●	●	●	●
Moss et al., 2012 [100]	●	●	●	●	●	●	●	●	●
Musto et al., 2013 [175]	●	●	●	●	×	●	●	●	●
O'Leary & Frost, 2023 [173]	●	●	●	●	●	●	●	●	●
Oliver et al., 2019 [124]	●	●	●	●	●	●	●	●	●
Parra-Medina et al., 2015 [68]	●	●	●	●	●	●	●	●	●
Perkins et al., 2021 [101]	●	●	●	●	●	●	●	●	●
Poscia et al., 2019 [167]	●	●	●	●	●	●	●	●	●
Pratt et al., 2021 [44]	●	●	●	●	●	●	●	●	●
Rand et al., 2018 [121]	●	●	●	●	●	●	●	●	●
Rand et al., 2018 [136]	●	●	●	●	●	●	●	●	●
Real et al., 2022 [157]	●	●	●	●	●	●	●	●	●
Rehn et al., 2016 [164]	●	●	●	●	●	●	●	●	●
Riviere et al., 2021 [28]	●	●	●	●	×	●	●	●	●
Ruffin et al., 2015 [168]	●	●	●	●	●	●	●	●	●
Sanderson et al., 2017 [74]	●	●	●	●	×	●	●	●	●
Staras et al., 2020 [90]	●	●	●	●	●	●	●	●	●
Steiner et al., 2021 [142]	●	●	●	●	●	●	●	●	●
Strasel et al., 2024 [51]	●	●	●	●	●	●	●	●	●
Stubbs et al., 2014 [56]	●	●	●	●	×	●	●	●	●
Suryadevara et al., 2019 [52]	●	●	●	●	●	●	●	●	●
Suryadevara et al., 2021 [53]	●	●	●	●	●	●	●	●	●

Szilagyi et al., 2020 [116]	●	●	●	●	✕	●	●	●	●
Szilagyi et al., 2021 [141]	●	●	●	●	●	●	●	●	●
Taddio et al., 2024 [180]	●	●	●	●	✕	●	●	●	●
Tran et al., 2022 [57]	●	●	●	●	✕	●	●	●	●
Vinci et al., 2022 [117]	●	●	●	●	●	●	●	●	●
Whelan et al., 2014 [105]	●	●	●	●	●	●	●	●	●

● = Yes, ✕ = No, △ = Unclear

Supplementary Table S4. Risk of Bias for Studies with Randomization (N = 55).

	Joanna Briggs Institute Critical Appraisal Checklist for Assessment of Risk of Bias for Randomized Controlled Trials												
References	1. Was true random-ization used for assignment of partici-pants to treatment groups?	2. Was allocation to treat-ment groups concealed?	3. Were treatment groups similar at the baseline?	4. Were participants blind to treatment assign-ment?	5. Were those delivering the treatment blind to treatment assign-ment?	6. Were treatment groups treated identically other than the inter-vention of interest?	7. Were outcome assessors blind to treatment assign-ment?	8. Were outcomes measured in the same way for treatment groups?	9. Were outcomes measured in a reliable way?	10. Was follow up complete and if not, were differences between groups in terms of their follow up adequately described and analy-zed?	11. Were partici-pants analyzed in the groups to which they were random-ized?	12. Was appro-priate statistical analysis used?	13. Was the trial design appropriate and any deviations from the standard RCT design (individual randomization, parallel groups) accounted for in the conduct and analysis of the trial?
Baldwin et al., 2021 [87]	●	△	●	△	●	●	△	●	●	**NA**	×	●	●
Bastani et al., 2022 [75]	●	△	●	△	△	●	△	●	●	●	●	●	●
Bradley-Ewing et al., 2022 [152]	●	△	●	△	△	●	△	●	●	●	●	●	●
Brewer et al., 2017 [137]	●	×	●	×	×	●	×	●	●	●	×	●	●
Chao et al., 2015 [94]	●	△	●	△	△	●	△	●	●	●	●	●	●
Chodick et al., 2021 [76]	●	△	●	△	△	●	△	●	●	●	●	●	●
Davies et al., 2023 [86]	●	△	●	△	△	●	△	●	●	●	●	●	●
Dempsey et al., 2018 [49]	●	×	●	●	×	●	×	●	●	●	●	●	●
Dixon et al., 2019 [39]	●	△	●	△	△	●	△	●	●	●	●	●	●
Ferreira et al., 2022 [29]	●	△	●	●	△	●	△	●	●	●	×	●	●
Fiks et al., 2013 [63]	●	●	●	●	●	●	●	●	●	●	●	●	●
Fiks et al., 2023 [150]	●	△	●	△	△	●	△	●	●	●	●	●	●
Fisher-Borne et al., 2018 [125]	×	△	●	△	△	●	△	●	●	●	●	●	●
Gilkey et al., 2014 [151]	●	△	●	△	△	●	△	●	●	●	●	●	●
Gilkey et al., 2022 [131]	●	×	●	×	×	●	×	●	●	●	●	●	●
Gilkey et al., 2023 [148]	●	×	●	×	×	●	×	●	●	●	●	●	●
Gilkey et al., 2019 [145]	●	×	●	×	×	●	×	●	●	●	●	●	●
Glenn et al., 2022 [119]	●	△	●	△	△	●	△	●	●	●	●	●	●
Gurfinkel et al., 2021 [111]	●	△	●	△	△	●	△	●	●	●	●	●	●
Hanley et al., 2023 [113]	●	△	●	△	△	●	△	●	●	●	●	●	●
Henrikson et al., 2018 [118]	●	△	●	△	△	●	△	●	●	●	×	●	●
Hofstetter et al., 2017 [122]	●	△	●	△	△	●	●	●	●	●	×	●	●

Joseph et al., 2016 [79]	●	✕	●	✕	✕	●	✕	●	●	●	●	●	●
Kempe et al., 2016 [97]	●	△	●	△	△	●	△	●	●	●	●	●	●
Koskan et al., 2022 [174]	●	△	●	△	△	●	△	●	●	●	●	●	●
Lee et al., 2018 [80]	●	△	●	●	●	●	△	●	●	●	✕	●	●
Mantzari et al., 2015 [99]	●	△	●	△	△	●	△	●	●	●	●	●	●
Maria et al., 2021 [66]	●	△	●	△	●	●	●	●	●	●	●	●	●
Mayne et al., 2014 [107]	●	△	●	△	△	●	△	●	●	●	✕	●	●
Paskett et al., 2016 [73]	●	△	●	△	△	●	△	●	●	●	✕	●	●
Perkins et al., 2020 [135]	●	△	●	△	△	●	△	●	●	●	✕	●	●
Perkins et al., 2015 [140]	●	△	●	△	△	●	△	●	●	●	●	●	●
Pot et al., 2017 [81]	●	△	●	△	△	●	△	●	●	●	●	●	●
Pot et al., 2020 [31]	●	△	●	△	△	●	△	●	●	●	●	●	●
Rand et al., 2023 [154]	●	△	●	△	△	●	△	●	●	●	●	●	●
Rand et al., 2017 [115]	●	△	●	✕	△	●	●	●	●	●	●	●	●
Richman et al., 2019 [82]	●	△	●	●	●	●	△	●	●	●	●	●	●
Rickert et al., 2015 [83]	●	△	●	△	✕	●	△	●	●	●	●	●	●
Rutten et al., 2024 [103]	●	△	●	●	●	●	△	●	●	●	●	●	●
Scarinci et al., 2020 [45]	●	△	●	△	△	●	△	●	●	●	●	●	●
Shegog et al., 2022 [84]	●	△	●	△	△	●	△	●	●	●	✕	●	●
Staras et al., 2015 [89]	●	△	●	△	△	●	△	●	●	●	✕	●	●
Suzuki et al., 2022 [85]	●	●	●	●	●	●	●	●	●	●	✕	●	●
Szilagyi et al., 2015 [162]	●	△	●	△	△	●	△	●	●	●	●	●	●
Tiro et al., 2015 [65]	●	△	●	△	●	●	△	●	●	●	●	●	●
Tull et al., 2019 [104]	●	✕	●	●	✕	●	✕	●	●	●	●	●	●
Underwood et al., 2019 [69]	●	△	●	△	△	●	△	●	●	●	●	●	●
Watson-Jones et al., 2012 [30]	●	✕	●	✕	✕	●	✕	●	●	●	●	●	●
Wilkinson et al., 2019 [169]	●	△	●	△	△	●	△	●	●	●	●	●	●
Winer et al., 2016 [70]	●	△	●	△	△	●	△	●	●	●	●	●	●
Woodall et al., 2021 [47]	●	✕	●	△	●	●	✕	●	●	●	●	●	●
Wynn et al., 2021 [93]	●	✕	●	✕	✕	●	●	●	●	●	●	●	●
Zimet et al., 2018 [170]	●	△	●	△	△	●	△	●	●	●	●	●	●
Zimmerman et al., 2017 [108]	●	△	●	△	△	●	△	●	●	●	●	●	●
Zimmerman et al., 2017 [109]	●	△	●	△	△	●	△	●	●	●	●	●	●

● = Yes, ✕ = No, △ = Unclear

Supplementary Table S5. Summary of Study Characteristics (N = 159).

Study Characteristic	Frequency (%)	References
**Study Setting**		
**High-Income Country (HIC)**	**151 (95%)**	
**North America**		
United States	130	[32–39, 41–56, 58–75, 77, 79, 80, 82–84, 87–94, 96–98, 100–103, 106–109, 111–159, 161–163, 165, 166, 168–174, 177, 181]
Canada	3	[105, 175, 180]
**Europe**		
England	3	[99, 178, 179]
Italy	3	[110, 160, 167]
Denmark	2	[40, 176]
The Netherlands	2	[31, 81]
France	1	[57]
Sweden	1	[164]
Switzerland	1	[78]
**Middle East**		
Israel	1	[76]
**East Asia**		
Japan	2	[85, 95]
**Oceania**		
Australia	2	[86, 104]
**Low- or Middle-Income Country (LMIC)**	**8 (5%)**	
**Caribbean**		
Haiti	2	[27, 28]
**South America**		
Peru	1	[23]
Brazil	1	[29]
Bolivia	1	[27]
**Africa**		
Cameroon	1	[27]
Lesotho	1	[27]
Uganda	1	[25]
Tanzania	1	[30]
**South Asia**		
India	1	[24]
Bhutan	1	[27]
Nepal	1	[27]
**Southeast Asia**		
Cambodia	1	[27]
Malaysia	1	[26]
**Study Type**		
Quasi-Experimental Study	74 (47%)	[27, 28, 32–37, 41, 44, 48, 50–53, 55–60, 62, 67, 68, 71, 72, 74, 77, 78, 88, 90, 92, 96, 98, 100, 101, 105, 112, 116,

		117, 120, 121, 124, 127, 128, 132–134, 136, 138, 139, 141, 142, 146, 147, 155–158, 160, 164–168, 171, 173, 175–181]
Randomized Control Trial	55 (35%)	[29–31, 39, 45, 47, 49, 63, 65, 66, 69, 70, 73, 75, 76, 79–87, 89, 93, 94, 97, 99, 103, 104, 107–109, 111, 113, 115, 118, 119, 122, 125, 131, 135, 137, 140, 145, 148, 150–152, 154, 162, 169, 170, 174]
Prevalence Study	30 (19%)	[23–26, 38, 40, 42, 43, 46, 54, 61, 64, 91, 95, 102, 106, 110, 114, 123, 126, 129, 130, 143, 144, 149, 153, 159, 161, 163, 172]
**Adolescent Targets**		
**Age**		
Includes ages 9 and/or 10 years	52 (33%)	[23–25, 27–29, 33, 35, 36, 42, 43, 45, 46, 48, 51, 54–58, 60, 61, 64, 67, 70, 71, 73, 74, 82, 91–94, 98, 102, 113, 118, 126, 129, 133, 135, 143, 149, 152, 156, 159, 165, 168, 173–175, 181]
Includes any ages 11-17 years	155 (98%)	[23, 24, 26–30, 32–50, 52–164, 166–181]
Includes ≥ 18 years	34 (21%)	[27, 34, 35, 41, 42, 44, 50, 52–54, 60, 65, 71, 72, 74, 78, 85, 94, 98, 99, 112, 113, 116, 129, 133, 134, 138, 140, 144, 151, 153, 166, 168, 171]
**Gender**		
Boys and Girls	107 (67%)	[35, 36, 38, 39, 41, 43, 44, 48, 49, 51–53, 55, 57, 58, 60–62, 64, 66, 67, 69, 72, 75, 77, 78, 82–84, 86–89, 91–93, 96, 100–104, 106, 108, 109, 111–125, 127–133, 135–162, 165, 167, 169–174, 180, 181]
Girls Only	45 (28%)	[23–31, 37, 40, 42, 45–47, 50, 54, 56, 59, 63, 65, 68, 70, 71, 73, 76, 79–81, 85, 90, 94, 95, 98, 99, 105, 107, 110, 164, 166, 168, 175, 176, 178, 179]
Boys Only	4 (3%)	[32–34, 134]
Not Reported	3 (2%)	[126, 163, 177]
**Intervention Targets**		
Parent-Level Only	34 (21%)	[31, 36–47, 66–70, 75–85, 87–90, 93]
Provider-Level Only	17 (11%)	[126, 137–141, 144–146, 150–152, 155–159]
Practice-Level Only	55 (35%)	[23, 25–30, 94–105, 111–118, 122–124, 148, 153, 154, 160–181]
Multilevel	53 (33%)	
Parent-Provider	9	[32, 33, 48–53, 71]
Parent-Practice	6	[54–57, 65, 86]
Provider-Practice	25	[35, 91, 92, 106–109, 119–121, 125, 127–136, 142, 143, 147, 149]
Parent-Provider-Practice	13	[24, 34, 58–64, 72–74, 110]
**HPV Vaccine Outcome**		
Uptake	80 (50%)	[23, 24, 27–31, 33, 39, 40, 42–45, 51, 54, 58–60, 63, 71, 73, 74, 77–79, 81, 83, 85, 88, 90, 92, 95–98, 102–104, 107, 110, 112–115, 117, 120, 122, 123, 125, 127, 128, 130, 132–134, 136, 138–142, 145, 152, 153, 156, 158, 159, 162–164, 167, 169–171, 173, 177–180]

Initiation (of the series)	75 (48%)	[23, 24, 27–31, 33, 39, 40, 42–45, 51, 54, 58–60, 63, 71, 73, 74, 77–79, 81, 83, 85, 88, 90, 92, 95–98, 102–104, 107, 110, 112–115, 117, 120, 122, 123, 125, 127, 128, 130, 132–134, 136, 138–142, 145, 152, 153, 156, 158, 159, 162–164, 167, 169–171, 173, 177–180]
Completion (of the series)	72 (45%)	[25–28, 32, 35–37, 41, 45, 47–50, 52, 53, 55–57, 59–62, 65–68, 70, 72, 74, 75, 77, 83, 86, 91, 93, 94, 97, 99–101, 105, 106, 108, 109, 111, 113, 115, 116, 118–121, 124, 129, 131, 132, 135, 137, 139, 143, 144, 146, 149, 151, 161, 166, 168, 172–175]
Missed Opportunities	9 (6%)	[33, 106, 126, 128, 141, 150, 154, 162, 166]
Time	4 (3%)	[115, 118, 162, 168]
Refusing Vaccination	2 (1%)	[147, 160]
**Result of Intervention**		
Improved HPV Vaccination	84 (53%)	[23, 24, 26, 29–32, 35–64, 88, 89, 91, 93–110, 118, 126–136, 148, 149, 155–157, 160, 165–170, 176, 181]
Mixed Findings	44 (28%)	[25, 27, 28, 33, 65–74, 90, 92, 111–117, 119–121, 137–142, 150, 151, 158, 159, 161, 162, 171–173, 177, 178]
Did Not Improve HPV Vaccination	31 (20%)	[34, 75–87, 122–125, 144–147, 152–154, 163, 164, 174, 175, 179, 180]

Supplementary Table S6. Summary of Parent-Level Studies (N = 34).

		Adolescent Targets		Intervention/Strategy			
Reference	Study Location/ Setting	Gender & Age	Race* & SES*	Modifiable Targets	Procedures/Description	HPV Vaccine Outcome	Findings
**Results indicate intervention associated improved HPV vaccine outcomes**							
Aragones et al., 2015 [36]	United States<br><br>Health Window at the New York City Mexican Consulate	N = NR<br>Boys and girls<br>9-17 years	100% Hispanic<br>SES = NR	N = 69 parents<br><br>Parent knowledge/attitudes, reminder/recall	Parents received one-on-one education and were encouraged to make an appointment with their child's provider or go to an immunization clinic to receive the vaccine. The first 24 parents were enrolled in the non-text messaging arm of the study (education only), and the following 45 parents received additional text messages once a week reminding them of their child's vaccination eligibility (education + text).	Initiation, completion of 3 doses	Initiation was significantly higher for adolescents in the education + text message condition (82%) compared to education only (40%).<br><br>Completion was significantly higher for adolescents in the education + text message condition (88%) compared to education only (40%). The education + text message was 15.5x more likely to complete the series.
Beck et al., 2021 [88]	United States<br><br>Nurse practitioner–run primary care and walk-in clinic in rural Southeast state	N = 24<br>Boys and girls<br>11-17 years	NH White = 75%<br>Hispanic = 21%<br>Other = 4%<br><br>Parent education<br>Less than high school diploma = 17%<br>High school diploma = 25%<br>Some college = 21%<br>College degree or high = 37%	N = 24 parents<br><br>Parent intention/decision-making	During health care appointments, parents completed the Parent Attitudes about Childhood Vaccine measure then received a strong recommendation for the HPV vaccine from their child' health care provider. Concerns were addressed based on parents' results on the Parent Attitudes about Childhood Vaccine measure. This process was repeated at the next office visit for parents who refused the vaccine.	Initiation, continuation of series	24 adolescents were vaccinated post-intervention, compared to 4 pre-intervention.
Buller et al., 2021 [37]	United States<br><br>Facebook	N = 869<br>Girls<br>14-17 years	NH White = 82%<br><br>Annual household income >$80000 = 51%	N = 869 parents<br><br>Parent knowledge/attitudes	Parents in two private Facebook groups received a social media intervention which comprised of researchers posting two times a day over 12 months about HPV and general vaccinations (e.g., benefits of influenza vaccination).	Initiation, completion	Significantly more daughters received at least 1 dose (71% vs. 63%) and completed the vaccine with two or three doses (65% vs. 50%) following the intervention compared to initiation and completion reported at baseline.

Chen et al., 2017 [38]	United States<br><br>Vaccine clinic in Arizona	N = NR<br>Boys and girls<br>11-17 years	Hispanic = 100%<br>Parent born in Mexico = 55%<br>Parent born in U.S. = 19%<br><br>Parent employment<br>Full time = 45%<br>Part-time = 5%<br>Unemployed = 50%	N = 42 parents<br><br>Parent knowledge/ attitudes	Parents received a virtual intervention featuring a nurse avatar. The program utilized a conversation tree and turn-based system and provided culturally-tailored information for Latino parents.	Initiation	Following the intervention, 50% of adolescents received same-day vaccination.
Dixon et al., 2019 [39]	United States<br><br>Five urban, safety-net health clinics of the Eskenazi Health system in Indianapolis, Indiana	N = 1596<br>Boys and girls<br>11-17 years	NH Black = 55%<br>Other/unknown = 37%<br>NH White = 9%<br><br>Health insurance<br>Public = 78%	N = 1596 parents<br>547 (intervention, 1059 (control)<br><br>Parent knowledge/ attitudes	Based on immunization status of the adolescents, parents interacted with one of two software programs on a study-provided tablet. If the adolescent had never received an HPV vaccine and were interested, the program reinforced the decision to initiate the vaccine series and provided information specific to the cancer prevention benefits and safety, if needed. If the adolescent already began the vaccine series, the program emphasized completing the vaccine series. Adolescents at control practices received usual care.	Uptake	The odds of receiving any dose was significantly higher for adolescents in the intervention than in the control.
Hansen et al., 2020 [40]	Denmark<br><br>National Campaign	N = 328,779<br>Girls<br>12-16 years	Race = NR<br>SES = NR	N = NR<br><br>Parent knowledge/ attitudes	This retrospective study examined historical contexts of population level vaccine rates. The first time period (baseline) was when HPV vaccine uptake was the same as other childhood vaccines. The second and third time period marked increasing levels of negative press about the vaccine. The fourth time period was the implemented information campaign aimed at raising uptake to 90%.	Uptake	Following the information campaign, rates of uptake were >90% and were not significantly different from rates at baseline (before the periods of negative media coverage).

Ma et al., 2022<br><br>[41]	United States<br><br>Community health centers that predominantly serve low-income Chinese American community in Philadelphia	N = 180<br>Boys and girls<br>11-18 years	Race = NR<br>SES = NR	N = 180 parents<br>110 (intervention), 70 (control)<br><br>Parent knowledge/attitudes, communication with child, health educator or patient/peer navigator	Parents received a culturally-tailored intervention that addressed concerns of Chinese parents. The intervention had four parts: one video with four modules demonstrating a routine vaccine injection, a conversation about the vaccine between two parents, communication between parent and child, and a conversation between two adolescents; a second video featuring an interview with a well-known pediatrician; a discussion with a health educator; and, printed materials. The control group received a general health intervention.	Initiation, completion of 3 doses	Significantly more adolescents in the intervention group (66%) initiated and completed the vaccine series compared to the control group (3%, initiated; 0% completed). The odds of initiation was 485x higher for the intervention group.
Obulaney et al., 2016 [42]	United States<br><br>Low-cost, faith-based clinic serving a large southern urban area	N = NR<br>Girls<br>9-18 years	Hispanic = 67%<br>NH White = 26%<br>NH Black = 7%<br><br>Parent education<br>Less than high school = 26%<br>High school diploma = 41%<br>Some college = 33%	N = 41 parents<br><br>Parent knowledge/attitudes	During an 20-25 minute educational session, parents watched a video and were provided a brochure and list of HPV preventive measures.	Vaccine uptake	Following the intervention, the rate of vaccine uptake increased from 5% to 18%.
Pot et al., 2020<br><br>[31]	Netherlands<br><br>Online	N = 3995<br>Girls<br>Age = NR	Race = NR<br>SES = NR	N = 3995 parents<br><br>Parent knowledge/attitudes, communication with child	Mothers interacted with a website that provided tailored feedback with two virtual assistants (a doctor and a mother). The website content included information about the HPV vaccine, pros and cons, how and where to get the HPV vaccine, and talking about the vaccine with their daughter or partner. Additionally, mothers could visit "in-depth" information pages for some content with additional features (e.g., educational films).	Uptake of doses 1 and 2	Completion of the intervention was associated with uptake.

Potts & Southard, 2019 [43]	United States<br><br>Primary care office located in rural, West-Central Indiana	N = 46<br>Boys and girls<br>9-17 years	Race = NR<br>SES = NR	N = 46 parents<br><br>Parent knowledge/attitudes	Parents watched a video featuring three mothers discussing the HPV vaccine. Two mothers had daughters and one mother had a son.	Uptake	Following the intervention, uptake increased from 35% to 41% for girls and from 12% to 21% for boys.
Pratt et al., 2021 [44]	United States<br><br>Urban primary care clinic that serves a large Somali immigrant population in Minnesota	N = 324<br>Boys and girls<br>11-20 years	Race = NR<br>SES = NR	N = 324 parents<br><br>Parent knowledge/attitudes	Somali and non-Somali parents participated in an intervention which modified usual care to require that parents and physicians discuss the vaccine. Additionally, parents received a tailored patient information tool featuring key messages including a faith-based message.	Uptake	There was a significant difference in uptake before and after the intervention.
Scarinci et al., 2020 [45]	United States<br><br>Predominantly Latinx communities in Alabama	N = 278<br>Girls<br>9-12 years	100% Hispanic<br>SES = NR	N = 278 parents<br>136 (intervention), 142 (control)<br><br>Parent knowledge/attitudes, health educator or patient/peer navigator	Parents received four informational group sessions and one individual session (home visit) with lay health educators. Parents in the intervention learned about HPV vaccination and parents in the control learned about healthy eating.	Initiation, continuation of series, completion of 3 doses	The intervention was associated with significantly higher initiation, uptake of dose 2, and completion than the control. Odds were 6x greater for the dose 1, 8x greater for dose 2, and 16x greater for dose 3.
Spleen et al., 2012 [46]	United States<br><br>Five-county service area of the ACTION Health Cancer Task Force, not-for-profit State Health Improvement Plan in Appalachian Pennsylvania affiliated with the Appalachia Community Cancer Network	N = NR<br>Boys and girls<br>11-17 years	NH White = 89%<br>Hispanic = 8%<br>American Indian/Alaska Native = 3%<br><br>Health insurance<br>Private = 57%<br>Public = 35%<br>Other = 3%<br>Uninsured = 5%	N = 38 parents<br><br>Parent knowledge/attitudes, health educator or patient/peer navigator	Parents received a 60-minute presentation about HPV and the vaccine delivered by professional health educators from the local community.	Initiation	Following the intervention, 44% initiated the vaccine series.
Staras et al., 2015 [89]	United States	N = 5663<br>Boys and girls	NH White = 47%<br>NH Black = 26%	N = 400 parents	The intervention included a postcard campaign encouraging	Initiation	The postcard condition was associated with greater odds of

	Four general pediatric and one family medicine clinic of an academic health center in North Central Florida	11-17 years	Hispanic = 14%<br>Other = 13%<br><br>Health insurance<br>Public = 81%	Parent self-efficacy, communication with provider	parents to discuss the vaccine with providers and an in-clinic health information technology program to gauge interest in the vaccine. Providers recommended the vaccine based on the information provided in the health information technology system. Adolescents were enrolled in the postcard campaign only, the in-clinic HIT system only, the postcard campaign and in-clinic HIT system, or usual care.		initiation for girls, but not boys.<br><br>The health information technology system was associated with greater odds for girls and boys.<br><br>The combined effect of the postcard and health information technology system was associated with the largest increase in initiation for girls and boys.
Woodall et al., 2021 [47]	United States<br><br>Nine pediatric clinics in New Mexico	N = 69<br>Girls<br>11-14 years	Hispanic = 39%<br>NH White = 38%<br>Other = 12%<br>American Indian/Alaska Native = 6%<br>Asian = 1%<br><br>Parent education<br>Less than high school diploma = 4%<br>High school diploma = 34%<br>College degree or higher = 61%	N = 82 parents<br><br>Parent knowledge/attitudes	Parents utilized a mobile application platform, *Vacteens/Vacunadolescente.or*g. The application had a video introduction by a well-known pediatrician in New Mexico, a Vaccine FAQ section, and five modules providing information about HPV and the vaccine ("Get Answers"), communicating about vaccination ("Let's Talk"), instructions for making an HPV vaccination appointment ("How-To"), interactive games for adolescents ("Teen Tools"), and sign up for a reminder system to facilitate completion of the vaccine series ("We're Ready").	Initiation, completion	Using the application was associated with a 20% increase in initiation and 37% increase in completion.
Wynn et al., 2021 [93]	United States<br><br>Community health clinics in New York City affiliated with the New York-Presbyterian Hospital Ambulatory Care Network and Columbia University	N = 956<br>Boys and girls<br>9-17 years	Race = NR<br><br>Health Insurance<br>Public = 95%	N = 956 parents<br>475 (intervention), 481 (control)<br><br>Reminder/recall	The intervention consisted of precision text message reminders. Parents first answered a short survey via text that assessed the family's stage of decision-making regarding continuation of the vaccine series 21 days following receipt of dose 1. Precision text messages were sent based on their stage of decision-making	Completion	There was no significant difference between the precision and standard groups in completion of the HPV series (73-76%). However, both groups were more likely to complete the series than historical controls.

					(i.e., precontemplation, contemplation, preparation). Parents in the control received standard text reminders notifying them when the next dose was due.		
**Results indicate intervention associated with mixed HPV vaccine outcomes**							
Maria et al., 2021 [66]	United States<br><br>Twenty-two after-school programs (e.g., Boys and Girls Clubs) and nineteen charter schools in medically under-served communities in Texas	N = 508<br>Boys and girls<br>11-18 years	Hispanic = 57% NH Black = 38%<br>NH White = 3%<br>Other = 2%<br><br>Health insurance<br>Public = 42%<br>Private = 33%<br>Uninsured = 16%	N = 519 parents<br><br>Parent knowledge/attitudes, communication with child	A sexual health intervention was adapted to promote HPV vaccination. The intervention had three components: a face-to-face session with parent; a take-home manual focused on consequences of sexual behavior; and, two follow-up calls. Parents in the control group had a face-to-face session about child exercise and nutrition, a brochure about healthy lifestyles, and two follow-up calls.	Initiation, completion	There were no significant differences in initiation and completion one-month post intervention.<br><br>Initiation was significantly higher in adolescents in the intervention (70%) than in the control group (61%), but completion was not.
Molokwu et al., 2019 [67]	United States<br><br>Community in El Paso County, Texas	N = 859<br>Boys and girls<br>9-17 years	Hispanic = 98%<br><br>Annual household income<br><$20,000 = 50%<br>$20–35,000 = 13%<br>>$35,000 = 8%<br>Don't know or refused = 30%	N = 859 parents<br><br>Parent knowledge/attitudes, health educator or patient/peer navigator, reminder/recall	Parents and adult participants received culturally-tailored education materials and met with patient navigators who provided community resources, scheduling, transportation assistance, and reminder messages.	Initiation, completion	Following the intervention, initiation was higher for adults than for children, and completion was higher for children than adults.
Parra-Medina et al., 2015 [68]	United States<br><br>Ten community resource centers of the Colonias Program in the Lower Rio Grande Valley of South Texas	N = 372<br>Girls<br>Age = 11-17	Race = NR<br><br>Health insurance<br>Insured = 10%	N = 372 parents<br>257 (intervention), 115 (control)<br><br>Parent knowledge/attitudes, peer navigators, communication with child	Mothers received a health education session from a trained *promotora* (Latino community health educator). The curriculum included information about HPV, the vaccine, and communication with their child about sex. Additionally, mothers were provided a community resource sheet that included information on local clinics offering free or low-cost HPV immunizations.	Initiation, completion of 3 doses	Initiation was not significantly different between groups, but adolescents in the intervention (72%) were more likely to complete the vaccine series than those in the control group (43%).

					Parents in the control group only received brochures.		
Staras et al., 2020 [90]	United States<br><br>Four general pediatric clinics and one family medicine clinic of an academic health center of North Central Florida	N = 1387<br>Girls<br>11-17 years	NH White = 49%<br>NH Black = 26%<br>Hispanic = 15%<br>Other = 13%<br><br>Health insurance<br>Public = 80%	N = 1387 parents<br><br>Parent self-efficacy, communication with provider	Parents in the intervention received two postcards by mail. The postcards aimed to encourage a conversation with their adolescent's health care provider about vaccination.	Uptake	There was no direct effect of the postcards on uptake. Postcards indirectly increased uptake by encouraging preventive health visits which were associated with uptake.
Underwood et al., 2019 [69]	United States<br><br>School district in one county in Georgia	N = 2135<br>Boys and girls<br>Middle and high school	Race = NR<br>SES = NR	N = 1358 parents<br><br>Parent knowledge/attitudes	Parents were enrolled in a parent-only educational intervention which included an educational brochure, a parent-adolescent version of the intervention which included the brochure and an interactive curriculum implemented by science teachers in classrooms of the adolescent, or the control group which did not receive any information.	Initiation	There was no significant difference in initiation between the parent-only condition and the control group.<br><br>Odds of initiation was significantly higher for the parent-adolescent condition than the control and the parent-only condition.
Winer et al., 2016 [70]	United States<br><br>The Hopi Reservation in northeastern Arizona	N = 88<br>Girls<br>9-12 years	100% Hopi Native American<br><br>Annual household income<br><$16,000 = 36-48%<br>$16,000–$34,999 = 34-38%<br>≥$35,000 = 14-30%	N = 88 parents<br><br>Parent knowledge/attitudes	Mother-daughter dyads attended two or three dinners that included an educational presentation comprised of a presentation and education brochure. The intervention group received information about HPV and the control group received information about juvenile diabetes.	Initiation, completion of 3 doses	Initiation was not significantly different between the intervention group and the control group.<br><br>For daughters who initiated the series, completion was not different between the intervention and control groups. For daughters who were unvaccinated at baseline, the intervention was associated with increased initiation and completion.
**Results indicate intervention not associated HPV vaccine outcomes**							
Baldwin et al., 2021 [87]	United States<br><br>Safety-net health care system in Texas	N = 161<br>Boys and girls<br>11-17 years	Hispanic = 77%<br>NH Black = 22%<br>NH White/other = 1%<br><br>Health insurance	N = 161 parents<br><br>Parent motivations	All parents watched a brief educational video and were assigned to one of four conditions:<br>Condition 1 - Select three of six topics to learn then verbalize	Initiation	No condition was significantly associated with vaccination status.

			Public = 68%<br>Private = 26%<br>Uninsured = 6%		three reasons for vaccination.<br>Condition 2 - Learn about three assigned topics then verbalize three reasons for vaccination.<br>Condition 3 - Select three of six topics to learn about then listen to three reasons for vaccination.<br>Condition 4 - Learn about three assigned topics then listen to three reasons for vaccination.		
Bastani et al., 2022 [75]	United States<br><br>Los Angeles County Department of Public Health Office of Women's Health health service telephone hotline	N = 238<br>Boys and girls<br>11-17 years	Latino = 69-72%<br>Asian = 20-23%<br>NH Black = 5-6%<br>NH White = 1-3%<br>Multiple = 0-1%<br><br>Annual income<br><$12,000 = 23-30%<br>$12,000–<$24,000 = 47-55%<br>$24,000–$36,000 = 10-21%<br>>$36,000 = 5-6%	N = 238 parents<br>138 (intervention), 100 (control)<br><br>Parent knowledge/attitudes	Parents using the Office of Women's Health hotline received a brief telephone education, a referral to a local HPV vaccine provider if needed, and a mailed brochure. Parents enrolled in the control group only received a mailed information sheet about HPV and the vaccine.	Initiation, completion (2 or 3 depending on age)	Initiation and completion were not significantly different between the intervention group and control group.
Chodick et al., 2021 [76]	Israel<br><br>Facebook	N = 21,979<br>Girls<br>14 years	Race = NR<br>SES = NR	N = 17,271 parents<br><br>Parent knowledge/ attitudes	Parents enrolled in the intervention received targeted Facebook news feed advertisements promoting HPV vaccination. The control group did not receive targeted advertisements.	Initiation	Initiation was not significantly different between the intervention group and the control group.
Coley et al., 2018 [77]	United States<br><br>New York State Department of Health excluding New York City	N = 162,452<br>Boys and girls<br>11-13 years	Race = NR<br>SES = NR	N = 162,452 parents<br>81,558 (intervention), 80,894 (control)<br><br>Parent knowledge/attitudes, communication with provider	Parents received a mailed letter encouraging communication with their child's provider about the vaccine and an information sheet.	Continuation of series, completion	The intervention showed little to no effect on continuation of the vaccine series or completion.
Froidevaux et al., 2023 [78]	Switzerland	N = 12,143<br>Boys and girls	Race = NR<br>SES = NR	N = 713 parents	The intervention updated the official Swiss brochure on HPV	Uptake	The updated brochure did not contribute to increased uptake.

	Schools in the Canton of Vaud	11-18 years		Parent knowledge/ attitudes	to include parents as a target audience and improve language on the benefits of the vaccine.		
Joseph et al., 2016 [79]	United States<br><br>Adolescent and pediatric primary care practices of a large urban hospital	N = 200<br>Girls<br>11-15 years	NH Black = 50%<br>Haitian = 50%<br><br>Annual household income<br><$20,000 = 45%<br>$20,000-35,000 = 29%<br>$35,000-60,000 = 19%<br>>$60,000 = 6%	N = 200 parents<br>98 (intervention), 102 (control)<br><br>Parent knowledge/attitudes, parent self-efficacy, communication with provider	Mothers received a brief negotiated interviewing intervention that assisted in evaluating their attitudes about the vaccine, addressed reasons for vaccination, and set goals to receive the vaccine. Mothers in the control group received an information sheet.	Initiation, continuation of series	Initiation and uptake of dose 2 or dose 3 were not significantly different between the intervention group and the control group.
Lee et al., 2018<br><br>[80]	United States<br><br>Community of Cambodian, largely Khmer population in Massachusetts.	N = 18<br>Girls<br>14-17 years	Race = NR<br>SES = NR	N = 18 parents<br>9 (intervention), 9 (control)<br><br>Parent knowledge/ attitudes	Mothers participated in a culturally-grounded intervention comprised of two story-based DVDs for the mother and daughter, and an additional "Learn More" video featuring a bilingual/bicultural Khmer American physician. Mothers in the control group only received an information sheet.	Initiation	There was no difference in vaccine initiation between the intervention and control group.
Pot et al., 2017<br><br>[81]	Netherlands<br><br>Online	N = NR<br>Girls<br>Born in 2002 or age 12	Race = NR<br>SES = NR	N = 8062 parents<br>2995 (intervention), 4067 (control)<br><br>Parent knowledge/attitudes, communication with child	Mothers interacted with a website that provided tailored feedback with two virtual assistants (a doctor and a mother). The website content included information about the HPV vaccine, pros and cons, how and where to get the HPV vaccine, and talking about the vaccine with their daughter or partner. Those in the control group did not have access to the website.	Uptake of doses 1 and 2	There was no significant difference in uptake between the intervention group and control group.
Richman et al., 2019 [82]	United States<br><br>Two rural community clinics in	N = 275<br>Boys and girls<br>9-17 years	NH Black = 60%<br>Hispanic = 28%<br><br>Health insurance	N = 257 parents<br><br>Parent knowledge/attitudes,	Following initiation of the vaccine series, parents in the intervention condition received seven electronic messages via	Uptake of doses 2 or 3	The intervention was not associated with uptake of dose 2 or 3.

	Pitt County and Greene County, North Carolina serving predominantly economically-disadvantaged, African American or Latino patients		100% uninsured or publicly insured	reminder/recall	email and/or text once per month across seven months including four health education messages and two appointment reminder messages. Parents in the control group received standard care.		
Rickert et al., 2015 [83]	United States<br><br>Five school-based health clinics operated by Teen Health Center, a nonprofit organization in collaboration with Department of Pediatrics at the University of Texas Medical Branch in Galveston County, Texas	N = 445<br>Boys and girls<br>11-15 years	Hispanic = 40%<br>NH White = 31%<br>NH Black = 28%<br>Other = 2%<br><br>Health insurance<br>Private = 46%<br>Public = 37%<br>Uninsured = 17%	N = 445 parents<br><br>Parent knowledge/attitudes	Parents received a health message intervention and were assigned to one of four conditions:<br>Condition 1 – Parents were asked two questions ("Do you want to protect your daughter/son from cervical cancer?" and "If there was a vaccine that could prevent cervical cancer/genital warts, would you have your daughter/son get it?"), and presented a one-sided message expressing benefits of the vaccine.<br>Condition 2 – Parent were asked the two questions and presented a two-sided message which stated that parents have concerns about the vaccine, but the vaccine is safe.<br>Condition 3 – Parents were only presented the one-sided message.<br>Condition 4- Parents were only presented the two-sided message.<br>All parents were mailed a vaccine information sheet and two reminders health messages consistent with their intervention condition.	Initiation, continuation of series, completion of 3 doses	The rhetorical question and message sidedness were not associated with initiation of the vaccine, dose 2, or completion.
Shegog et al., 2022 [84]	United States<br><br>Primary care network affiliated	N = 375<br>Boys and girls<br>11-17 years	White = 56%<br>Hispanic = 22%<br>Black = 13%<br>Asian = 6%	N = 375 parents<br>168 (intervention), 207 (control)	Parents in the intervention were given access to the *HPVcancerFree* application which included four sections	Initiation	Initiation was not significantly different between the intervention group and the control group.

	with Texas Children's Hospital and Baylor College of Medicine		Other = 3%<br><br>Health insurance<br>Private = 77%<br>Public = 21%<br>Other = 1%<br>Uninsured= 2%	Parent knowledge/attitude	providing HPV and vaccine facts ("HPV A-Z"), peer and provider testimonials addressing common barriers ("Bust a Myth"), resources to facilitate communication with providers ("Notes 4 Doc"), and a reminder system ("Get the Vax").		
Suzuki et al., 2022 [85]	Japan<br><br>Online	N = NR<br>Girls<br>11-18 years	Race = NR<br>SES = NR	N = 2175 parents<br><br>Parent knowledge/attitudes	All parents received an informational brochure about the HPV vaccine. Parents in the intervention additionally watched a short film featuring a cervical cancer survivor who had undergone a radical hysterectomy with bilateral salpingo-oophorectomy and pelvic lymphadenectomy talked about her experience.	Uptake	Uptake was not significantly different between the intervention group and the control group.

N = sample size; NR = not reported; NH = non-Hispanic; SES = socioeconomic status; HPV = human papillomavirus.
High school diploma may also include completion of GED equivalency diploma.
*Based on parent, adolescent, or practice data reported.

Supplementary Table S7. Summary of Provider-Level Studies (N = 17).

		Adolescent Targets		Intervention/Strategy			
Reference	Study Location/ Setting	Gender & Age	Race* & SES*	Modifiable Targets	Procedures/Description	HPV Vaccine Outcome	Findings
**Results indicate intervention associated improved HPV vaccine outcomes**							
Brewer et al., 2021 [155]	United States<br><br>Two large healthcare systems in a mid-western state	N = NR<br>Boys and girls<br>11-17 years	Race = NR<br>SES = NR	N = 234 providers (intervention)<br><br>Recommendation style	Providers participated in an online Announcement Approach Training that presented data on the effectiveness of presumptive announcements and timing of recommendations. The comparison group only sent reminders to patients needing dose 2 and dose 3.	Initiation	The intervention was significantly associated with increased initiation.
Brodie & McPeak, 2018 [126]	United States<br><br>Primary care practice located in an urban neighborhood of north Philadelphia	N = 6703<br>2854 (9-10 years),<br>3849 (11-13 years)<br>Gender = NR<br>9-13 years	Race = NR<br>SES = NR	N = 132 providers<br><br>Provider education, recommendation style	Providers received education on the HPV vaccine and providing a strong recommendation at every clinical encounter. Weekly educational emails were sent to providers. A wheel tool to help providers recommend the appropriate vaccine dose was distributed.	Initiation, missed opportunities	The educational intervention was identified as a special cause for an increase in initiation among adolescents 9-10 years from 56% to 84%, and a decrease in missed opportunities from 65% to 18%.
Fenton et al., 2021 [156]	United States<br><br>Pediatric and family medicine departments at three community health centers and one hospital based-practice	N = 82<br>Boys and girls<br>9-17 years	NH Black = 41%<br>NH White = 26%<br>Hispanic = 15%<br>Other = 18%<br><br>Parent education<br>Less than high school degree = 13%<br>High school diploma = 26%<br>Some college = 35%<br>Bachelor degree or higher = 26%	N = 82 visits<br><br>Recommendation style	The study procedures included recording clinical encounters where providers offered a recommendation. Visits were coded as indicated (e.g. "your child is due for the HPV vaccine") or elective (e.g. "are you interested in the HPV vaccine?").	Uptake	An indicated recommendation was associated with a 9x increase in odds of uptake compared to receiving an elective recommendation.
Real et al., 2022 [157]	United States<br><br>Cincinnati	N = 1099<br>403 (pre-intervention)	Race = NR<br>SES = NR	N = 134 providers<br>97 (intervention), 37	Providers participated in a virtual reality training comprised of simulated scenarios training	Initiation	The intervention was associated with a significant increase in initiation compared to the pre-intervention

	Children's Hospital Medical Center's Pediatric Primary Care Center (intervention site) and Hopple Street Neighborhood Health Center (comparison site), two academic pediatric primary care centers in Cincinnati, Ohio	696 (post-intervention)<br>Boys and girls<br>11-17 years		(control)<br><br>Provider communication	providers to counsel parents. Prior to the training, providers reviewed a mobile application on providing an effective recommendation. Each simulation provided training for a specific communication competency. Providers also learned motivational interviewing techniques to address parent hesitancy. Those in the control group did not complete the VR trainings.		period.
**Results indicate intervention associated with mixed HPV vaccine outcomes**							
Brewer et al., 2017 [137]	United States<br><br>Thirty clinics in North Carolina	N = 54,969<br>17,173 (11-12 years), 37,796 (13-17 years)<br>Boys and girls<br>11-17 years	Race = NR<br>SES = NR	N = Avg. of 5 providers per clinic<br><br>Provider education, provider communication, recommendation style	Providers received education and communication training that consisted of the latest research on HPV vaccination, skill-building to deliver effective HPV vaccine recommendations, a brief exercise to adapt the suggested material to their own personal style and language, role-play exercises, and open discussion about their clinical practice. Providers were assigned to three versions of the training: announcement training, conversation training, or wait-list control. The announcement training had three elements: mention child's age, announce that the child is due for 3 vaccines to be given today naming the HPV vaccine as second out of the three, strongly recommending same-day HPV vaccination if parents raised a concern. The conversation training utilized shared-decision making which included starting the conversation about 3 adolescent vaccines naming the HPV vaccine as second, normalizing the vaccine as routine, and inviting parent	Initiation, completion of 3 doses	Compared to the control group, initiation was significantly higher for adolescents 11-12 years seeing providers with announcement training, but the conversation training did not differ from the control in initiation for adolescents 11-12 years.<br><br>The intervention arms did not differ from the control group for completion among adolescents 11-12 or for any outcomes among adolescents 13-17.

					questions.		
Dawson et al., 2018 [138]	United States<br><br>Seven clinics in the Fort Hood Region of Texas	N = 67,000<br>Boys and girls<br>11-18 years	Race = NR<br>SES = NR	N = 200 providers<br><br>Provider education, provider communication, recommendation style	Providers participated in a standard "You are the Key to HPV Cancer Prevention" educational session. Sessions included role-playing on recommendations. Communication strategies learned were standardizing recommendations, focusing on cancer prevention, framing the vaccine in the context of other adolescent vaccines, and following published vaccine guidelines.	Uptake	The intervention was significantly associated with an increase in uptake 3-months post-interventions, but not 6-months post-intervention.
Dempsey et al., 2019 [158]	United States<br><br>Sixteen primary care practices in Denver, Colorado	N = 342<br>Boys and girls<br>11-18 years	NH White = 77%<br>Other = 14%<br>Asian = 5%<br>NH Black = 4%<br><br>Annual household income<br><$50,000 = 27%<br>$50,000-99,999 = 19%<br>≥$100,000 = 42%<br>Unknown = 12%	N = 188 providers<br><br>Provider communication, recommendation style	Providers were trained to give a presumptive recommendation and to use motivational interviewing techniques. Communication tools including a website, information sheet, set of HPV disease images, and vaccination decision aid were distributed.	Uptake	There was no difference in clinical records between the intervention group and control group. However, self-reported vaccine uptake was associated with a strong and presumptive recommendation.
Fiks et al., 2016 [139]	United States<br><br>Children's Hospital of Philadelphia primary care network in Pennsylvania and New Jersey	N = NR<br>Boys and girls<br>11-17 years	Race = NR<br>SES = NR	N = 27 providers<br><br>Provider education, provider communication	Providers participated in an online webinar that offered data on current vaccination rates in the primary care network, vaccine safety and efficacy, and strategies used. Providers were encouraged to use standard recommendation language, emphasize the importance of HPV vaccine in cancer prevention, and provide a recommendation during acute visits.	Initiation, continuation of series, completion	The intervention was associated with a significant increase in dose 1 at preventive visits and dose 1 and 2 at acute visits but not dose 2 or dose 3 at preventive visits or dose 3 at acute visits.
Fiks et al., 2023 [150]	United States<br><br>Pediatric primary	N = 65,123<br>34,188 (intervention),	NH White = 64%<br>Hispanic = 17%<br>NH Black = 10%	N = 234 providers<br><br>Provider	Providers participated in a communication training that also provided performance review and	Missed opportunities	Missed opportunities were decreased after the performance feedback period of the intervention, compared to the

	care practices in various states	30,935 (control)<br>Boys and girls<br>11-17 years	Asian or Pacific Islander = 4%<br>Multiple/other = 2%<br>American Indian/Alaska Native = 1%<br><br>SES = NR	communication, performance feedback	feedback. Providers in the control group continued with standard care practices.		communication training period. The cumulative intervention did not have a significant effect on missed opportunities.
Gilkey et al., 2014 [151]	United States<br><br>Ninety-one high-volume primary care clinics in North Carolina	N = 107,443<br>Boys and girls<br>11-18 years	Race = NR<br>SES = NR	N = NR<br><br>Performance feedback	Providers participated in an in-person or online webinar. Trainings included an evaluation of vaccine coverage, immunization best practices, how to maintain records, and how to decrease missed opportunities.	Initiation, completion	The in-person and webinar trainings did not differ from each other. The in-person and webinar trainings were associated with significant increases in initiation, but not in completion for adolescents 11-12 years. In-person training was significantly associated with only completion among adolescents 13-18 years.
Margolis et al., 2022 [159]	United States<br><br>Online probability-based, national panel maintained by a survey company	N = 1263<br>Boys and girls<br>9-17 years	NH White = 71%<br>Hispanic = 11%<br>NH Black = 10%<br>Other/multiple = 8%<br><br>Annual household income<br>≤$34,999 = 14%<br>$35,000-$74,999 = 24%<br>≥$75,000 = 61%	N = NR<br><br>Provider communication	The study examined provider response. An active response was defined as providers doing any of the following: give more information, offer to talk about the HPV vaccine again at a later visit, try to change parent's mind, or ask parent to sign a form confirming refusal to vaccinate. Follow up was indicated by any of the following: scheduling another visit to talk about the vaccine again, bringing the vaccine up again at the next check-up, or sending a reminder (phone, text, email or mail).	Uptake after declining	An active response was not associated with uptake of the vaccine after previously declining. Follow-up behaviors were associated with higher odds of uptake after decline.
Perkins et al., 2015 [140]	United States<br><br>One outpatient pediatric/adolescent department of an urban academic medical center and seven affiliated	N = 13,118<br>4093 (intervention), 9025 (control)<br>Boys and girls<br>11-21years	NH Black = 39-51%<br>Other = 23-38%<br>Hispanic = 9-14%<br>NH White = 5-10%<br>Asian = 1-10%<br><br>Health insurance	N = NR<br><br>Provider education, performance feedback	Providers received focused education on the morbidity and mortality from HPV, vaccine safety, vaccine efficacy, and motivational interviewing. Providers were also given performance feedback. Providers at control practices did not participate	Initiation, continuing the series	Initiation among girls was higher for providers in the intervention than in the control group during the intervention, but not post-intervention. Initiation among boys remained higher than the control during and after the intervention.

	federally qualified community health centers		Subsidized = 54-62%		in the intervention.		Getting the next needed dose was higher for girls and boys compared to the control group during and after the intervention.
Szilagyi et al., 2021 [141]	United States<br><br>Forty-eight pediatric practices across nineteen states	N = 104,438<br>50,569 (intervention),<br>53,869 (control)<br>Boys and girls<br>11-17 years	NH White = 64%<br>Hispanic = 17%<br>NH Black = 10%<br>Asian = 4%<br>American Indian/Alaska Native = 1%<br>Multiple/other= 1%<br><br>SES = NR	N = 234 providers<br><br>Provider education, provider communication, recommendation style	Providers reviewed three online modules covering HPV epidemiology, effective recommendation using a "same way same day approach," answering common questions, and addressing parental hesitancy. Module content was reinforced with weekly emails/texts. Providers at control practices continued standard care and participated in one session explaining the control arm of the study.	Initiation, continuation of series, missed opportunities	The intervention was associated with an increase in dose 1 and decrease in missed opportunities, but there was no significant difference in dose 2 or dose 3.
**Results indicate intervention not associated HPV vaccine outcomes**							
Austin & Morgan, 2019 [144]	United States<br><br>Three family practice offices within a large network of primary care providers in Northeast Florida	N = NR<br>Boys and girls<br>11-26 years	Race = NR<br>SES = NR	N = 12 providers<br><br>Provider education, performance feedback	Providers participated in an education session that utilized the standard "You Are the Key to HPV Cancer Prevention" presentation, received a packet and email links of resources from key organizations (CDC, KFF, AAP), and received performance feedback.	Initiation, completion	There was no significant effect of the intervention on initiation or completion rates.
Bradley-Ewing et al., 2022 [152]	United States<br><br>Four community-based practices affiliated with an integrated academic pediatric network in urban and suburban communities in the Midwest	N = 217<br>Boys and girls<br>9-17 years	White = 68%<br>NH Black = 28%<br>Other = 5%<br>Hispanic = 4%<br><br>Parent education<br>Some high school = 3%<br>High school diploma = 11%<br>Training or undergraduate degree = 66%<br>Graduate degree =	N = 16 providers<br><br>Provider communication, performance feedback	Providers were assigned to a single intervention comprised of performance feedback or a combined intervention which additionally included communication training and behavioral cues.	Vaccination rates	Uptake did not differ between intervention conditions.

			18%				
Gilkey et al., 2019 [145]	United States<br><br>Ambulatory care clinic in the Cook Children's Health Care System, a large, not-for-profit integrated delivery system based in Fort Worth, Texas	N = 22,983<br>Boys and girls<br>12-14 years	Race = NR<br>SES = NR	N = 77 providers<br><br>Provider education, provider communication, recommendation style	Providers participated in an in-clinic training session that provided information on HPV epidemiology, high-quality presumptive recommendations, and communication strategies such as emphasizing cancer prevention and providing follow-up counseling. Video vignettes were used to train providers on communication with hesitant parents.	Uptake	The intervention was not associated with uptake.
Irving et al., 2018 [146]	United States<br><br>Nine clinics in the Kaiser Permanente Northwest system in Oregon (intervention sites) and three Kaiser Permanente Northwest clinics in southwest Washington (control sites)	N = Avg. of 29,021 per quarter<br>Boys and girls<br>11-17 years	Race = NR<br>SES = NR	N = 150 providers<br><br>Provider education, provider communication, performance feedback	Providers received a standard education presentation using "You are the Key to Cancer Prevention" materials. The session also discussed communication strategies and provided clinic- and department-specific coverage and missed opportunity data. Both intervention and control clinics utilized common best practices prior to the intervention including standing orders, walk-in vaccine opportunities, electronic provider prompts, reminder letters, and performance feedback. During the intervention, providers at control clinics did not participate in the intervention.	Initiation, completion of 3 doses	The intervention did not differ significantly from the control group on initiation or completion.

N = sample size; NR = not reported; NH = non-Hispanic; SES = socioeconomic status; HPV = human papillomavirus.
High school diploma may also include completion of GED equivalency diploma.
*Based on parent, adolescent, or practice data reported.

Supplementary Table S8. Summary of Practice-Level Studies (N = 55).

		Adolescent Targets		Intervention/Strategy			
Reference	Study Location/ Setting	Gender & Age	Race* & SES*	Modifiable Targets	Procedures/Description	HPV Vaccine Outcome	Findings
**Results indicate intervention associated improved HPV vaccine outcomes**							
Abuelo et al., 2014 [23]	Peru<br><br>Urban (Iquitos) and rural communities along the Amazon rainforest	N = 318<br>Girls<br>10-13 years	Race = NR<br>SES = NR	N = 1 practice<br><br>Vaccination timing/schedule	Community health leaders developed a vaccine schedule that corresponded to the school calendar, administering two doses before the summer holiday and the final dose four months after the school year began.	Uptake	98% received dose 1, 91% received dose 2, and 72% received dose 3.
Chao et al., 2015 [94]	United States<br><br>Kaiser Permanente Southern California	N = 12,205<br>9760 (intervention),<br>2445 (control)<br>Girls<br>9-26 (57%-58% 9-17 years)	Hispanic = 50-51%<br>White = 22-24%<br>Black = 9-10%<br>Asian = 8-9%<br>Other = 3%<br><br>Health insurance<br>Public = 15%	N = NR<br><br>Reminder/recall	A reminder letter providing the HPV vaccine dose schedule, date of dose 1, and encouragement to complete the series was sent to participants. The control group received standard care.	Completion of 3 doses	Completion rates were significantly higher in the intervention group than in the control (56% vs. 47%). Among those who only had one dose, completion was significantly higher in the intervention than in the control (80% vs. 70%). These effects were stronger for girls ages 9-17 years than women 18-26 years.
Christensen et al., 2023 [165]	United States<br><br>Washington state Department of Health	N = NR<br>Boys and girls<br>9 years	Race = NR<br>SES = NR	N = 2 practices<br><br>Provider prompts, initiation age	Department of health updated provider prompts to recommend the HPV vaccine at age 9 instead of age 11 and from “optional” to “due now.”	Initiation	Initiation rate doubled following initiation at age 9. This was a 99% increase over what was expected.
Deshmukh et al., 2018 [166]	United States<br><br>Urban, hospital-based OB/GYN clinic	N = 3940<br>1176 (pre-intervention),<br>2764 (post-intervention)<br>Girls<br>11-26 years	Black = 44-47%<br>Hispanic = 36-38%<br>White = 8-12%<br>Other = 1%<br><br>Health insurance<br>Public = 80-85%	N = 1 practice<br><br>Standing orders, provider prompts	The practice implemented standing orders for HPV vaccines. Provider prompts were created to remind providers to screen for HPV vaccine eligibility.	Initiation, completion, missed opportunities	The intervention resulted in a significant increase in initiation (4x higher) and completion (3x higher) compared to the pre-intervention. Trend in the rate of missed opportunities deceased 4x faster after the intervention than before.
Desiante et al., 2017 [160]	Italy<br><br>Clinic- and school-based HPV vaccination centers served by Local Health Unit of	N = 5720<br>Boys and girls<br>12 years (cohort of adolescents born in 2003)	Race = NR<br>SES = NR	N = 24 centers<br>10 clinic-based vaccination centers,<br>14 school-based vaccination centers<br><br>Vaccine access	Vaccination centers were randomly assigned to the traditional condition where families were given an invitation letters to go to the vaccination clinic for the vaccine. Other centers involved secondary schools where families	Non-completion after dose 1	School vaccination resulted in vaccination among 85% and 60% of females and males, respectively. Compared to 9% of non-completion in school-based sites, 15% in outpatient clinics did not complete the vaccine series after receiving dose 1.

	Taranto				were provided counseling and promotional meetings and the vaccine was adaminsted during school time.		
Ferreira et al., 2022 [29]	Brazil<br><br>Elementary schools in two municipalities in the state of Ceará, Northeast Brazil	N = 210<br>Girls<br>9-14 years	Race = NR<br>SES = NR	N = 6 schools<br><br>Reminder/recall	The intervention was a message card promoting adherence to the HPV vaccine series. The message card was given to students twice a week during school for two months. The control group only received vaccine counseling by nurses at health care units or by the school.	Uptake	The intervention resulted in 53% uptake.
Fujiwara et al., 2013 [95]	Japan<br><br>Tochigi Prefecture	The local governments that conducted school-based vaccination programs targeted 810 students with a 100% subsidy.<br>Girls<br>13-15 years	Race = NR<br>SES = NR	N = 22 local governments<br><br>Program subsidies, reminder/recall	Some school-based and community-based programs provided subsidies for vaccination to offer no-cost HPV vaccine. Practices mailed a recall notice and free ticket once. An additional recall was sent if there was no response. Notices included information on immunization stage, available dates, and immunization sites.	Uptake	Coverage was ≥90% for all doses in school-based programs (dose 1: 97%, dose 2: 96%, dose 3: 91%), but varied in subsidy programs (dose 1: 46-95%, dose 2: 41-94%, dose 3: 3-90%). The most effective combination of strategies in the subsidy condition was a free ticket for vaccine and direct mail of notice and recall (dose 1: 95%, dose 2: 94%, dose 3: 90%). No subsidy was associated with lowest coverage (0-1%).
Gilkey et al., 2023 [148]	United States<br><br>Departments of Health in Illinois, Michigan, and Washington State	N = 312,227<br>Boys and girls<br>11-17 years	Race = NR<br>SES = NR	N = 224 practices<br><br>Performance feedback, provider communication/ recommendation, standing orders	Practices received performance feedback from a consultation coach and implemented a brief action plan of evidence-based strategies including increasing the frequency and quality of provider recommendations and establishing standing orders. Control practiced did not receive the intervention.	Initiation	Initiation among ages 11-12 was higher in the in-person and virtual coaching arms compared to the control. Initiation among ages 13-17 was higher in the virtual arm compared to the control.
Glenn et al., 2023<br><br>2023 [96]	United States<br><br>A large, multi-site federally qualified health center in Los Angeles County,	N = 877<br>417 (intervention), 460 (control)<br>Boys and girls<br>12 years	Latino = 85-86%<br>White = 7-10%<br>Other = 5%<br><br>Health insurance<br>Public = 52-74%	N = 6 clinical sites of one health center<br><br>Reminder/recall	Practices implemented one of three reminder systems: mailed letter, automated phone call (robocall) or text message. Contact occurred once at baseline and one month after baseline. Control clinics	Continuation of series	Compared to usual care (12% continued series), 23% in any reminder system received the next dose needed.

	California		Uninsured = 2-3%		implemented no reminder strategies.		
Goleman et al., 2018 [181]	United States<br><br>Nationwide Children's Hospital, an academic children's hospital in Columbus, Ohio	Boys and girls<br>9 & 10 years<br>3741 (pre-intervention), 4047 (post-intervention)<br>11 & 12 years<br>3635 (pre-intervention),<br>4130 (post-intervention)	9 & 10 years<br>Black = 53-54%<br>White = 19-23%<br>Latino = 12-17%<br>Other = 7-8%<br>Asian = 3-4%<br>Not provided for 11 & 12 year olds<br><br>Health insurance<br>9 & 10 years olds<br>Private = 10-14%<br>Public = 79-84%<br>Self = 6-8%<br>Not provided for 11 & 12 year olds	N = 14 practices<br><br>Initiation age, vaccine messaging	Provider prompts were updated to recommend vaccination at age 9 and providers were encouraged to focus on cancer prevention benefits of the vaccine.	Initiation	Initiation increased from 36% (6 months following the intervention) to 61% (18 months following the intervention) among ages 9-10. Initiation increased from 70-85% for ages 11-12 years.
Howhü & Bro, 2012 [176]	Denmark<br><br>General practices in the Region Central Jutlan	N = NR<br>Girls<br>14-16 years	Race = NR<br>SES = NR	N = 100 practices<br><br>Patient invitation	Providers contacted unvaccinated girls with a standard letter or letter and phone call.	Initiation	Girls contacted by their practice were 15% more likely to initiate the vaccine series than girls not contacted.
Kempe et al., 2016 [97]	United States<br><br>Kaiser Permanente Colorado	N = 929<br>374 (intervention), 555 (control)<br>Boys and girls<br>11-17 years	White = 48-54%<br>Hispanic = 22-26%<br>Other = 13-18%<br>Black = 8-14%<br><br>SES = NR	N = 7 practices<br>4 (intervention), 3 (control)<br><br>Reminder/recall	Recall messages were sent via text, email, or automated phone message for adolescents who missed an HPV dose. Parents/adolescents chose up to two methods of contact. Practices in the control group provided usual care and did not implement reminders or recalls.	Completion, on-time continuation of series	Recommended time intervals for doses 1, 2, and 3 were more likely to occur at intervention practices. A combination of phone and email recalls were most effective. Rates of completion were higher for younger adolescents.
Kharbanda et al., 2011 [98]	United States<br><br>Five hospital-affiliated academic clinical sites and four private pediatric clinical sites located in New York City	N = 434<br>124 (intervention), 308 (control)<br>Girls<br>9-20 years	Race = NR<br><br>Health insurance<br>Public = 68-90%<br>Private = 10-24%<br>Uninsured = 0-8%	N = 9 practices<br><br>Vaccine access, Reminder/recall	Girls were able to receive dose 2 and 3 at vaccine-only clinic visits. Reminder messages for subsequent doses were sent up to three times a week at least three weeks prior to the dose due date. Two control groups: an opt-out group comprised of those who did not	Continuation of series (on-time, within 4 months)	Those receiving reminder messages were significantly more likely to receive the next dose on time than those who opted-out (52% vs. 35%) and historical controls (38%). The intervention group was also significantly more likely to receive the next dose within 4 months than

					participate in the intervention, and a historical control group comprised of girls who received dose 1 and dose 2 during a 5-month control period prior to the intervention.		those who opted-out (65% vs. 51%) and historical controls (53%).
Mantzari et al., 2015 [99]	England<br><br>Community clinics within the Birmingham East and North and Heart of Birmingham Primary Care Trusts	N = 1000<br>500 (intervention), 500 (control)<br>Girls<br>16-18 years	Race = NR<br>SES = NR	N = 3 practices<br><br>Reminder/recall, Patient incentives	Reminder letters were sent out to unvaccinated patients ahead of their dose 1 appointment. The letter included an informational brochure. Text message reminders were sent for dose 2 and 3. Patients were offered a financial incentive for each dose for a total of £45 (€52; $65) for completing the vaccine series. Those in the control group were sent a standard invitation letters, but were not given financial incentives.	Initiation, completion	Financial incentives were associated with 10% increase in initiation, and a combination of financial incentive and text message was also associated with a 10% increase in completion.
Moss et al., 2012 [100]	United States<br><br>Federally qualified health centers in North Carolina	N = 15,660<br>7827 (pre-intervention), 7833 (post-intervention)<br>Boys and girls<br>12-17 years	Race = NR<br>SES = NR	N = 17 practices<br><br>Provider prompts, standing orders, immunization registry, reminder/recall, performance feedback	During the performance feedback intervention, clinics implemented evidence-based strategies such as reviewing and flagging charts to reduce missed opportunities, standing orders, establishing use of the state immunization registry to create reminder letters, and use of automated phone calls for reminders.	Initiation, dose 2, completion	There was significant increases in initiation (52% to 54%), receipt of dose 2 (35% to 36%), and completion (21% to 22%) from baseline to follow-up.
Muhamad et al., 2018 [26]	Malaysia<br><br>School Health Service Program	N = 242,638<br>Girls<br>12-13 years or grade 7	Race = NR<br>SES = NR	N = 450 schools<br><br>Vaccine access, media/advertisement campaign	A national school-based vaccination program was implemented in public and private schools. Community nurses administered free vaccines at three scheduled school visits occurring before the end of the school year. A campaign including electronic, print in newspapers and magazine, and social media content was also conducted.	Initiation, dose 2, completion	Uptake of dose 1, 2, and 3 reached to 99% or higher.

Perkins et al., 2021 [101]	United States<br><br>Ten urban and one rural federally qualified health centers in Alabama, Louisiana, Oklahoma, and Texas recruited by Vaccinate Adolescents Against Cancers	N = 3804 to 58,873 per clinic<br>Boys and girls<br>13 years	Racial ethnic minority = 49-96%<br><br>≤ 100% of Poverty level = 54-87%	N = 45 practices<br><br>Reminder/recall, provider prompts, standing orders	Several evidence-based strategies were implemented including reminder/recalls through telephone, letter, postcard, and/or text message; provider prompts delivered through daily huddles, chart notes, and electronic health record system; and standing orders.	Initiation, completion	Initiation increased from 47% to 71%, and completion increased from 25% to 46%. Results indicated large effects, suggesting a 90% greater change of vaccination during the intervention.
Poscia et al., 2021 [167]	Italy<br><br>Three Italian Regions (Lazio, Basilicata and Sicily)	N = 755<br>265 (intervention), 490 (control)<br>Boys and girls<br>Avg. of 12.3 years	Race = NR<br>SES = NR	N = 6 schools<br>1 intervention and 1 control school per region<br><br>Vaccine access	Local health authorities scheduled at least one day to administer vaccinations for students. Matched control schools did not receive the intervention.	Uptake	The intervention was associated with 11% increase in uptake.
Rodriguez et al., 2022 [102]	United States<br><br>Rio Grande City Independent School District and Roma Independent School District in Starr County; Zapata County Independent School District; and PSJA (Pharr-San Juan-Alamo) Independent School District in Hidalgo County in Texas	N = 1,766<br>Boys and girls<br>9-14 years, >14 years	Race = NR<br>SES = NR	N = 11 schools<br><br>Vaccine access, reminder/recall	Middle schools implemented vaccine programs. Vaccines were administered at back-to-school events, progress report nights, and preview events. Additionally, a maximum of five reminder letters, text messages, and phone calls were sent for remaining doses. Catch-up vaccines were scheduled with local clinics, if needed. Extra school-based events were planned for missed doses.	Up-to-date status	The overall percent of up-to-date adolescents was 60%. A larger proportion of those who initiated the vaccine at ages 9, 10, or 11 (74%) were up-to-date compared to those who initiated at age 12 or older (45%). Up-to-date status was highest among middle school students who initiated the series at age 11.
Ruffin et al., 2015 [168]	United States<br><br>Academic, community-based family medicine practices in the Midwest	N = 15,021<br>5994 (intervention), 9027 (control)<br>Girls<br>9-18 years	Intervention only<br>White = 16-67%<br>Black = 18-22%<br>Other = 13-18%<br><br>SES = NR	N = 9 practices<br><br>Provider prompts, patient prompts	Provider prompts were implemented. The alert indicated whether the HPV vaccine was a needed service and specified the dose required at the visit. Providers were required to respond "done," "ordered," "declined," "not eligible," "discussed," or "not	Initiation, completion of 3 doses, time to completion, time interval between doses	The prompt intervention was associated with significantly greater odds of initiation, quicker completion of series, and less time between doses 1, 2, and 3 compared to the control.

					addressed" after the visit. Parents/patients received a note outlining services that will be recommended during the visit. Control practices had a common electronic health record system, but did not have any electronic prompting or alert system for vaccines.		
Rutten et al., 2024 [103]	United States<br><br>Mayo Clinic primary care practices in Southeastern Minnesota	N = 9242<br>Boys and girls<br>11-12 years	White = 73%<br>Other = 12%<br>Black = 9%<br>Asian = 5%<br>Hispanic = 2%<br><br>SES = NR	N = 6 practices<br><br>Reminder/recall, immunization registry, standing orders, patient comfort, performance feedback	The stepped-wedge intervention included usual care consisting of the electronic immunization registry, standing orders allowing nurses to administer vaccines, pain preventive measures, and point-of-care prompts for vaccines. Reminder/recall procedures were implemented with presumptive language mailed letters describing standard care for the HPV vaccine (nurse visits, pain measures, etc.).	Uptake	The reminder/recall intervention was associated with greater odds of uptake, but the audit/performance feedback intervention was not. A combination of the two interventions was associated with double odds of uptake compared to usual care.
Tull et al., 2019 [104]	Australia<br><br>Seventy-seven local government immunization providers in Victoria	N = 4386<br>1442 (motivational arm), 1418 (self-regulatory arm), 1526 (control)<br>Boys and girls<br>Avg. of 13.2 years	Race = NR<br>SES = NR	N = 31 schools<br><br>Reminder/recall	Recall messages were sent to parents before a school vaccination visit. Three conditions were tested: Motivational text messages discussed HPV risk to child; self-regulatory messages focused on parent efficacy to get the vaccine dose on time; and controls received no text message.	Uptake	There was a significant effect of both text message reminders on uptake compared to the control.
Watson-Jones et al., 2012 [30]	Tanzania<br><br>Sixty urban government, Sixty rural government, and fourteen private schools in northwest Mwanza region	N = 5532<br>3352 (class-based model), 2180 (age-based model)<br>Girls<br>Year 6 (median age = 13) or 12 years (born in 1998)	Race = NR<br>SES = NR	N = 131 schools<br>Conditions:<br>(age-based = 64; class-based = 67)<br><br>Initiation age	Schools implemented one of two delivery strategies. The age-based strategy provided the vaccine to girls born in 1998 (same age) while the class-based strategy provided the vaccine to girls in Year 6 (same school grade). The vaccine was given in four rounds over 12 months.	Uptake	There was no difference in either strategies for dose 1 (86% vs 82%). However, the class-based strategy was associated with greater uptake of dose 2 (84% vs 78%) and dose 3 (79% vs 72%) than the age-based strategy.
Whelan et al., 2014	Canada	N = 3291	Race = NR	N = 48 schools	Schools implemented vaccine	Initiation,	74% of students completed the series.

[105]	The Capital Health District's school-based immunization program in Nova Scotia	Girls<br>Avg. of 13.7 years	SES = NR	Reminder/recall, consent procedures, vaccine access	programs. Teachers and other school personnel were provided HPV education. Nurses were assigned to schools. Consent forms were returned to teachers. Reminder/recall were used for return of consent forms and for missed clinic visits.	completion of 3 doses	Reminder calls to return the consent forms were associated with 2x greater odds of initiation. Having a nurse at the school was associated with 1.6x greater odds of completion and returning consent forms to teachers was associated with 3x greater odds of completion which accounted for 99% of variance in completion rate.
Wilkinson et al., 2014 [169]	United States<br><br>Urban, safety-net health clinics in the Eskenazi Health system in Indianapolis, Indiana	N = 1285<br>634 (intervention), 651 (control)<br>Boys and girls<br>11-17 years	Black = 59%<br>Hispanic = 22%<br><br>SES = NR	N = 5 practices<br><br>Provider prompts	Automated clinical decision support system prompts were set to identify adolescents needing dose 2 and dose 3. In the control, nurses manually identified adolescents needing the vaccine.	Uptake of dose 2, dose 3	Following the intervention, 54% of patient encounters received dose 2 and 46% received dose 3. There were no differences in automated and nurse-provided prompts.
Zimet et al., 2018 [170]	United States<br><br>Pediatric primary care clinics in urban Indianapolis that are part of Eskenazi Health, a large safety net health system serving Marion County, Indiana.	N = 648<br>Boys and girls<br>11-13 years	NH Black = 38-70%<br>Other = 17-21%<br>NH White = 7-14%<br>Hispanic = 6-30%<br><br>SES = NR	N = 5 pediatric primary care clinics with 29 providers total (10 usual practice, 8 simple condition, 11, elaborate condition)<br><br>Provider prompts	Providers were randomized to 1 of 3 conditions. Condition 1: Usual practice; Condition 2: The simple prompt condition utilizing computer-generated messages reminding providers of vaccine eligibility for three adolescent vaccines including the HPV vaccine; Condition 3: An elaborated prompt that additionally included a suggested script for recommending the adolescent vaccines.	Uptake	The elaborated prompt condition was associated with significantly higher uptake than the usual practice group. The simple prompt and elaborated prompt were not significantly different in uptake.
**Results indicate intervention associated with mixed HPV vaccine outcomes**							
Bae et al., 2017 [171]	United States<br><br>National Ambulatory Medical Care Survey (NAMCS)	Three adolescent target groups: (1) 3388 visits with adolescents ages 11-12 years, (2) 14,354 visits with adolescents ages 11-18 years, (3) 25,573 visits with females ages 11-26 years and males ages 11-21	Race = NR<br>SES = NR	N = NR<br><br>Provider prompts	This study examined whether health care practices used an electronic health record system to generate provider prompts and whether use was "routine" or "nonroutine."	Uptake	Practices routinely using clinical reminders were more likely to order HPV vaccines than non-use and non-routine use practices. Clinical reminders were associated with higher vaccination rates for female patients ages 11 to 26 years and male patients ages 11 to 21 years (group 3). However, associations were not significant for patients 11-12 years and 11-18 years overall (groups 1 and

		years					2).
Bar-Shain et al., 2015 [112]	United States<br><br>Large academic tertiary public health care system in Ohio	85% (2897) of 3393 adolescents overdue for the HPV vaccine<br>Boys and girls<br>11-18 years	Black = 38%<br>White = 23%<br>Hispanic = 19%<br>Other = 4%<br><br>Health insurance<br>Public = 79%<br>Other = 21%	N = NR<br><br>Provider prompts, reminder/recall	Practices' electronic health record were set to identify all adolescents due for HPV vaccines, alert providers that vaccination was needed during face-to-face encounters, and provide data for messaging parents. A stepwise messaging cascade sent parents emails, text messages, or a postcard reminders for vaccine scheduling. A well-child visit, nurse immunization visit, or non-well-child visit was scheduled.	Uptake	Those needing dose 3 were more likely to uptake than those needing dose 1 and 2. Those needing more than one vaccine were 1.3x more likely to be vaccinated. Receiving one message was associated with greater likelihood of uptake than two or three messages, of which text messages and postcards were more effective than phone calls.
Fisher et al., 2022 [178]	England<br><br>Local health authorities in the Southwest of England	N = 13,290<br>6343 (pre-intervention),<br>6947 (post-intervention)<br>Girls<br>"School-aged"	NH White = 68%<br>Asian = 5%<br>Multiple/other = 3%<br>NH Black = <1%<br><br>Level of deprivation index<br>Least, 1 = 19.9%<br>Level 2 = 19.8%<br>Level 3 = 20.1%<br>Level 4 = 20.0%<br>Most, 5 = 20.2%	N = 2 local health authorities<br><br>Consent procedures	New consent procedures were implemented where parents were contacted by phone if written consent was not obtained. If parents could not be contacted, the immunization team allowed participants deemed “Gillick-competent" to self-consent.	Uptake	Overall, uptake increased from 88% in the pre-intervention period to 89% in the post-intervention period. A significant intervention effect was only observed for Local Authority One where uptake increased form 76% to 83%.
Fuller & Papachrisanthou, 2017 [177]	United States<br><br>Midwest inner city clinic	N = NR<br>Gender = NR<br>≥ 13 years	Race = NR<br>SES = NR	N = 1 practice<br><br>Patient navigators	A patient educator program was implemented. The curriculum focused on HPV epidemiology, vaccine availability, and dissemination practices.	Uptake	The intervention was not associated with a significant increase in uptake, although the increase (N = 32) was clinically significant.
Gurfinkel et al., 2021 [111]	United States<br><br>Pediatric, family medicine, or community health centers in eight urban counties along the Front Range in Colorado, and in all	N = 32,283 (Colorado),<br>37,003 (New York)<br>Boys and girls<br>11-14 years	Race = NR<br>SES = NR	N = 224 practices (Colorado = 88, NY = 136)<br><br>Reminder/recall	A centralized reminder/recall system was implemented. In Colorado, reminder/recall was sent by autodialing and mail. In New York, they were sent by autodial and text message. The control group was usual care (no implemented system).	Initiation, completion	In Colorado, the intervention was not associated with improved initiation but the rate of completion was higher for both mailed and autodialer methods compared to the control. In New York, the intervention was not associated with improved initiation or completion.

	57 upstate urban, upstate rural, and downstate counties in New York (excluding New York City)						
Hanley et al., 2023 [113]	United States<br><br>Primary care clinics of one health care organization in Houston, Texas	N = 7408<br>3705 (intervention), 3703 (control)<br>Boys and girls<br>9-25 years	Black = 26%<br>Other = 25-26%<br>Hispanic = 24-25%<br>White = 23-25%<br><br>Health insurance<br>Private = 54-55%<br>Public = 36%<br>Uninsured = 8-9%	N = 13 practices<br><br>Reminder/recall	Electronic reminders were sent to parent or patient based on age via text message, patient portal (MyChart) or email depending on parent/patient preference. The control group provided usual care.	Continuation of series, initiation, completion	The intervention was associated with increased odds of getting an additional dose, but not significantly associated with initiation or completion.
Henrikson et al., 2018 [118]	United States<br><br>Group Health Cooperative (now Kaiser Permanente Washington)	N = 1624<br>1224 (intervention), 400 (control)<br>Boys and girls<br>10-12 years	White = 63-65%<br>Asian = 16%<br>Black = 6-7%<br>Hispanic = 2%<br>Native = 2%<br><br>SES = NR	N = 7 practices<br><br>Reminder/recall	Parents received a reminder letter signed by the Group Health lead immunization physician and lead immunization nurse providing a recommendation for the vaccine, information about the dose schedule, and where to get the vaccine within Group Health. Parents also received an automated interactive voice recognition reminder call for dose 1, 2, and 3. Control practices provided usual care that did not include a reminder letter or call.	Completion, time to initiation	There was no significant difference in the intervention and usual care groups in completing the vaccine series within 210 days of dose 1, but the intervention reported overall higher rates of completion. Reminders for dose 2 or 3 showed no effect compared to receiving a reminder for dose 1 only.
Ladner et al., 2012 [27]	Bhutan, Bolivia, Cambodia, Cameroon, Haiti, Lesotho, and Nepal<br><br>Eight programs implemented in urban, rural, and mixed areas across seven countries	N = 87,580<br>Girls<br>9-13, 10-18 years	Race = NR<br>SES = NR	N = 8 project countries totaling 423 vaccination sites<br><br>Vaccine access	Intervention projects administering free vaccines were provided in a school-based model, a health facility-based model, or a mixed model (i.e., school and health facility).	Completion, uptake	Completion was higher in the school-based (93%) and mixed (94%) models than the health facility model (77%). Between dose 1 and dose 3, the mixed model was more effective (97%) than the school (89%) and health models (80%).

Mackey et al., 2019 [172]	United States<br><br>Center for Family Medicine, a family practice ambulatory clinic in Sioux Falls, South Dakota	N = 247<br>Boys and girls<br>11-12 years	Black = 41%<br>Caucasian = 37%<br>Other = 8%<br>American Indian = 7%<br>Asian = 4%<br>Hispanic = 3%<br><br>Health insurance<br>Public = 50%<br>AveraHealthPlan = 25%<br>Private = 14%<br>Other = 11%	N = 1 practice<br><br>Patient invitation, clinic materials, provider prompts	Letters that provided basic information about the vaccine and contract information for the clinic were mailed. At the clinic, fliers and posters were placed in several areas including patient waiting rooms, restrooms, check-in area, and exam rooms. Provider prompts were implemented through the clinic electronic health record system and in weekly meetings. In the electronic health system, HPV vaccination status was added for all patient under 12 years of age.	Initiation, completion of 2 doses	Following the mailing intervention, initiation rose from 47% to 60% and completion rose from 21 to 26%. Following the second part of the intervention, initiation increased to 69% and completion to 28%. Initiation was significantly higher than baseline, but completion was not.
Mugisha et al., 2015 [25]	Uganda<br><br>The Uganda Ministry of Health, in collaboration with PATH, an international non-profit organization	N = 5722<br>3459 (Ibanda), 2263 (Nakasongola)<br>Girls<br>Age 10 or primary grade 5	Race = NR<br>SES = NR	N = 31 health facilities, 52 schools<br><br>Initiation age	Districts implemented one of two delivery strategies. The age-based strategy provided the vaccine to girls age 10 years while the class-based strategy provided the vaccine to girls in P.5 classes (same school grade). Both strategies provided referrals to neighboring health units or through regular routine outreach for girls age 10. Vaccinations were administered over a few days each for dose 1, 2, and 3 in a six-month period. Two rounds of vaccination were conducted.	Initiation, completion	Researchers found that the grade-based model was more effective in terms of initiation and completion rates, and determining the eligibility of the girls was easier in the grade-based. The age-based model likely reflected over-estimation of intervention effect because girls outside of age range were vaccinated as well.
O'Leary & Frost, 2023 [173]	United States<br><br>Denver Health and Hospital Authority in Denver, Colorado consisting of federally qualified health centers	N = 25,888<br>12,433 (pre-intervention),<br>13,455 (post-intervention)<br>Boys and girls<br>9-12 years	White = 64-66%<br>Black = 15%<br>Asian = 4%<br>Multiple = 1%<br>American Indian/Alaska Native = <1%<br>Native Hawaiian/other Pacific Islander = <1%<br><br>Health insurance	N = 28 (all primary care specialties that see children within Denver Health and Hospital Authority)<br><br>Standing orders, initiation age, provider prompts, vaccine access	Standing orders were implemented and the vaccine was offered at any outpatient and inpatient in-person visits. The recommended age for initiation was lowered to 9 years for all patients. Electronic record alerts (Best Practice Advisories) were set to alert providers to patients needing the vaccine.	Initiation, dose 2, completion	Initiation among ages 9-12 years increased from 42% to 54% with increases among those 9-10 years (<1% to 24%) and those ages 11-12 (66% to 74%). At least one dose by 13 years increased from 30 to 43%, and receipt of dose 2 increased from 19% to 43%. Completion for those 9-12 years remained stable.

			Private = 9%<br>Public = 84-86%<br>Uninsured = 6-7%				
Oliver et al., 2021 [161]	United States<br><br>Practices recruited by four American Academy of Pediatrics chapters in Arizona, New Jersey, and Oregon	N = NR<br>Boys and girls<br>11-14 years	Race = NR<br>SES = NR	N = 9 practices<br><br>Vaccine access, vaccine messaging, vaccine procedures, provider prompts, initiation age, standing orders	Several evidence-based strategies were implemented including follow-up vaccine appointments, administering the vaccine at sick visits, staff training on improving vaccine communication/provider recommendation, bundling vaccines, flagging charts that need the vaccine, lowering initiation age to 9 or 10 years, and implementing standing orders.	Initiation, Completion	Initiation increased from 54% to 69% and completion remained stable (88 to 86%). However, aggregate data over 8 months demonstrated no significant change.
Rand et al., 2015 [114]	United States<br><br>Monroe Plan for Medical Care, a large not-for-profit managed care organization in upstate New York	N = 3,812<br>1893 (intervention), 1919 (control)<br>Boys and girls<br>11-16 years	Race = NR<br><br>Health insurance<br>Public = 100%	N = 39 practices<br><br>Reminder/recall	A maximum of four reminder text messages were sent to parents for dose 1, 2, or 3. Parents in the control group received messages about a general adolescent health topic (e.g., healthy eating) when parents in the intervention were sent reminder messages.	Uptake	The intervention was associated with a significant increase in dose 1 uptake (16% intervention arm vs. 13% control arm), but not in dose 2 or 3.
Rand et al., 2017 [115]	United States<br><br>Urban pediatric, medicine pediatric, and family medicine primary care clinics in Rochester, New York	N = 749<br>178 (phone arm, control = 180), 191 (text message arm, control = 200)<br>Boys and girls<br>11-17 years	Black = 61-71%<br>Other = 16-18%<br>White = 13-21%<br>Hispanic = 11-13%<br><br>Health insurance<br>Phone group<br>Public = 79-82%<br>Private = 14%<br>Uninsured = 5-7%	N = 3 practices<br><br>Reminder/recall	Telephone or text message reminders were implemented. A maximum of three reminders per dose were sent one week apart. The control group was standard care.	Uptake, completion of 3 doses, time interval between doses	There were no significant differences in uptake of dose 1, 2, or 3. The phone intervention was associated with significantly quicker uptake of dose 2 and dose 3 for those who enrolled at dose 1. The text intervention was associated with significantly greater series completion than the control (49% vs 31%) and significantly quicker uptake of dose 2, dose 3, and completing the series.
Riviere et al., 2021 [28]	Haiti<br><br>The GHESKIO (Haitian Study Group on Kaposi's Sarcoma and Opportunistic	N = 2445<br>1,698 (non-school cohort), 747 (school cohort)<br>Girls<br>9-14 years	Race = NR<br>SES = NR	N = 14 vaccination sites<br>1 clinic, 13 school-based sites<br><br>Vaccine access	Non-school and school-based programs were implemented. Girls were recruited from GHESKIO adolescent and pediatric clinics. For non-school programs, girls were vaccinated at the GHESKIO vaccine clinic. For school-based	Uptake, completion of 3 doses	More participants in the school program consented to vaccination than in the non-school program (92% vs. 77%). Overall, there was no significant difference in uptake between groups. However, of girls previously seen at the GHESKIO

	Infections) clinic or schools near the clinic				programs, schools near the GHESKIO clinic were identified. Girls with guardian consent were vaccinated at school.		clinic, odds of receiving dose 2 were double that of girls who had never been to the GHESKIO clinic and completion was 4x greater in school than girls vaccinated in a clinic setting.
Szilagyi et al., 2020 [116]	United States<br><br>Pediatric, family medicine, or community practices in Colorado and New York	N = 30,616 (New York), 31,502 (Colorado)<br>Boys and girls<br>11-18 years	Race = NR<br>SES = NR	N = 213<br>(New York = 123, Colorado = 90<br><br>Reminder/recall	Practices sent one, two, or three reminder/recalls per dose. Reminder/recalls were autodialer phone calls.	Initiation, completion	In New York, the intervention was not associated with any differences in initiation or completion. In Colorado, receiving 1 or 3 reminder/recalls were associated with greater likelihood of initiation and completion.
Szilagyi et al., 2015 [162]	United States<br><br>The study was based in both a local and a national care network: Primary care practices in the Greater Rochester practice-based research network (GRPBRN) and pediatric continuity care clinics in the national Continuity Clinic Research Network (CORNET)	N = 1600 (GRPBRN), 1920 (CORNET)<br>Boys and girls<br>11-17 years	GRPBRN<br>Race = NR<br>CORNET<br>Black = 36-38%<br>White = 20-25%<br>Hispanic = 11-19%<br>Other = 4-6%<br><br>Health insurance<br>GRPBRN<br>Private = 15-20%<br>Public = 76-84%<br>Uninsured/military = 2-5%<br>CORNET<br>Private = 61-63%<br>Public = 29-35%<br>Uninsured/military = 4-8%	GRPBRN = 85 practices<br>CORNET = 73 practices<br><br>Provider prompts, vaccine access	Provider prompts were implemented. Electronic prompts were updated to display a list of needed vaccines at all health care visits instead of only preventive care visits. Providers at clinics using nurse/staff prompts were given vaccine information by staff at all visits.	Uptake, time to any dose, missed opportunities	There were few effects of the intervention on uptake except that adolescents in the GRPBRN were 124% more likely to receive dose 2 than control practices and with less time following dose 2. Additionally, missed opportunities reduced by 18% in the GRPBRN.
Vinci et al., 2022 [117]	United States<br><br>Pediatric clinics in a federally qualified health center organization in Charlotte, Hendry, and Lee Counties in Southwest Florida	N = 11,191 (8960 after intervention implemented)<br>Boys and girls<br>11-16 years	Race = NR<br>SES = NR	N = 9 practices<br><br>Provider prompts, reminder/recall, vaccine access, performance feedback	Clinics implemented use of the state electronic record system, automated prompts on patient forms and in the system, and postcard reminders on the adolescents' 11th and 12th birthday. Clinics also provided vaccine only appointments.	Uptake of dose 1, dose 2	The intervention was associated with increased uptake of dose 1 but decreased uptake of dose 2.

**Results indicate intervention not associated HPV vaccine outcomes**							
Calo et al., 2019 [163]	United States<br><br>North Carolina, Michigan, Iowa, Kentucky, Oregon	N = NR<br>Gender = NR<br>11-17 years	Race = NR<br>SES = NR	N = 15 pharmacies (North Carolina = 2; Michigan = 10; Iowa = 2; Kentucky = 1; Oregon = 0)<br><br>Vaccine access	Community-based HPV vaccination service sites were implemented in pharmacies in five states. In North Carolina, a pharmacist/nurse screened for eligibility, reviewed the adolescent's immunization record when a parent expressed interest, and provided same-day vaccine. In Michigan, a screening tool and educational handout was placed in prescription bags and same-day vaccine was offered. In Iowa and Kentucky, pharmacies administered the first dose and transmitted prescription orders for second and third doses to partnering pharmacies. No pharmacies were recruited in Oregon.	Service penetration	All pharmacy sites reported little-to-no service penetration. Although parents found the sites acceptable, some pharmacy staff and associated physicians were resistant to administering HPV vaccines.
Chantler et al., 2020 [179]	England<br><br>Secondary schools in seven South London boroughs	N = 3219<br>1733 (intervention), 1486 (control)<br>Girls<br>12-14 years	Race = NR<br>SES = NR	N = 14 schools<br><br>Consent procedures	Nurse-led intervention team provided schools with vaccination letters for parents which included a link to an online portal. Parents provided e-consent. The online portal indicated parents as consented, missing information or had questions, or non-consenting. Control schools and community catch-up clinics used paper consent procedures.	Uptake	There was no significant difference in uptake between paper consent and e-consent procedures.
Groom et al., 2017 [153]	United States<br><br>The Center for Health Research within Kaiser Permanente Northwest	N = NR<br>Boys and girls<br>11-18 years	Race = NR<br>SES = NR	N = 9 practices<br><br>Performance feedback	Feedback was provided on coverage rates and missed opportunities.	Uptake	Little change was observed for girls (71-72%) and boys (65-68%) likely due to practices in place prior to intervention.
Hofstetter et al.,	United States	N =295	Latino = 82%	N = 4 practices	Parents received a maximum of	Uptake	There was no difference in uptake

2017 [122]	Pediatric primary care clinics affiliated with an academic medical center	154 (intervention), 141 (control)<br>Boys and girls<br>11-17 years	NH Black = 13%<br>NH White = 5%<br>Other/multi = 3%<br><br>Health insurance<br>Public = 93%<br>Private = 7%<br>Uninsured = .3%	Reminder/recall	five text messages and additional one or two "booster" messages. Those in the educational condition received messages that included information about infection risk, vaccine safety/efficacy, and physician recommendations and one message where parents could reply and get additional information on a selected topic. The control group received standard ("plain") reminder messages.		between study arms.
Jacobs-Wingo et al., 2017 [123]	United States<br><br>IHS, tribally-operated, and urban Indian healthcare facilities in five regions: Great Plains, Nashville, Navajo, Oklahoma, and Portland	N = 12,533<br>6,239 (intervention), 6,294 (control)<br>Boys and girls<br>13-17 years	Race = NR<br>SES = NR	N = 14 practices<br><br>Provider prompts, vaccine procedures, standing orders, vaccine access, initiation age, reminder/recall	Intervention sites implemented at least one best practice strategy including electronic provider reminders, bundles vaccines (HPV, Tdap, meningococcal), standing orders, walk-in or nurse immunization clinics, community vaccine events at schools and health fairs, recommending vaccines at age 9 and 10, reminder cards and magnets, and phone call reminders (initiating or enhancing). The control group consisted of top ranking IHS, tribally-operated, and urban Indian healthcare clinics ("best practices clinics").	Uptake	Eight of the ten intervention sites implemented strategies to increase uptake; all eight started or enhanced reminder/recall activities using phone calls, patient reminder magnets, and postcard/letter reminders. However, intervention sites performed worse than best practice sites on uptake for dose 1 (46% vs. 78%) and dose 3 (48% vs. 19%).
Koskan et al., 2022 [174]	United States<br><br>Federally qualified health center clinic	N = 31<br>14 (intervention), 17 (control)<br>Boys and girls<br>9-17 years	Race = NR<br>SES = NR	N = 1 practice<br><br>Vaccine access	The intervention implemented a Shared Responsibility Model where dose 1 was administered by a primary care provider and dose 2/3 was administered by a pharmacist. The control group completed the vaccine series at their primary care clinic.	Completion	Both groups had 100% HPV vaccine completion. The intervention group demonstrated low adherence to protocol; only 3 (38%) returned to the pharmacy whereas 5 (63%) went to their primary care provider.
Musto et al., 2013 [175]	Canada<br><br>Calgary Zone, including the urban	N = 26,304 (In-school model), 9288 (Community model)<br>Girls	Race = NR<br>SES = NR	N = NR<br><br>Vaccine access, patient invitation	Vaccine appointments at community-based public health clinics were provided to girls enrolled in public or private	Completion	Completion among in-school girls was 75% compared to 36% among girls in the intervention (community service model). Odds of uptake was

	city of Calgary and surrounding rural areas with three large publicly funded school boards: the Calgary Board of Education, Rocky View Schools, and Calgary Catholic School District	9-11, or grade 5<br>13-15, or grade 9			schools that did not permit school-based vaccination. Parents were sent initiation letters that provided information about the vaccine and indicated where they could receive their HPV vaccinations		4x greater in the school model.
Oliver et al., 2019 [124]	United States<br><br>The New York City Department of Health and Mental Hygiene quality improvement program for middle school- and high school-based clinics operated by 10 organizations participating in the Vaccines for Children program	N = NR<br>Boys and girls<br>13-17 years	Race = NR<br>SES = NR	N = 24<br>school-based health centers<br>all eligible high schools (n = 19); 5 middle schools<br><br>Reminder/recall, standing orders, immunization registry	Several evidence-based strategies were implemented including reminder/recalls, standing orders, follow-up scheduling, and utilizing the immunization registry.	Initiation, completion	Implementation of strategies at the middle school and high school health clinics did not result in significant increases in initiation or completion rates.
Rand et al., 2023 [154]	United States<br><br>Primary care pediatric practices through the American Academy of Pediatrics Pediatric Research in Office Settings national research network and two large health systems in Minnesota and Texas	N = 100,752<br>46,540 (intervention),<br>54,212 (control)<br>Boys and girls<br>11-17 years	White = 62%<br>Hispanic = 17%<br>Black = 10%<br>Asian/Pacific Islander = 5%<br>American Indian/Alaska Native = 1%<br>Other/Multiple = 1%<br><br>SES = NR	N = 48 practices<br>24 (intervention),<br>24 (control)<br><br>Provider prompts, performance feedback, vaccine access	A combination of electronic and staff prompts was implemented. Administering dose 2 was also encouraged during acute/chronic care visits. Control practices provided standard care.	Missed opportunities	There was an increase in the number of missed opportunities for the HPV vaccine. The magnitude of increases in missed opportunities was 5% smaller for well visits.
Rehn et al., 2016	Sweden	N = 325,229	Race = NR	N = 21 county	The county launched a smart	Uptake of catch-	There was wide variation in uptake

[164]	Regional health offices in all 21 counties	Girls<br>Born between 1993 and 1998	SES = NR	health care offices<br><br>Media/advertisement campaign, reminder/recall, vaccine access	phone application that provided written and visual information as well as dose reminders. Additionally, the county launched a webpage. Oral and written information was provided in other languages. Media coverage occurred through press releases, newspapers, booklets, posters, interviews with health professionals on the radio, TV, and social media. All county schools implemented vaccine programs. Nurses also toured schools across the county. Other outlets for vaccine delivery were implemented (private vaccination centers, primary health clinics, or midwife clinics).	up doses	after strategies were implemented, but none explained differences in county-level vaccine uptake. The highest rate of uptake was observed in counties with county-wide school-based delivery. Counties that offered the vaccine to catch-up groups in schools reported significantly higher vaccine uptake.
Taddio et al., 2024 [180]	Canada<br><br>Community Health Centres in Calgary, Alberta serving jurisdictional schools from two school boards	N = 8839<br>Boys and girls<br>11-14 years	Race = NR<br>SES = NR	N = 105 practices<br>50 (intervention), 55 (control)<br><br>Patient comfort	Preventive pain management was implemented. Strategies including provider education, immunization spaces (e.g., separate waiting and injection areas, obscuring needles, and sharps containers), and self-selected coping strategies (e.g., immunization in privacy, presence of a support person, and distraction items). Control schools followed usual practices.	Uptake	The intervention was not associated with any significant difference in uptake.

N = sample size; NR = not reported; NH = non-Hispanic; SES = socioeconomic status; HPV = human papillomavirus.
High school diploma may also include completion of GED equivalency diploma.
*Based on parent, adolescent, or practice data reported.

Supplementary Table S9. Summary of Multilevel Studies (N = 53).

		Adolescent Targets		Intervention/Strategy			
Reference	Study Location/ Setting	Gender & Age	Race* & SES*	Modifiable Targets	Procedures/Description	HPV Vaccine Outcome	Findings
**Parent- and Provider-level Interventions (N = 9)**							
**Results indicate intervention associated improved HPV vaccine outcomes**							
Agana-Norman et al., 2022 [32]	United States<br><br>Vaccine data from NIS-Teen national survey	N = 97,587<br>49,644 (pre-intervention),<br>47,943 (post-intervention)<br>Boys<br>13-17 years	NH White = 55-58%<br>Hispanic = 20-22%<br>NH Black = 14%<br>Other/Multiple = 8-9%<br><br>Annual household income<br>>$75,000 = 42-46%<br>≤$75,000 = 34-37%<br>Below poverty = 21%	National campaign reached 3800 providers<br><br>Parent knowledge/attitudes, media/advertisement campaign, provider communication/ recommendation	A Centers for Disease Control and Prevention campaign was rebranded to focus on provider communication and parent attitudes. The campaign aimed to counteract negative parent attitudes by linking HPV vaccination to cancer prevention and other health preventive behaviors (e.g., wearing a helmet while bicycle riding). Parent campaign materials included interactive digital ads and a series of parent testimonial videos that were online and displayed in health care settings. Providers were encouraged to use presumptive recommendations. Provider campaign materials were distributed through webinars, clinician-targeted ads, brochures, and posters.	Initiation, completion of 3 doses	Initiation was 4x higher and completion was 6x higher following the campaign.
Cates et al., 2018 [48]	United States<br><br>North Carolina - Protect Them study	N = 147,294<br>19,398 (intervention),<br>127,896 (control)<br>Boys and girls<br>9-14 years	White = 44-50%<br>Black or African-American = 25-30%<br>Other =1-22%<br>Asian = 2%<br>American Indian/Alaska Native = 1-3%<br>Native Hawaiian/Other Pacific Islander = <1%	N = 14 practices<br><br>Parent knowledge/attitudes, provider education, provider communication/ recommendation	Parent/child dyads tested the acceptability and efficacy of an intervention game model. Practices involved in the study were asked to commit 50% of providers to training. Providers received education on HPV epidemiology and communication.	Initiation, completion of 3 doses	The intervention was associated with significantly higher rates of initiation and completion compared to baseline.

			Health Insurance<br>Private = 36-42%<br>Public = 39-49%<br>Uninsured = 8-10%				
Dempsey et al., 2018 [49]	United States<br><br>Sixteen family or pediatric medical practices in the Denver, Colorado metropolitan area that were part of a 30-clinic research network	N = 43,132<br>21,240 (intervention),<br>21,892 (control)<br>Boys and girls<br>11-17 years	White = 55%<br>Hispanic = 12%<br>Black = 5%<br>Other = 8%<br><br>Health Insurance<br>Private = 65%<br>Public = 31%<br>Other = <1%<br>None = 4%	N = 188 providers<br><br>Parent knowledge/attitudes, provider communication/ recommendation	The intervention included a parent education website that provided tailored information about HPV vaccination, a fact sheet library about HPV infection and vaccination, images depicting diseases associated with HPV, and a decision aid to encourage HPV vaccination. Providers received communication training comprised of a 30-minute self-guided training and two 1-hour in-person group sessions promoting presumptive recommendations.	Initiation, completion	The intervention was associated with significantly higher odds of initiation and completion.
Jafari et al., 2020 [50]	United States<br><br>Four clinics affiliated with Women's Health Center in rural Maryland	N = 635<br>317 (pre-intervention)<br>318 (post-intervention)<br>Girls<br>12-26 years	Race = NR<br>SES = NR	N = 7 providers<br><br>Parent knowledge/attitudes, reminder/recall, provider communication/ recommendation	A fact sheet was distributed to parents. Providers were trained to provide a direct recommendation.	Completion	There was a significant association between timing of the intervention and increased vaccine administration from 81% to 82%.
Strasel et al., 2024 [51]	United States<br><br>Two outpatient family medicine care clinics within a large primary care network in Grand Rapids, Michigan	N = 367<br>Boys and girls<br>9-10 years	Race = NR<br><br>Health insurance<br>Private = 76%<br>Public = 23%<br>None = 1%	N = 2 practices<br><br>Provider education, provider communication, parent knowledge/attitudes	The intervention included provider education focused on HPV recommendations for adolescents ages 9 and 10 years. Parents with children eligible for the vaccine received direct messages on MyChart that included vaccine information, addressed frequently asked questions about the HPV vaccine, and provided instructions for scheduling HPV vaccination via nurse visit or at their next appointment.	Uptake of dose 1	From pre-intervention to the provider education intervention, uptake of dose 1 significantly increased from .5% to 2.5%. From provider education to the direct messaging intervention, initiation significantly increased from 2.5% to 12.8%.

Suryadevara et al., 2019 [52]	United States<br><br>6 large pediatric offices in upstate New York	N = 1900 to 6000 per clinic<br>Boys and girls<br>11-18 years	Race = NR<br>SES = NR	N = 26 providers<br><br>Parent knowledge/attitudes, provider education, provider communication/ recommendation	The intervention included a year-long distribution of booklets that framed the HPV vaccine as cancer prevention. Providers participated in an 1-hour education focused on HPV infection-associated cancer epidemiology, current HPV vaccine standards, and preferred recommendation styles.	Initiation, completion	Across the 6 clinics, 5 increased initiation rates by 5%, and 4 increased completion rates by 5%.
Suryadevara et al., 2021 [53]	United States<br><br>Pediatric and family medicine practices across upstate and western New York.	N = 31,408<br>Boys and girls<br>11-18 years	Race = NR<br>SES = NR	N = 11 practices<br><br>Parent knowledge/attitudes, provider education, provider communication/ recommendation	The intervention included a year-long distribution of booklets that framed the HPV vaccine as cancer prevention. Providers participated in an 1-hour education focused on HPV infection-associated cancer epidemiology, current HPV vaccine standards, and preferred recommendation styles.	Initiation, completion	Intervention was associated with significant increases in initiation and completion 1 year after the intervention.
**Results indicate intervention associated with mixed HPV vaccine outcomes**							
Cates et al., 2014 [33]	United States<br><br>Thirteen counties in North Carolina	N = 25,869 total counties<br>19,842 (intervention counties), 6027 (control counties)<br>Boys<br>9-13 years	Control counties<br>White = 40%<br>Black = 31-47%<br>Other = 8-11%<br>Hispanic = 5-10%<br>American Indian/Alaska Native = 1-8%<br><br>64-66% of counties in intervention area eligible for Vaccination For Children program	N = 19,842 counties<br><br>Parent knowledge/attitudes, media/advertisement campaign, provider communication/ recommendation	A social marketing campaign began 3 months before the school year. Two public service announcements on HPV vaccination for boys were ran in addition to radio ads. Posters and brochures for parents were distributed to providers. Providers received a 1-hour communication training and a 1-page tip sheet.	Vaccination rates, missed opportunities	The intervention was associated with a significantly higher level of vaccination, but this effect was not sustained post-intervention. Missed opportunities remained lower in intervention counties 6 months post-intervention.
Cates et al., 2011 [71]	United States<br><br>Four counties (urban - Cumberland, rural - Robeson, Harnett,	N = 174<br>Girls<br>9-18 years	Based on survey sample:<br><br>White = 64%<br>Black = 20%	N = 213<br>parents in survey sample, 112 practices<br><br>Parent	A social marketing campaign was developed to engage a mother's instincts to protect her daughter from harm and decision-making regarding daughters' futures.	Self-report vaccination based on survey; county-level vaccination rate	Mothers aware of the campaign were more likely to vaccinate their daughters than mothers not aware. 27% mothers reported vaccination. County-level changes in

	Richmond) in the South Central Partnership for Public Health region of North Carolina		American Indian = 10%<br>Other = 6%<br><br>Annual Household Income<br><$25,000 = 16%<br>$25-75,000 = 60%<br>>$75,000 = 24%	knowledge/attitudes, media/advertisement campaign, provider communication/ recommendation	Print-based materials included two messages: "Help protect your daughter from cervical cancer. Vaccinate today. Protect her tomorrow;" "You have hopes and dreams for her future, and they don't include cervical cancer." Primary distribution included health care providers with posters and brochures, guide to "starting the conversation" with parents, buttons, and sticky notes to act as provider prompts, and community locations targeting parent populations such as pharmacies, beauty salons, grocery stores with posters and brochures. Secondary distribution included a project website, toll-free hotline, media releases, and radio public service announcement.		vaccination uptake were inconclusive.
**Parent- and Practice-level Interventions (N = 6)**							
**Results indicate intervention associated improved HPV vaccine outcomes**							
Daniel et al., 2021 [54]	United States<br><br>Rural community pharmacy in Alabama	N = 89<br>Girls<br>10-18 years	NH Black = 63%<br>NH White = 34%<br>Other = 3%<br><br>Health insurance Enrolled in Vaccines for Children program = 72%	N = 1 pharmacy<br><br>Parent knowledge/ attitudes, reminder/recall, media/advertisement campaign, vaccine access, standing orders	The intervention included a health communication campaign for parents comprised of digital and print materials to target knowledge and awareness of HPV and the vaccine. Mailers were sent to all households with an adolescent ages 10-18 years. Large posters were displayed in community spaces. Informational pamphlets and cards were stapled to prescription bags. Follow-up cards were distributed to adolescents who were vaccinated to remind them when to come back for their next dose. A social media campaign was also implemented. The intervention also included the creation of a Vaccine for Children authorized pharmacy, establishing physician	Uptake	HPV vaccination increased from 27% to 34%.

					standing order for HPV vaccination, and reporting to the Alabama's state immunization registry. Prior to the start of the school year, a community back to school vaccine clinic and block party was held at the pharmacy were HPV educational information and school supplies were distributed.		
Kaul et al., 2019 [55]	United States<br><br>Rio Grande Valley of Texas	N = 2307<br>885 (intervention), 1422 (control)<br>Boys and girls<br>9-14 years	Race = NR<br>SES = NR	N = 3 schools<br>1 intervention school, 2 control schools<br><br>Parent knowledge/attitudes, vaccine access	The intervention implemented a school-based HPV vaccination program that provided HPV vaccination to students at one school (intervention school) and included educational presentation events for parents at all three schools. Vaccination occurred at five events during the school year including<br>back-to-school events, progress report nights, and scheduled preview events. The educational presentations were given at school-based events including health fairs, vaccination days, back-to-school nights, Parent-Teacher Association [PTA] and school board meetings, and monthly nurse meetings, and community events including health department events, regional conferences, and training sessions/workshops.	Initiation, completion	Initiation (54% vs. 42%) and completion (28% vs. 21%) were significantly higher at the intervention school compared to the control schools following the intervention. On-site vaccination and community-based education was associated with 4x greater odds of initiation and completion.
Stubbs et al., 2014 [56]	United States<br><br>6 regions in Guilford County, North Carolina	N = 189<br>Girls<br>10-17 years	Race = NR<br>SES = NR	N = 21 schools<br>6 host schools, 15 satellite schools<br><br>Parent knowledge/attitudes, vaccine access	The education campaign included a web campaign on the county department of health website, a local media campaign, and automated calls to parents. The intervention also included a vaccination campaign that implemented temporary school-	Initiation, completion	Girls at schools providing vaccination were significantly more likely to be vaccinated than girls at satellite schools. Of all girls who received vaccination at a school clinic, 80% completed the series.

					located clinics that hosted 4 one-day clinics that provided HPV vaccine.		
Tran et al., 2022 [57]	France<br><br>Southern Reunion Island located in the priority education zone	N = 176<br>89 (intervention), 87 (control)<br>Boys and girls<br>9-16 years	Race = NR<br>SES = NR	N = 2 schools<br><br>Parent knowledge/attitudes, vaccine access	A school-based health promotion program was established to provide information to students during school classes, information to parents by letter and phone calls to obtain oral consent, information to general practitioners by letter and video conference call. Vaccines were administered to students in a "health bus."	Initiation, completion	Significantly more adolescents in the intervention initiated and completed the vaccine series than adolescents in the control.
**Results indicate intervention associated with mixed HPV vaccine outcomes**							
Tiro et al., 2015 [65]	United States<br><br>Parkland Health and Hospital System, the safety net for Dallas County in Texas	N = 814<br>Girls<br>11-18 years	Hispanic = 68%<br>NH Black = 28%<br>NH White = 3%<br><br>Health insurance<br>Private = 2%<br>Public = 74%<br>None = 24%	N = 4 practices<br><br>Parent knowledge/attitudes, reminder/recall, standing orders	Parents were mailed brochures discussing HPV risk, vaccine efficacy, perceived barriers to vaccination, and parent safety concerns along with an invitation letters. Nurses contacted parents who declined dose 1 two weeks after the visit, read a script indicating that the practice's providers strongly recommend the vaccine and offered a nurse-only immunization appointment. Control participants were only contacted to complete a follow-up survey. Parents in the intervention were contacted again for dose 2 or 3 if overdue by 4 weeks. The nurse followed a script to stress the importance of complete vaccination and offered a nurse-only immunization appointment.	Initiation, completion	There was no main effect of the mailed materials or follow-up for dose 1. However, those in the intervention were more likely to complete the series than those in the control.
**Results indicate intervention not associated HPV vaccine outcomes**							
Davies et al., 2023 [86]	Australia<br><br>Schools in Western and	N = 6967<br>Boys and girls<br>Avg. of 13.7 years	Race = NR<br>SES = NR	N = 40 schools<br>21 intervention schools, 19 control schools	Control schools utilized standard practice procedures including consent forms, vaccination room set-up, and catch-up vaccinations.	Initiation, completion	The intervention was not associated with uptake of any dose.

	South Australia			Parent knowledge/attitudes, parent intention/decision-making, communication with child/others, vaccine procedures	Intervention schools additionally followed adolescent in-class education and vaccination-day guidelines, sent adolescents home with consent forms and a decisional support tool booklet to support shared parent-adolescent decision, and set in-school catch-up of missed doses and vaccination-day guidelines.		
**Provider- and Practice-level Interventions (N = 25)**							
**Results indicate intervention associated improved HPV vaccine outcomes**							
Berenson et al., 2019 [91]	United States<br><br>Pediatric clinics in the University of Texas Medical Branch	N = 2162<br>Boys and girls<br>9-17 years	Hispanic = 34.4<br>White = 34.1<br>Black = 29.2<br>Asian/other = 2.3<br><br>SES = NR	N = 8 practices<br><br>Provider education, patient navigators	Providers participated in an education program on HPV vaccination. Practices implemented patient navigators that informed parents of children's eligibility for the vaccine, obtained consent, scheduled appointments and coordinated vaccine visits with other medical appointments, and sent reminders.	Completion	Overall, completion among initiators and prior starters was 92% after the intervention. Initiators had a completion rate of 93% and prior starters had a completion rate of 85%.
Bernstein et al., 2022 [127]	United States<br><br>Private pediatric practice in New England	N = 128<br>73 (pre-intervention), 55 (post-intervention)<br>Boys and girls<br>11-12 years	Race = NR<br>SES = NR	N = 1 practice<br><br>Provider education, provider communication/ recommendation, vaccine procedures, patient prompt	Providers participated in an educational session that included using presumptive recommendations and evidence-based responses to parent questions. Office polices were implemented to bundle the HPV vaccine with the tetanus, diphtheria and pertussis booster, and meningococcal vaccines and to send pre-visit emails to all families of 11 and 12 year old patients with well-child visits.	Uptake	Vaccination rate significantly increased from 18% to 64%.
Biehl & Efre, 2023 [128]	United States<br><br>A suburban primary care pediatric office in Florida	N = 1374<br>Boys and girls<br>13-17 years	NHH White = 77%<br>NH Black = 17%<br>Other = 6%<br><br>Health Insurance	N = 1 practice<br><br>Provider education, vaccine procedures, provider	Providers learned how to recommend the HPV vaccine. The practice implemented bundling the HPV vaccine with two other adolescent vaccines and	Uptake of dose 1, 2, and 3, Missed opportunities	Uptake of all doses increased after the intervention by 6%-11%. An 11% increase in dose 1 was a significant change from pre- to post-intervention. Missed

			Public/uninsured = 34%<br>Private = 66%	communication/ recommendation, provider prompts, standing orders	encouraged providers to give a strong recommendation for all three vaccines. Additionally, the practice implemented provider prompts and standing orders.		opportunities decreased by 4%.
Bonville et al., 2019 [106]	United States<br><br>Clinics affiliated with New York Chapter 1 of the American Academy of Pediatrics	N = 1048 (71-82 patient charts each cycle)<br>Boys and girls<br>11-12 years	Race = NR<br>SES = NR	N = Phase 1: 5 practices, Phase 2: 8 practices<br><br>Provider education, provider communication/ recommendation, reminder/recall, provider prompts, standing orders	Providers participated in an educational training on HPV epidemiology and giving presumptive recommendations. Practices implemented evidence-based strategies including reminder/recalls, provider prompts, and standing orders.	Completion, Missed opportunities	During Phase 1 of the intervention, completion rates increased from 59% to 78% and missed opportunities fell to zero for well visits and nurses visits. In Phase 2, completion rates increased from 62% to 94%.
Casey et al., 2022 [129]	United States<br><br>Federally qualified health center practices in the Boston metropolitan area	N = 16,612<br>8099 (pre-intervention),<br>8513 (post-intervention)<br>Boys and girls<br>10-16 years	Hispanic = 71-79%<br>White = 13-20%<br>Other = 6%<br>Black = 2%<br>Asian = 2%<br><br>SES = NR	N = 5 practices<br><br>Provider education, provider communication/ recommendation, performance feedback, initiation age	Providers participated in a continuing education program focused on HPV vaccination and communication training. The practice also implemented performance feedback. Providers were given the option to administer the vaccine to adolescents younger than 11 years.	Initiation, Completion	Initiation increased from 72% to 92% for adolescents ages 11-12 years and from 96% to 97% for adolescents ages 13-18 years. Completion increased from 62% to 88% before age 13, and from 89% to 91% for adolescents ages 13-18 years. Increases were statistically significant.
Charles & Erikson, 2023 [130]	United States<br><br>Private family practice in Bucks County, Pennsylvania	N = 15<br>10 (pre-intervention), 5 (post-intervention)<br>Boys and girls<br>11-15 years	Race = NR<br>SES = NR	N = 1 practice<br><br>Provider education, provider communication/ recommendation, vaccine procedures, clinic materials	Providers participated in the "You Are the Key to HPV Cancer Prevention" education course and were encouraged to give a standard recommendation of the HPV vaccine in the same manner as other adolescent vaccines. The practice implemented vaccine bundling. Other strategies included materials in waiting rooms and examinations rooms and sending reminder letters to patients due for the HPV vaccine.	Uptake	Vaccination increased by 400% following the intervention. Prior to the intervention, 1 adolescents was vaccinated. Following the intervention 5 adolescents were vaccinated.
Gilkey et al., 2022 [131]	United States	N = 98,682<br>Boys and girls	Race = NR<br>SES = NR	N = 267 practices	Providers in practices receiving QI (Quality Improvement)	Initiation, completion	At 6-months, initiation for 11 to 12 year olds was significantly higher

	Health departments serving a Midwestern state, a Southwestern state, and 3 large counties in a Northeastern state	11-17 years		Provider education, performance feedback, provider communication/ recommendation	coaching participate in 12 feedback sessions and developed an evidence-based strategy plan. Providers in practices with communication training participated in one virtual education session that focused on the presumptive recommendation style. The combined intervention included QI coaching and communication training.		among those visiting practices in the combined intervention. There was no significant association with QI coaching or communication training. At 12-months, initiation for 11 to 12 year olds was significantly higher in practices where QI coaching and the combined intervention were implemented. There was no significant association with communication training. For 13 to 17 year olds, only the combined intervention was significantly associated with initiation and completion at 12-months. At 18-months, initiation was significantly higher at practices with QI coaching or the combined intervention while communication had no significant effect.
Krantz et al., 2018 [132]	United States<br><br>Primary care center affiliated with the Cincinnati Children's Hospital Medical Center	N = NR<br>Boys and girls<br>13-17 years	NH Black = 74% NH White = 18%<br>Hispanic = 3%<br>Other = 4%<br><br>Health Insurance<br>Public = 80%<br>Self-pay = 10%<br>Private = 10%	N = 1 practice<br><br>Provider education, vaccine procedures, provider prompts	Providers participated in an educational seminar focused on vaccine communication. Practices also implemented vaccine scheduling procedures and visual prompts.	Uptake, Completion	Uptake significantly increased from 78% to 89% following the education and vaccine procedure interventions. Completion significantly increased from 51% to 62% following the intervention.
Mayne et al., 2014 [107]	United States<br><br>Children's Hospital of Philadelphia Pediatric Research Consortium	N = 17,016<br>Clinician+Family condition = 4190<br>Clinician condition = 4185<br>Family condition = 4356<br>Control = 4285<br>Boys and girls<br>11-17 years	White = 62%<br>Black = 26%<br>Other = 12%<br>Hispanic = 2%<br><br>Health insurance<br>Private = 82%	N = 22 practices<br><br>Provider education, performance feedback, provider prompts, reminder/recall	The intervention consisted of clinician-focused interventions including provider education, performance feedback, and provider prompts. Practices also implemented a family-focused intervention with automated reminder calls to families with an adolescents needing the vaccine.	Vaccine opportunities for doses 1, 2, and 3	Overall, clinician-only strategies were more effective than the automated reminder calls at increasing vaccine opportunities. Opportunities for dose 1 increased by 9% with the clinician strategies and by 10% with the combined intervention. The intervention was not associated with increased opportunities for dose 2. Opportunities for dose 3 were increased by 6% with the clinician

							strategies and by 3% with the combined intervention.
Meyer et al., 2018 [133]	United States<br><br>Retail care clinics owned by Mayo Clinic in shopping areas of Southeast Minnesota	N = 3234<br>1469 (pre-intervention),<br>1765 (post-intervention)<br>Boys and girls<br>9-26 years (avg. of 14.11 years)	White = 86%<br>Hispanic = 4%<br>Asian = 3%<br>Black = 3%<br><br>SES = NR	N = 2 practices<br><br>Provider education, provider prompts, vaccine procedures, patient prompts	Retail clinic staff including certified nurse practitioners and licensed practical nurses participated in an educational session. Clinics also implemented the electronic point-of-care prompts. Print outs of recommended vaccinations were given to parents.	Uptake	Uptake significantly increased from 2% to 12%.
Molokwu et al., 2023 [35]	United States<br><br>Academic medical center near the US-Mexico border in El Paso, Texas	N = 2851<br>1561 (pre-intervention), 1290 (post-intervention)<br>Boys and girls<br>9-26 years (avg. of 17 years in pre-intervention, avg. of 18 years in post-intervention)	Hispanic = 77-81%<br><br>Health Insurance<br>Public = 65-69%<br>Private = 24-26%<br>Other = 8-9%	N = 84 providers across 3 departments (family medicine, pediatrics, obstetrics-gynecology)<br><br>Provider education, provider prompt	Providers received one educational lecture. The practice also implemented electronic provider prompts for all eligible patients.	Completion	The provider prompt was associated with a significant increase in completion.
Moore et al., 2022 [134]	United States<br><br>Midwestern tertiary academic children's hospital	N = 440<br>121 (pre-intervention), 319 (post-intervention)<br>Boys<br>≥13 years (avg. of 15.3 years)	Race = NR<br>SES = NR	N = 1 hospital<br><br>Provider education, vaccine procedures, patient prompts	Providers received two training sessions and the practice added immunization status information on after-visit summaries provided upon discharge from the hospital.	Uptake	Vaccination rates increased from 5% to 22% following implementation of the intervention.
Perkins et al., 2020 [135]	United States<br><br>Primary care pediatric and/or family medicine practices in the Boston, Massachusetts metropolitan area	N = 16,136<br>Boys and girls<br>9-17 years	Hispanic = 37-55%<br>NH Black = 20-30%<br>Other = 13%-18%<br>NH White = 12-15%<br><br>Health Insurance<br>Public = >80%	N = 5 practices<br><br>Provider education, provider communication/ recommendation, performance feedback, initiation age	Providers received education, communication training, and performance feedback. The practices changed the initiation age to before age 11 (ages 9 or 10).	Initiation, completion	For 9-10 year olds, initiation increased from 75% before the intervention to 84% during the intervention and 90% after the intervention. Completion increased from 60% before to 63% during and 69% after. Changes in initiation and completion were significant. For 11-12 year olds, initiation increased from 83% before the intervention to 89% during and 93% after. Completion increased

							from 54% to 53% during and 69% after. Changes in initiation and completion were significant.
Rand et al., 2018 [136]	United States<br><br>National, practice-based research network comprised of pediatric resident continuity practices called the Continuity Research Network (CORNET)	N = Avg. of 2224 per clinic<br>Boys and girls<br>11-17 years	Race = NR<br><br>Health Insurance<br>Public = 28%<br>Private = 29%<br>Uninsured = 8%	N = 8 practices<br><br>Provider education, provider communication/ recommendation, provider prompts, clinic materials	Providers received education on recommendations and communication about the vaccine. Provider prompts were implemented for acute and chronic care visits as to have prompts at every adolescent visit. Additional visual prompts including door signs and posters were posted in practices.	Uptake	Adolescents were more likely to receive an HPV vaccine dose during the intervention than before the intervention.
Smajlovic & Toth, 2023 [149]	United States<br><br>Nationwide Children's Hospital Primary Care Network in Columbus, Ohio	N = NR<br>Boys and girls<br>9-13 years	Race = NR<br>SES = NR	N = 12 practices<br><br>Provider communication/ recommendation, performance feedback, clinic materials, provider prompts, standing orders	The first intervention encouraged providers to use the announcement style recommendation. The second intervention included performance feedback emails every 6 months to 1 year. The third intervention included use of posters and handouts in the clinic. The fourth intervention implemented electronic prompts and standing orders where nursing staff can order and administer the HPV vaccine at the start of an eligible visit.	Completion	Completion rates increased from 27% to 46% following the implementation of performance feedback. Rates increased by 10% following a discussion of the Quality Improvement project. An additional 10% increase occurred after all providers were reminded to use the announcement approach and posters were placed in clinic rooms.
Zimmerman et al., 2017 [108]	United States<br><br>Primary care family medicine and pediatric practices from two practice-based research networks and a clinical network in the Pittsburgh metropolitan area in Pennsylvania	N = 10,861<br>4942 (intervention), 5919 (control)<br>Boys and girls<br>11-17 years	Non-White = 2%–98% (Differences between conditions were statistically significant)<br><br>Health insurance<br>Private = 65%	N = 20 practices (Intervention = 9, Control = 11)<br><br>Provider education, vaccine procedures, standing orders, reminder/recall, provider communication/ recommendation	The intervention was designed using the 4Pillars™ Model which had 4 domains: (1) Convenient vaccination services, (2) Communication with patients, (3) Enhanced office systems, and (4) Motivation through an office immunization champion and on-on-one coaching. Strategies implemented included vaccination at every opportunity, standing order protocols for nursing staff,	Initiation, completion	Initiation was significantly higher in the intervention group than for the control group. Completion was not significantly different between groups. However, completion in the intervention was significantly higher after the intervention.

					reminders to patients to return for subsequent doses, and office-wide recommendations to parents/patients to be vaccinated. Practices in the control group did not participate in the intervention.		
Zimmerman et al., 2017 [109]	United States<br><br>Primary care family medicine and pediatric practices from two practice-based research networks and a clinical network in the Pittsburgh metropolitan area in Pennsylvania	N = 9473<br>Boys and girls<br>11-17 years	One-third were non-White<br><br>Two-thirds had private insurance	N = 11 practices<br><br>Provider education, vaccine procedures, standing orders, reminder/recall, provider communication/ recommendation	The intervention was designed using the 4Pillars™ Model which had 4 domains: (1) Convenient vaccination services, (2) Communication with patients, (3) Enhanced office systems, and (4) Motivation through an office immunization champion and on-on-one coaching. Strategies implemented included vaccination at every opportunity, standing order protocols for nursing staff, reminders to patients to return for subsequent doses, and office-wide recommendations to parents/patients to be vaccinated. Practices in the control group did not participate in the intervention.	Initiation, Completion	Initiation and completion significantly increased after the intervention was implemented. Initiation was significantly higher for younger adolescents. There were no differences in completion between age groups.
**Results indicate intervention associated with mixed HPV vaccine outcomes**							
Berenson et al., 2020 [92]	United States<br><br>Pediatric clinics in the University of Texas Medical Branch	N = 21,395<br>Boys and girls<br>9-17 years	NH White = 41%<br>Hispanic = 35%<br>NH Black = 22%<br>Other = 2%<br><br>SES = NR	N = 8 practices<br><br>Provider education, patient navigators	Providers participated in an education program on HPV vaccination. Practices implemented patient navigators that informed parents of children’s eligibility for the vaccine, obtained consent, scheduled appointments and coordinated vaccine visits with other medical appointments, and sent reminders. Control clinics were non-program practices that did not have an onsite patient navigator.	Uptake	Intervention clinics had significantly higher odds of vaccination than control clinics. Specifically, rates for adolescents 9-12 years significantly improved, but did not for adolescents 13-17 years.
Glenn et al., 2022 [119]	United States	N = 14,738<br>5988 (intervention),	Hispanic = 88%<br>NH White = 7%	N = 8 practices	The intervention included provider and staff training,	Initiation, Completion	Initiation was significantly higher at intervention practices compared

	The largest federally qualified health center in the nation, which serves the Los Angeles and Orange counties in California	8750 (control)<br>Boys and girls<br>11-17 years	Other = 3%<br><br>Health insurance<br>Public = 72%<br>Self pay = 11%<br>Private = <1%	Provider education, performance feedback, vaccine procedures, standing orders, reminder/recall	performance feedback, and the implementation of systematic protocols that included several procedural changes standing orders, offering the HPV vaccine at each visit, visual provider prompts, and a check out procedure. Practices implemented paper logs to track patients due for follow-up doses and sent families reminder cards. Control practices provided usual care.		to usual care practices. Completion in both groups demonstrated an upward trend.
McLean et al., 2017 [120]	United States<br><br>A regional health care system in Central, Northern, and Western Wisconsin	N = 24,658<br>16,041 (intervention), 8617 (control)<br>Boys and girls<br>11-17 years	Race = NR<br>SES = NR	N = 7 practices<br><br>Provider education, provider communication/ recommendation, performance feedback, reminder/recall	Departments with the largest adolescent populations were selected for the intervention. Each department was visited at least three times for provider and staff education including training on providing recommendations. Resources were given for providers and patients and quarterly feedback was implemented. Practices also began to send reminder letters to every family with 12 year olds needing vaccines. The control group consisted of all other departments that provided primary care and vaccinations to adolescents at each practice.	Uptake, completion	Uptake was significantly greater for 11-12 year olds and for 13-17 year olds at intervention practices compared to control practices. The greatest increase occurred after provider education and implementation of reminder letters. There was no significant difference in intervention practices and control practices in completion rates.
Rand et al., 2018 [121]	United States<br><br>The National Improvement Partnership Network recruited state improvement partnerships, which then recruited community practices in	N = NR<br>Boys and girls<br>11-17 years	Race = NR<br>SES = NR	N = 47 practices (33 community-based practices, 14 continuity practices)<br><br>Provider education, provider communication/ recommendation, performance feedback, standing	Providers were training to reduce missed opportunities and provide strong recommendations. Providers also received feedback reports. Practices had the option to implement standing orders and/or reminder/recall procedures.	Initiation, completion	Initiation at community sites reported significantly higher initiation overall (62% to 70%), for boys (57% to 65%), and for girls (66% to 74%). Initiation at continuity sites were significantly higher overall (71% to 77%) and for boys (68% to 75%), but not for girls. Completion was significantly higher overall at community sites, and significantly higher for boys at

	Alabama, Maine, New Hampshire, New Jersey, Tennessee, and Vermont; and continuity practices associated with the Continuity Research Network (CORNET)			orders, reminder/recall			continuity sites.
Steiner et al., 2021 [142]	United States<br><br>Downtown, nurse-run walk-in immunization clinic affiliated with a county health department in Southwestern Pennsylvania	N = 209<br>115 (intervention), 94 (control)<br>Boys and girls<br>11-14 years	Race = NR<br>SES = NR	N = 1 practice<br><br>Provider education, vaccine procedures, clinic materials	Providers participated in an educational class which educated staff on vaccine protocols. The protocol script and immunization schedule were posted in all patient exam rooms, all restrooms, and the staff kitchen. Staff were also required to utilize the immunization registry to check immunization status.	Uptake	For all patients, uptake was not significantly higher after the intervention. For those initiating the vaccine, uptake significantly increased from 18% to 35%. Uptake among boys significantly increased, but not for girls.
Zorn et al., 2023 [143]	United States<br><br>Private pediatric clinics in King County, Washington	Clinic A = >4300<br>Clinic B = >44,500<br>Boys and girls<br>9-14 years	Race = NR<br><br>Health Insurance<br>Private = 85-93%<br>Public = 6-9%	N = 2 practices (Clinic A = 1 site, Clinic B = 8 sites)<br><br>Provider education, provider communication/ recommendation, initiation age, provider prompt, clinic materials	The intervention consisted of provider and staff training, a recommendation script, policy change requiring initiation at age 9, utilization of the electronic prompts, standardized immunization schedule poster for the practice, and HPV vaccine messaging via printed resources including a lobby poster, pamphlets, and information cue cards.	Initiation, completion	At Clinic A, initiation and completion increased for all age groups (9-10 year, 11-12 years, 13 years, 13-17 years). At Clinic B, initiation and completion increased for all age groups except adolescents 13 years old.
**Results indicate intervention not associated HPV vaccine outcomes**							
Davis et al., 2022 [147]	United States<br><br>Urban pediatric primary care clinic in a large community-based hospital system in the Pacific	N = 39<br>Boys and girls<br>11-12 years	NH Black = 33%<br>Latinx = 28%<br>NH White = 20%<br>Multiple/other = 16%<br>Asian = 3%<br><br>SES = NR	N = 1 practice<br><br>Provider education, provider communication/ recommendation, vaccine procedures	Providers participated in an educational program focused on five factors of a high-quality recommendation. Practices also implemented scheduling dose 1 and dose 2 during well-child visits for all 11 and 12 year olds.	Decline of vaccine	There was no significant effect of recommendation quality on decline rates.

	Northwest						
Fisher-Borne et al., 2018 [125]	United States<br><br>Federally qualified health center systems reaching >20,000 adolescents	N = >20,000<br>Boys and girls<br>11-12 years	Race = NR<br>SES = NR	N = 130 practices across 30 systems<br><br>Provider education, vaccine procedures, performance feedback, provider prompts, reminder/recall	Ten systems equaling 36 clinics implemented provider training. Ten systems equaling 37 clinics each received $10,000, implemented provider training, and updated their electronic health record system. Ten systems equaling 57 clinics each received $90,000, implemented provider training, implemented at least one evidence-based strategy including performance feedback, provider prompts, and patient reminders, and updated their electronic health record system.	Initiation, Uptake of dose 2	The intervention was not associated with significant change in initiation or uptake of dose 2.
**Parent-, Provider- and Practice-level Interventions (N = 13)**							
**Results indicate intervention associated improved HPV vaccine outcomes**							
Ahmed et al., 2022 [24]	India<br><br>Community intervention with school- and community health clinics in the North, East, South, and West districts of Sikkim State	N = 21,118<br>1591 (North), 6063 (South), 6107 (West), 7357 (East)<br>Girls<br>9-13 years	Race = NR<br>SES = NR	N = 2081 schools, 29 health facilities<br><br>Parent knowledge/attitudes, provider education, vaccine access	The Sikkim government introduced a multiple-age cohort, school-based HPV vaccine program for all girls ages 9-13 years. More than 1000 primary and secondary schools were included. Those who did not attend school could be vaccinated at school or local health facilities. All districts implemented cascaded training for health care providers, community health workers, and teachers. Community education programs were also created. The vaccination program implemented school-based vaccination sessions conducted in the first week of the vaccination campaign each year with vaccination opportunities at local health facility during the second and third weeks.	Uptake of dose 1 and 2	Uptake across the state was high: >95% coverage for dose 1 and >90% coverage for dose 2.
Bowden et al., 2017	United States	N = 265	NH Black = 85-86%	N = 1 practice	Education sessions were provided	Uptake	Uptake increased from 50% on

[58]	University-run urban clinic primarily serving Medicaid population in Tennessee	136 (pre-intervention), 129 (post-intervention)<br>Boys and girls<br>9-13 years	Hispanic = 5-7%<br>Other = 4-6%<br>NH White = 2-3%<br><br>Health Insurance<br>Private = 8-12%<br>Public = 86-92%<br>Uninsured = 0-2%	Parent knowledge/attitudes, provider education, provider communication/ recommendation, initiation age	to nursing staff and residents. Residents received three sessions on providing a recommendation. A standardized script for recommendations was implemented. Initiation age was changed from 11 years old to 9 years old. All parents of patients 9-13 years were provided information during visits to improve knowledge of the vaccine.		average to 62% following resident educational training, Following implementation of the recommendation script, uptake peaked at 100%. Uptake was reported to be 84% after the initiation age was changed to 9 years. Post-intervention uptake was 69% on average.
Cassidy et al., 2014 [59]	United States<br><br>Small private pediatric practice in an urban location	N = 23<br>Girls<br>11-12 years	NH White = 61%<br>NH Black = 35%<br>Asian = 4%<br><br>Income<br>≤$19,999 = 4%<br>$20,000-$39,999 = 26%<br>$40,000-$59,999 = 22%<br>$60,000-$99,999 = 17%<br>$100,000 = 17%<br>Unknown/refused = 13%	N = 1 practice<br><br>Parent knowledge/attitudes, provider education, provider communication/ recommendation, provider prompts, reminder/recall	The intervention included a brochure and one-on-one intervention for parents. The brief intervention occurred after a physical examination of the adolescent. Providers received educational training. The practice implemented a script for recommending the vaccine. Electronic alerts prompted reminder calls for parents.	Initiation, completion, uptake of dose 1, 2, and 3	Uptake increased from 24% to 75% with parents who received the intervention were more likely to be vaccinated than historical controls. The brief intervention was significantly associated with 9.4x greater odds of uptake than historical controls. Completion increased from 7% to 63% with the intervention conditions being associated with significantly greater completion than the historical control. The intervention was significantly associated with 22.5x greater odds of series completion.
Choi et al., 2018 [60]	United States<br><br>VFC program clinics, walk in immunization clinics, and federally qualified health centers in Chicago	N = NR<br>Boys and girls<br>9-18 years	Race = NR<br>SES = NR	N = 80<br>(37 federally qualified health centers, 30 private clinics, 12 school-based clinics, 1 public clinic)<br><br>Parent knowledge/attitudes, provider education, provider communication/ recommendation, performance	An HPV curriculum consisting of in-person training, dinner seminars, grand rounds lectures, and webinars was created for providers. Providers also received performance feedback on vaccine administration. Consistent vaccine messaging and recommendations were implemented. Recall procedures including mailed letters and postcards were sent to parents. A comprehensive public communication program was also	Initiation, completion	95% of practices indicated increases in initiation and 96% indicated increases in completion. Among adolescents 9-18 years, those with an adult reporting exposure to the campaign were more likely to be vaccinated that day. Population estimates for 2014 and 2015 indicate increased uptake of doses 1, 2, and 3 among adolescents.

				feedback, reminder/recall, media/advertisement campaign	implemented with broadcast (2 television networks, 3 local radio stations), print (newspapers, magazines), outdoor (6 billboards), and digital platforms (websites, social media toolkit).		
Cox et al., 2022 [61]	United States<br><br>A hospital-based clinic and community health center in Massachusetts	N = 12,270<br>Boys and girls<br>9-13 years	Hispanic = 45%<br>NH Black = 38%<br><br>Health Insurance Public = 70%	N = 2 connected pediatric primary care practices<br><br>Parent knowledge/attitudes, provider education, provider communication/ recommendation, standing orders, vaccine access, initiation age	Posters and handouts were created to address parent mistrust, concerns, and misconception about the HPV vaccine. Provider education focused on communication skills and “announcement” style recommendations. All providers also received a 45- minute training on motivation interviewing. Standing orders were implemented allowing nurses to administer the vaccine and a monthly evening shot clinic was implemented. Initiation age was changed to 9.	Initiation, completion	Initiation by age 9 increased from 1% to 52%, and completion by age 13 increased from 38% to 77%.
Dang et al., 2023 [62]	United States<br><br>A rural federally qualified health center (FQHC) located in Glenn County, California	N = 996<br>Boys and girls<br>11-17 years	Race = NR<br>SES = NR	N = 1 practice<br><br>Parent knowledge/attitudes, provider education, clinic materials, reminder/recall	Practice-wide training on the clinic’s HPV vaccination policies and procedures were conducted. Providers were given resources on HPV vaccine messaging. Posters and handouts were placed in all exam rooms. Reminder postcards were mailed to all active patients. The first postcard was mailed to patients turning 11 years and provided parents with information about the vaccine. The second postcard was mailed to patients ages 11-17 who initiated the HPV vaccine series but did not complete to emphasize the importance of completing the series. The third postcard was mailed to patients ages 11-17 who have not started the series with messaging to that emphasizes	Uptake	Initiation significantly increased from 53% to 83%, and completion significantly increased from 27% to 58%.

					initiation.		
Fiks et al., 2013 [63]	United States<br><br>The Children's Hospital of Philadelphia (CHOP) Pediatric Research Consortium	N = 22,486 (Family condition= 5680 Clinician condition = 5557 Combined condition = 5561 Control = 5688)<br>Girls<br>11-17 years	NH White = 55%<br>NH Black = 31%<br>Other = 12%<br>Asian = 2%<br><br>Health Insurance<br>Private = 80%	N = 22 practices<br><br>Parent knowledge/attitudes, provider education, performance feedback, reminder/recall	Providers participated in an in-person or online training session and were provided performance feedback quarterly. Electronic provider prompts were implemented for all routine adolescent vaccines. Automated telephone calls were made to families based on HPV vaccine status. Each call stated the vaccines due, emphasized the importance of vaccination, and referred families to an educational website.	Uptake	The intervention was associated with significantly higher uptake than control practices.
Giambi et al., 2015 [110]	Italy<br><br>17 regions in Italy - 40% in the North, 32% in the South and 28% in the Centre of Italy.	N = NR<br>Girls<br>11 years	Race = NR<br>SES = NR	N = 133 local health authorities<br><br>Parent knowledge/attitudes, provider education, reminder/recall, patient invitation, vaccine access	Providers received professional training and performance feedback. Practices implemented contact protocols, an information hotline, and established vaccine-only clinic sessions. Communication with parents included reminder/recall phone calls, invitation letters, and pre-vaccination counseling.	Uptake	Factors associated with significantly higher probability of uptake ≥ 70% were a pre-assigned date for HPV vaccination, more than one recall contact, and more than three communication channels used to promote vaccination.
Huang et al., 2024 [64]	United States<br><br>Large pediatric primary care network across 2 states in both urban and suburban settings	N = NR<br>Boys and girls<br>9-12 years	Race = NR<br>SES = NR	N = 30 practices<br><br>Parent education/attitudes, provider education, provider communication/ recommendation, performance feedback, vaccine procedures, clinic materials, provider prompts, initiation age, standing orders	Pre-visit phone calls to parents with the objective of preparing families for vaccines were implemented in response to the COVID-19 pandemic. Providers received webinar-based educational training and performance feedback. Providers were also encouraged to participate in scripted motivational interviewing role-play scenarios via an interactive training application. One standard poster was displayed in all exam rooms. Electronic provider prompts were established and	Completion	Completion by age 13 increased from 30% to 55% following implementation of the intervention.

					initiation age was lowered to age 9. Standing orders were implemented for the initial dose.		
**Results indicate intervention associated with mixed HPV vaccine outcomes**							
Chung et al., 2015 [72]	United States<br><br>Phase 1: Intervention sites in Duplin County of North Carolina. Control sites included Columbus, Edgecombe, Pender, and Sampson.<br><br>Phase 2: Duplin County public school system	N = NR<br>Boys and girls<br>11-18 years	Race = NR<br>SES = NR	Phase 1 = 7 practices (4 family medicine practices, 2 pediatric practices, and the county health department)<br>Phase 2 = 12,420 parents of public school students<br><br>Parent knowledge/attitudes, provider education, reminder/recall, clinic materials, vaccine access	Providers participated in educational meetings focused on strategies for improving vaccine uptake. Reminder postcards were sent to families. Additional promotional posters were provided to practices. Practices were financially incentivized to utilize mailed reminders. At 6 months, a school-based intervention was implemented that involved educational telephone messages to parents in the public school system.	Initiation, completion	Initiation among 11-12 year olds was significantly higher in intervention counties compared to control counties. Completion was only significantly higher for boys ages 13-18 years in intervention counties.
Paskett et al., 2016 [73]	United States<br><br>12 counties in Appalachian Ohio	N = 337<br>174 (intervention), 163 (control)<br>Girls<br>9-17 years	White = 96%<br><br>Annual income<br><$30K = 19%<br>$30K–$69,999 = 44%<br>≥$70,000 = 37%	N = 24 clinics (10 intervention with 57 providers, 14 control with 62 providers)<br><br>Parent knowledge/attitudes, provider education, clinic materials	Parents received educational brochures and DVDs as well as telephone-based educational phone calls with health educators. Providers participated in a powerpoint education session. Practices posted waiting room and exam room posters and brochures.	Uptake, completion	The intervention was not significantly associated with initiation at 3-months follow up. At 6 months, initiation in the intervention was significantly higher than in the control condition. However, the intervention was not significantly associated with uptake of dose 2 or 3.
Sanderson et al., 2017 [74]	United States<br><br>Safety-net clinics in three cities in Tennessee, U.S.	N = 408<br>Boys and girls<br>9-18 years	Black = 87%<br>Hispanic = 13%<br><br>Monthly income<br>Intervention<br><$1,000 = 27-28%<br>$1,000-$1,999 = 34-46%<br>≥$2,000 = 18-28%	N = 4 practices<br><br>Parent knowledge/attitudes, provider education, initiation age, vaccine access	Study researchers created age-targeted educational videos for 9-12 year olds and 13-18 year olds about HPV as well as a brochure for parents. Providers received training on HPV and cancer screening procedures. Practices updated the eligibility criteria to include males and adjusted the eligible age range from 16-18 years to 9-18 years.	Uptake, completion	Those who received a provider recommendation in the intervention were 4x significantly more likely to be vaccinated, but not more likely to complete the series.

**Results indicate intervention not associated HPV vaccine outcomes**							
Marchand-Ciriello et al., 2020 [34]	United States<br><br>Pediatric practices in Northeastern Massachusetts	N = 520<br>Boys<br>11-21 years	Race = NR<br><br>Health insurance<br>Private = 50-57%<br>Public = 43-50%	N = 3 practices<br><br>Parent knowledge/attitudes, provider education, provider communication/ recommendation, vaccine procedures, provider prompts	Parents received culturally competent, bilingual, HPV educational information at check-in for all medical appointments. Providers participated in a culturally-sensitive educational program focused on recommendation. Vaccine procedures implemented a bundled recommendation with routine adolescent vaccines to all 11 and 12 year old patients. Electronic provider prompts were created.	Initiation, Uptake of dose 2 and 3	The intervention was not significantly associated with initiation or completion of the series.

N = sample size; NR = not reported; NH = non-Hispanic; SES = socioeconomic status; HPV = human papillomavirus.
High school diploma may also include completion of GED equivalency diploma.
*Based on parent, adolescent, or practice data reported.
